# Multimodal subtypes identified in Alzheimer’s Disease Neuroimaging Initiative participants by missing-data-enabled subtype and stage inference

**DOI:** 10.1093/braincomms/fcae219

**Published:** 2024-06-25

**Authors:** Mar Estarellas, Neil P Oxtoby, Jonathan M Schott, Daniel C Alexander, Alexandra L Young

**Affiliations:** Centre for Medical Image Computing, Department of Computer Science, University College London, London, UK; School of Biological and Behavioural Sciences, Queen Mary University of London, London, UK; Centre for Medical Image Computing, Department of Computer Science, University College London, London, UK; Dementia Research Centre, UCL Queen Square Institute of Neurology, London, UK; Centre for Medical Image Computing, Department of Computer Science, University College London, London, UK; Centre for Medical Image Computing, Department of Computer Science, University College London, London, UK; Department of Neuroimaging, Institute of Psychiatry, Psychology and Neuroscience, King’s College London, London, UK

**Keywords:** Alzheimer’s disease, subtyping, heterogeneity, missing data, multimodal

## Abstract

Alzheimer’s disease is a highly heterogeneous disease in which different biomarkers are dynamic over different windows of the decades-long pathophysiological processes, and potentially have distinct involvement in different subgroups. Subtype and Stage Inference is an unsupervised learning algorithm that disentangles the phenotypic heterogeneity and temporal progression of disease biomarkers, providing disease insight and quantitative estimates of individual subtype and stage. However, a key limitation of Subtype and Stage Inference is that it requires a complete set of biomarkers for each subject, reducing the number of datapoints available for model fitting and limiting applications of Subtype and Stage Inference to modalities that are widely collected, e.g. volumetric biomarkers derived from structural MRI. In this study, we adapted the Subtype and Stage Inference algorithm to handle missing data, enabling the application of Subtype and Stage Inference to multimodal data (magnetic resonance imaging, positron emission tomography, cerebrospinal fluid and cognitive tests) from 789 participants in the Alzheimer’s Disease Neuroimaging Initiative. Missing-data Subtype and Stage Inference identified five subtypes having distinct progression patterns, which we describe by the earliest unique abnormality as ‘Typical AD with Early Tau’, ‘Typical AD with Late Tau’, ‘Cortical’, ‘Cognitive’ and ‘Subcortical’. These new multimodal subtypes were differentially associated with age, years of education, Apolipoprotein E (APOE4) status, white matter hyperintensity burden and the rate of conversion from mild cognitive impairment to Alzheimer’s disease, with the ‘Cognitive’ subtype showing the fastest clinical progression, and the ‘Subcortical’ subtype the slowest. Overall, we demonstrate that missing-data Subtype and Stage Inference reveals a finer landscape of Alzheimer’s disease subtypes, each of which are associated with different risk factors. Missing-data Subtype and Stage Inference has broad utility, enabling the prediction of progression in a much wider set of individuals, rather than being restricted to those with complete data.

## Teaser

Estarellas *et al*. report that an adapted Subtype and Stage Inference algorithm accommodates missing data in multimodal Alzheimer’s disease studies. They identified five distinct subtypes, each associated with different risk factors, revealing new insights into the disease’s landscape and progression.

## Graphical Abstract

**Graphical Abstract:**
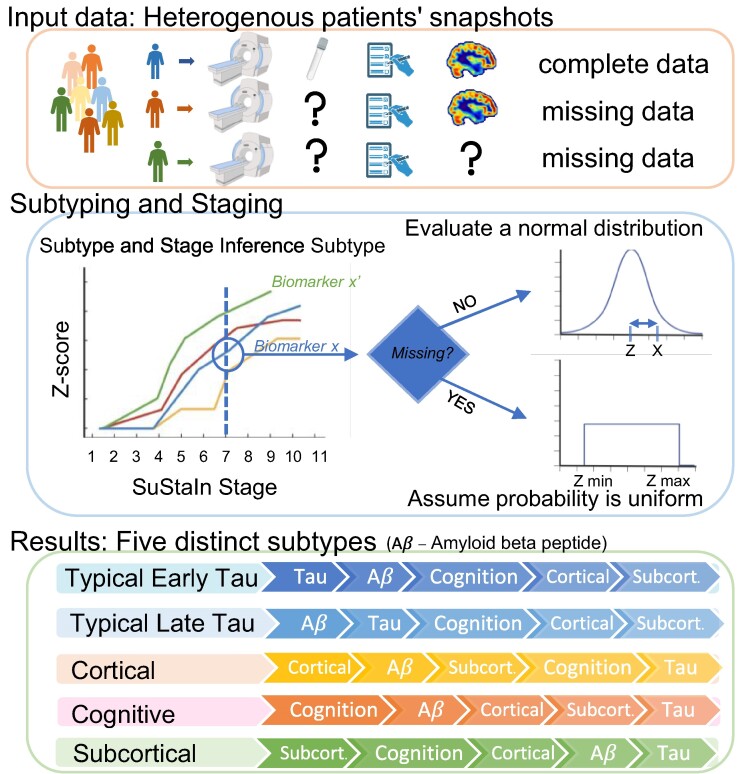

## Introduction

Alzheimer’s disease is clinically and pathologically heterogeneous. This heterogeneity potentially has important implications for clinical trials as heterogeneity may mask the benefit of a treatment that is effective in a particular subgroup.^1,2^ This motivates recent efforts to unravel the heterogeneous temporal progression patterns of Alzheimer’s disease using disease biomarkers and their relationship with clinical presentation, genetics, risk factors and multi-morbidity;^1-8^ this in turn potentially enables stratification for more targeted clinical trials and assisting clinicians in patient management.^9^

Established Alzheimer’s disease biomarkers include CSF^10^ and PET imaging of amyloid and tau accumulation;^11,12^ MRI of regional brain atrophy;^13^ and cognitive test scores.^14^ As each of these modalities provide different information and are likely dynamic at different stages,^15^ integrating information from a range of different disease biomarkers is key to building a complete picture of Alzheimer’s disease.^16,17^

Data-driven computational models enable the construction of a quantitative picture of the phenotypic and temporal heterogeneity of Alzheimer’s disease. However, most studies focus either on temporal (staging)^15,18-20^ or phenotype (subtyping) differences,^4,21^ which risks conflating disease subtypes with disease stages. Subtype and Stage Inference (SuStaIn)^3^ is an unsupervised machine learning algorithm that uses clustering and data-driven disease progression modelling techniques to identify subgroups of individuals with distinct disease trajectories. This has the advantage of simultaneously disentangling phenotypic heterogeneity (the presence of different disease subtypes) and temporal progression (different stages of the disease). The algorithm requires only cross-sectional data but can use longitudinal data if available. To date, SuStaIn applications have mostly exploited single-modality datasets, such as MRI or PET,^3,22^ or occasionally two data types such as Tau CSF and PET together.^23^ SuStaIn models built on more diverse sets of markers would provide a more complete picture of the disease time course and landscape of subtypes.^16,17^

One of the major challenges when applying SuStaIn to multimodal data is ‘missing data’, as many study subjects miss one or more modality due to refusal or funding constraints.^24^ Whilst there are several different versions of SuStaIn that enable the use of a range of disease progression models for different data types,^25-28^ none are well suited to modelling multimodal biomarker data. The most widely used version of the SuStaIn algorithm is *Z*-score SuStaIn.^3^  *Z*-score SuStaIn is the most appropriate version of SuStaIn to use for most modalities as it models the continuous evolution of biomarkers from one *Z*-score to another, capturing the gradual change of biomarkers with disease progression. However, *Z*-score SuStaIn currently requires complete data for all subjects. Alternative versions of SuStaIn, such as event-based SuStaIn^25,26,28^ and ordinal SuStaIn,^27^ more readily handle missing data but are limited in the types of progression and data they can model; event-based SuStaIn can only model discrete transitions from a normal to an abnormal level, whilst ordinal SuStaIn can only model discrete scored data such as visual or neuropathological ratings data.

Here we propose an adaptation of *Z*-score SuStaIn to allow for missing data, which we refer to as missing-data SuStaIn. We first validate our implementation of missing-data SuStaIn using a synthetic dataset. We use missing-data SuStaIn to identify subtypes with distinct progression patterns using multimodal data including PET, CSF, MRI and cognitive scores from the Alzheimer’s Disease Neuroimaging Initiative (ADNI) dataset. We then evaluate the associations between each subtype and demographics, cognitive scores, white matter hyperintensity volumes and age at death. Finally, we test whether the multimodal subtypes and stages identified by missing-data SuStaIn provide clinical utility for predicting conversion from mild cognitive impairment (MCI) to Alzheimer’s disease.

## Materials and methods

### Subtype and Stage Inference

To estimate subgroup progression patterns, SuStaIn^29^ simultaneously clusters individuals into groups (subtypes) and uses disease progression modelling to reconstruct a disease progression pattern (set of stages) for each subgroup. *Z*-score SuStaIn models disease progression using a piecewise linear *Z*-score model. The piecewise linear *Z*-score model describes disease progression as a series of stages, where each stage corresponds to a new biomarker reaching a new *Z*-score. The *Z*-score SuStaIn algorithm then consists of simultaneously optimizing subtype membership, and stage progression, building on the well-established methods developed for the event-based model^25,26^ and incorporating ideas from clustering. SuStaIn evaluates each subject’s likelihood of belonging to each subtype and stage, outputting the probability each subject belongs to each subtype and stage together with their maximum likelihood subtype and stage assignment.

The missing data adaptation we propose requires modification of the data likelihood. The data likelihood P(X|M) for the *Z*-score model in SuStaIn (derived in detail in Young *et al*.^3^) can be written as:


(1) $$P(X|M)=\;\prod_{\;j\;=\;1}^{J}\;\left(\sum_{c=1}^{C}\;f_{c}\sum_{k\;=\;0}^{N}\;\left[\int_{t\;=\;\frac{k}{N+1}}^{t\;=\;\frac{k+1}{N+1}}\left(P(t)\prod_{i\;=\;1}^{I}\;P(x_{ij}|t)\right)\partial t\right]\right)$$

where $x_{ij}\;$ is the measurement of biomarker *i* in subject *j*, *j* = *1* … *J*. *C* is the number of clusters (subtypes), *f* is the proportion of subjects assigned to a particular cluster (subtype), and M is the overall SuStaIn model; *k* refers to disease stage, *N* number of stages and *P*(*t*) is the prior likelihood of being at stage *k*. $P(x_{ij}|t)$ is usually modelled as a normal distribution around the piecewise linear trajectory $g_{i}(t)$ with variance $\sigma_{i}$ estimated from a control population:


(2) $$P(x_{ij}|t)=\mathrm{NormPDF}\;(x_{ij},\;g_{i}(t),\;\sigma_{i}).$$

The piecewise linear trajectory is described by an ordering of a set of *N Z*-score events $E_{\left\{iZ\right\}},\;\mathrm{where}\;\mathrm{each}\;\mathrm{event}\;$ corresponds to the linear increase of a biomarker i=1…I to a *Z*-score $\;Z_{\left\{i1\right\}}\ldots \;Z_{\left\{iR_{i}\right\}}$. Each biomarker trajectory is parameterized to start at Z=0 and end at $Z=Z^{\max}$.

### Missing data adaptation

The strategies for dealing with missing data are described below. Our proposed approach, which we label *MD1*, was benchmarked against alternative pre-processing approaches that handle missing data by deleting or inferring missing values of $x_{ij}$, *MD2-4*.

#### MD1: uniform

We handle missing data in SuStaIn by modelling $P(x_{ij}$|*t*), in the absence of $x_{ij}$, and evaluating Eq. (1) using


(3) $$P(x_{ij}|t)=\{\begin{array}{c} \mathrm{NormPDF}(x_{ij},\;g_{i}(t),\;\sigma_{i}),\;\;x_{ij}\;\mathrm{is}\;\mathrm{present}\;\\ \;\;\;\;\;\;P^{\prime }(x_{ij}|t)\;,\;\;\;\;x_{ij}\;\mathrm{is}\;\mathrm{missing} \end{array}.$$

When $x_{ij}\;\mathrm{is}\;\mathrm{missing}$, we propose modelling the distribution of $x_{ij}$ as uniform over the range of the *Z*-scores for that biomarker, so that$\;P^{\prime }\left(x_{ij}|t\right)=\;\frac{1}{Z_{i}^{\max}}$. This strategy means that missing biomarker entries have no effect on the overall progression pattern estimated by SuStaIn, whilst still enabling the available biomarker entries for each participant to contribute to the subtype and stage estimation.

This adaption can be used in both the training phase—estimating the subtypes and trajectories—and the application phase—assigning subtypes and stages to individuals. The Python implementation of the SuStaIn algorithm (pySuStaIn) was adapted to enable subtype progression patterns to be estimated and to allow subtyping and staging of individuals from incomplete data. The implementation can be found in the pySuStaIn package^29^ and is available on GitHub (https://github.com/ucl-pond/pySuStaIn).

#### MD2: deletion

All subjects with one or more missing biomarkers are excluded from the dataset, as in previous applications of *Z*-score SuStaIn.

#### MD3: imputing the mean

This approach imputes the missing datapoint as the mean of that biomarker over all subjects with non-missing values, i.e. $x_{ij}=\;\frac{1}{J}\;\overset{J}{\underset{i=1}{\sum }}\;x_{ij}$ (where $x_{ij}\;$ is the measurement of biomarker *i* in subject *j*, *j* = *1* … *J*). It is computationally fast but has clear disadvantages such as reducing variance in the dataset.

#### MD4: imputation using K-nearest neighbours

This approach imputes the missing datapoint as the mean value of that biomarker over a set of K similar subjects, who are identified using K-nearest neighbours (KNN). KNN is a supervised learning algorithm that finds the K-nearest subjects using a distance metric.^30^ As the modalities we wish to impute vary considerably in magnitude, we computed the distance between subjects using two different subject level feature vectors: (i) the mean, range, and standard deviation of an individual’s biomarker values; and (ii) an individual’s *Z*-scored biomarker data. Since the biomarkers can vary significantly in terms of magnitude, using this feature vector ensures that the algorithm can account for the overall statistical distribution (mean, range, standard deviation). Now, each subject is represented by three dimensions. Using these dimensions, we can compute the distance between subjects. After establishing the distance metrics, we refer to the original matrix to inspect the actual biomarker values for the subject with the missing data. We then impute this missing value by calculating the mean of these observed biomarker values. In summary, the following iterative procedure based on a simple KNN algorithm was computed, for each missing biomarker value for each subject:

1. In the feature vector extracted (composed of either the mean, standard deviation, and range for each subject, or the *Z*-scored data), find the K subjects that have the most similar features, using Euclidean distance between the two vectors being compared.
2. Go to the original data matrix and find the values of the missing biomarker from those K most similar subjects.
3. Compute the average of such value and replace the missing biomarker value.

### Datasets

#### Synthetic dataset

A synthetic dataset of 500 subjects and 10 biomarkers was generated to test different approaches to handling missing data. The data were simulated as in the original SuStaIn paper.^3^ The default number of clusters was set to *C* = 3, and no biomarker covariance, setting Σ to the identity matrix. SuStaIn stages were simulated using a uniform distribution and SuStaIn subtypes, using the following fraction: $f_{c}\;=\;\frac{2\;+\;(C\;-\;c)}{2C\;+\;\sum_{C\;=\;1}^{C}\;(C\;-\;c)}$. Thus, the fraction of subjects belonging to each cluster are $f_{1}=\frac{4}{9}\;,\;\;f_{2}=\frac{3}{9}\;,\;\;f_{3}=\frac{2}{9}.\;\;$ The progression pattern for each cluster is simulated as a linear *Z*-score model with a random monotonic ordering of the *Z*-score events, fixing *Z_i_* = (1,2,3) and $Z_{i}^{\max}$ = 5 for all biomarkers *i*. From this dataset, 150 and 2000 random values (3% and 40% of the 5000 datapoints) were deleted to mimic missing values. The resulting datasets were used to compare different approaches to handling missing data. We benchmarked the performance of each approach to handling missing data both against each other and against performance on the full dataset.

#### ADNI dataset

Data used in the preparation of this article were obtained from the ADNI database (adni.loni.usc.edu). The ADNI was launched in 2003 as a public–private partnership, led by Principal Investigator Michael W. Weiner, MD. The primary goal of ADNI has been to test whether serial MRI, PET, other biological markers, and clinical and neuropsychological assessment can be combined to measure the progression of MCI and early Alzheimer’s disease. For up-to-date information, see www.adni-info.org. Written consent was obtained from all participants, and the study was approved by the Institutional Review Board at each participating institution.

The inclusion criteria for our study were the availability of cross-sectional Freesurfer volumes derived from a 3T MRI scan at baseline and that these volumes passed overall quality control. Nine follow-up visits (up to Month 42 according to ADNI’s records) were also used for experiments in this work (see Supplementary Analysis—Longitudinal Consistency). The resulting dataset consisted of 789 subjects [182 cognitively normal (CN), 86 significant memory concern (SMC), 241 early MCI, 163 late MCI and 117 Alzheimer’s disease]. For this study, ADNI diagnosis was divided into three categories: controls (CN), Alzheimer’s disease and MCI. The group of controls includes subjects diagnosed as healthy and those with subjective memory complaints. Late and early MCIs were grouped together as MCI. The downloaded Freesurfer values were used to compute the volume of six cortical regions (frontal lobe, temporal lobe, occipital lobe, parietal lobe, cingulate, insula) and four subcortical regions (hippocampus, amygdala, thalamus and basal ganglia formed by the accumbens, pallidum, putamen and caudate).

Several additional biomarkers were downloaded comprising measurements from CSF, PET and cognitive tests. CSF measurements included Aβ, tau and p-tau, for which 109 subjects had missing data. PET measures of florbetapir (18F-AV45)-PET averaged of angular, temporal and posterior cingulate; and fluorodeoxyglucose (FDG) mean of whole cerebellum, were downloaded from the ADNIMERGE spreadsheet. 86 subjects had missing FDG-PET data and 92 subjects missing AV45 data. Three cognitive test scores were selected from the ADNIMERGE spreadsheet/table: the Alzheimer’s Disease Assessment Scale-Cognitive Subscale (ADAS-Cog 13),^31^ Rey Auditory Verbal Learning Test—RAVLT (RAVLT immediate sum of five trials)^32^ and the time to complete the Trail Making Test (TRABSCOR),^33^ for which only 33 subjects had missing entries. Finally, demographic data (age, sex, education), APOE genotype and total intracranial volume were also downloaded from ADNIMERGE table for covariate correction. Overall, baseline measurements had a total of 3.8% of the data missing, of which 60.8% was CSF data, 33% corresponded to missing PET and 6.1%, cognitive data.

### Statistical analysis

#### Evaluation of missing data modelling using synthetic data

The synthetic datasets enable evaluation of subtype and stage estimates against known ground truth values. To select the most effective strategy to deal with missing data, we evaluate the results obtained with the different methods. The event sequence similarity between two subtype progression patterns was computed using the Kendall tau distance between the sequence of the biomarkers obtained with each approach versus the ground truth sequence. Subtyping accuracy was reported as the percentage of subjects who were subtyped correctly (the estimated subtype coincided with the ground-truth subtype). Finally, the accuracy of patient staging is reported per subtype as the mean error ± standard deviation (across individuals) between the estimated stage and the ground-truth stage.

#### SuStaIn modelling in ADNI

All biomarkers were corrected for age, sex, education and, in the case of imaging markers, total intracranial volume. The correction was performed by estimating a linear regression model in a control population of 89 amyloid-negative CN participants (CSF Aβ1-42 > 192 pg/ml^34^), and then propagating this model to all 789 participants for the subset of associations that were significant. Baseline data of the corrected biomarkers were then converted into *Z*-scores relative to the control population for use as input to SuStaIn. *Z*-scores of up to 5 were used in this study (1, 2, 3 and 5). The minimum biomarker *Z*-score was set to zero for all biomarkers, whilst the maximum was set to the rounded 95th percentile computed for each biomarker. Biomarkers that decrease with disease progression were multiplied by −1 to give positive *Z*-scores that increase with disease progression. Longitudinal data used in the Supplementary Analysis of Longitudinal Consistency were *Z*-score transformed and covariate corrected using the same method performed in the baseline data.

SuStaIn was applied to three different data subsets: (i) all 789 subjects; (ii) β-amyloid-positive individuals, *n* = 406, (Aβ+); and (iii) β-amyloid-negative individuals, *n* = 275 (Aβ−) based on CSF cut-offs according to.^34^ The optimal number of subtypes was chosen by comparing the distribution of model likelihoods. The individual subject subtype assignments under each model were compared using a Sankey diagram, a typical diagram used to visualize the directed flow between nodes or different sets of values.

Differences between subtypes in age, years of education, sex, ADNI diagnosis and number of APOE4 alleles were studied using a *t*-test for continuous variables or chi-square test for discrete variables. Memory and executive function were also compared between subtypes, computed using the Memory and Executive Function Cognitive battery from the ADNI-composite scores,^35,36^ as were white matter hyperintensity values (WMHV).^37^

#### Predictive utility of SuStaIn

Cox proportional hazard models were used to assess the predictive utility of SuStaIn for predicting conversion from MCI to Alzheimer’s disease. Age, gender, APOE4 status and SuStaIn stage were used as covariates. The risk of MCI to Alzheimer’s disease conversion was assessed using hazard ratios for SuStaIn subtype and stage. A ratio of 1 means no modification of the risk of MCI to Alzheimer’s disease conversion, a ratio > 1 means increase of risk and ratio < 1, decrease of risk.

To further validate missing data SuStaIn and its contribution to clinical practice, the Cox proportional hazard models were fitted to three groups: (i) all subjects; (ii) subjects with complete data only; and (iii) subjects with missing data only. Results were compared using the 95% confidence interval (CI) of resulting hazard ratios for subtype.

### Supplementary analysis

#### Benchmarking modalities

We assessed the importance of different modalities for subtyping and staging of individuals by calculating the consistency of subtypes and stages when artificially treating a modality as missing versus using their full data as a proxy ground truth.

#### Longitudinal consistency

The consistency of subtyping and staging in longitudinal data was also compared for different percentages of missing data. Follow-up subtype assignments were deemed longitudinally consistent if: a participant progressed to a subtype from ‘normal appearing’ (i.e. no evidence of biomarker abnormality and therefore not subtypable); or if they were assigned to the same subtype at follow-up. Follow-up stages were deemed longitudinally consistent if a participant remained at the same stage or progressed to a later stage at follow-up. The 95% CI was computed by determining the set of subtypes and stages that fell within a cumulative probability of 0.95.

## Results

### Validation of missing data SuStaIn using a synthetic dataset

Table 1 shows that the uniform model of *P*(*x_ij_|t*) optimizes performance in recovering subtype trajectories in synthetic data experiments. For 3% data missing, the uniform distribution approach consistently resulted in the highest correlation between the estimated and ground truth progression patterns measured by Kendall Tau rank correlation coefficient (0.93, 0.97 and 0.89 for clusters 1, 2 and 3, respectively). This approach also produced the most accurate subtyping and staging, where 85% of the subjects were classified correctly compared to the ground-truth subtypes; and there was a mean difference of 1.53 (±1.30) stages across individuals. The uniform distribution approach gave similar results when the proportion of missing data was increased to 40%. Consequently, the uniform distribution approach, which we term ‘missing data SuStaIn’, was used for all further analyses.

**Table 1:** Assessment of performance of different methods for handling missing data

Missing data algorithm	Progression pattern similarity Mean Kendall tau distance	Subtype % correct	Stage Mean diff (SD)
3% data missing			
Deletion	0.89	79.60	1.84 (1.64)
Mean	0.92	84.20	1.57 (1.32)
KNN biomarkers (feature matrix)	0.91	84.80	1.60 (1.36)
KNN biomarkers (*Z*-scored)	0.44	60.60	14.25 (7.54)
Uniform distribution	0.93	85.40	1.53 (1.30)
40% data missing			
Deletion			
Mean	0.69	68.80	2.39 (3.39)
KNN biomarkers (feature matrix)	0.68	46.80	2.44 (2.18)
Uniform distribution	0.86	75.20	2.22 (2.06)
Full data	0.92	85.80	1.54 (1.31)

Comparison metrics include the similarity between the estimated subtype progression patterns and the ground truth, and subject staging and subtyping accuracy. Similarity between the three progression patterns is measured using the Kendall Tau coefficient, with 0 indicating no similarity between the progression patterns and 1 indicating identical progression patterns. The last experiment, ‘Full data’, benchmarks the performance when there are no missing data to indicate the best possible performance.

### Multimodal subtypes identified by SuStaIn

**Figure 1:**
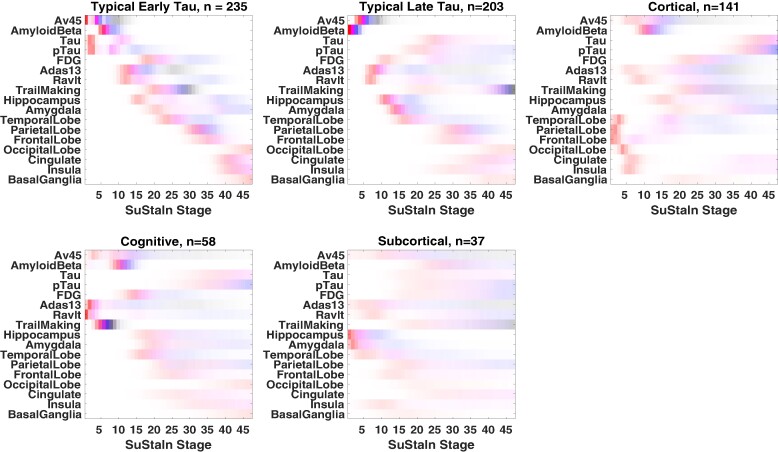
**SuStaIn subtypes identified in the ADNI dataset.** This figure depicts the five subtypes uncovered by SuStaIn and their respective progression patterns. At each stage, the colour indicates the level of change compared to controls: white means no change; red means a change of *Z*-score = 1; magenta, *Z*-score = 2; blue, *Z*-score = 3 and black, *Z*-score = 5. The *y*-axis shows the different biomarkers used for the study, whilst the *x*-axis shows the position of the *Z*-score events for each biomarker, which ranges from 1 to 45. Insula, Amygdala, Hippocampus, Thalamus, FrontalLobe, ParietalLobe, TemporalLobe, OccipitalLobe, Cingulate and BasalGanglia correspond to MRI measurements. Amyloid Beta, Tau and phosphorylated tau (pTau) are CSF markers, whilst fluorodeoxyglucose (FDG) and 18F-AV-45 (Florbetapir F-18 AV45) correspond to PET data. The Alzheimer’s Disease Assessment Scale (ADAS13), the Rey Auditory Verbal Learning Test (RAVLT) and the Trail Making Test (TRABSCOR) are the three cognitive test scores used in this study. *N* corresponds to the number of subjects belonging to each subtype, excluding those that were assigned to the Normal Appearing subtype (SuStaIn stage 0).

Figure 1 shows the temporal progression of the five different subtypes identified by missing data SuStaIn when applied to all 789 subjects from ADNI (Aβ+ and Aβ−). We labelled these five different subtypes as: ‘Typical AD Early Tau’, ‘Typical AD Late Tau’, ‘Cortical’, ‘Cognitive’ and ‘Subcortical’ according to early characteristic features of each progression pattern, however we note that the subtypes will have different profiles at different stages as they model temporal progression.

Two hundred thirty-five subjects were assigned to the ‘Typical AD Early Tau’ subtype. This subtype was characterized by an early CSF tau, p-tau and PET AV45 change, followed by CSF Aβ and cognitive decline. MRI volume changes were seen to appear late for this group of subjects. The ‘Typical AD Late Tau’ subtype (203 subjects) had a very similar progression pattern to the ‘Typical AD Early Tau’ subtype; however, in this case, the CSF markers of tau and p-tau appear later, after MRI markers change. The ‘Cortical’ and ‘Cognitive’ subtypes (*n* = 141 and 58, respectively) were characterized by early cortical atrophy and early cognitive decline, respectively. Only 37 subjects were assigned to the ‘Subcortical’ subtype, which shows an uncertain progression pattern with early subcortical atrophy. This suggests the subcortical subtype comprises a collection of individuals with heterogeneous patterns that have common atrophy in subcortical regions such as the amygdala and hippocampus.

**Figure 2:**
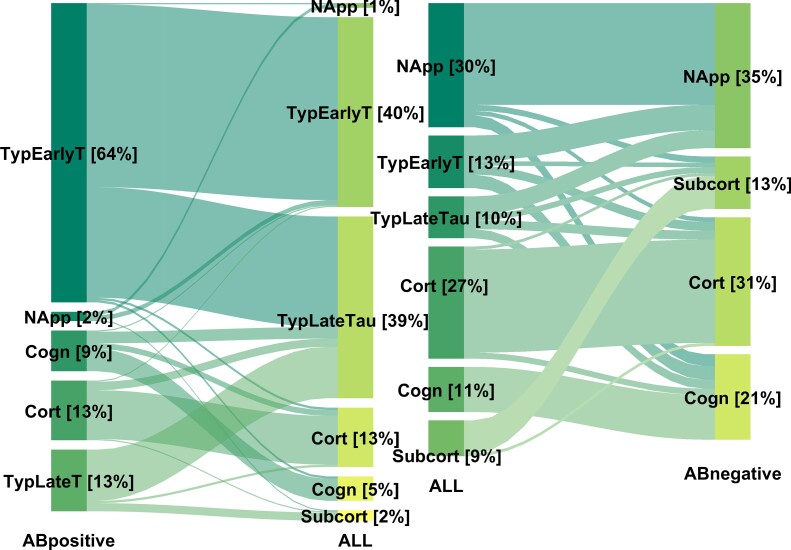
**Two Sankey flows showing subtyping consistency between models.** A Normal Appearing subtype was included, consisting of those subjects assigned to stage 0. Diagram **A** compares the 406 Aβ+ subjects, subtyped by the Aβ+ (left) and full data model (right). Diagram **B** compares the 275 Aβ− subjects, subtyped by the Aβ− (right) and full data model (left).

Missing-data SuStaIn was also applied to only amyloid-positive (Aβ+) (Supplementary Fig. 1) and amyloid-negative (Aβ−) (Supplementary Fig. 2) ADNI subjects. Figure 2A shows a Sankey diagram comparing the subtype assignments of Aβ+ individuals based on subtype progression patterns (i) learnt in the whole population and (ii) learnt in only Aβ+ individuals. Similarly, Fig. 2B shows a Sankey diagram comparing the subtype assignments of Aβ− individuals based on subtype progression patterns (i) learnt in the whole population and (ii) learnt in only Aβ− individuals. Application of SuStaIn to Aβ+ subjects recovered four of the five subtypes: ‘Typical AD Early Tau’, ‘Typical AD Late Tau’, ‘Cortical’ and ‘Cognitive’, whereas application of SuStaIn to Aβ− subjects recovered three of the five subtypes: ‘Cortical’, ‘Cognitive’ and ‘Subcortical’. Thus, ‘Typical AD Early Tau’ and ‘Typical AD Late Tau’ subtypes were unique to the Aβ+ group, whereas the ‘Subcortical’ subtype appeared only in Aβ− subjects.

Results in the following sections were computed using the SuStaIn model learnt from all 789 subjects, unless stated.

### Relationship of subtypes with demographics and risk factors

**Figure 3:**
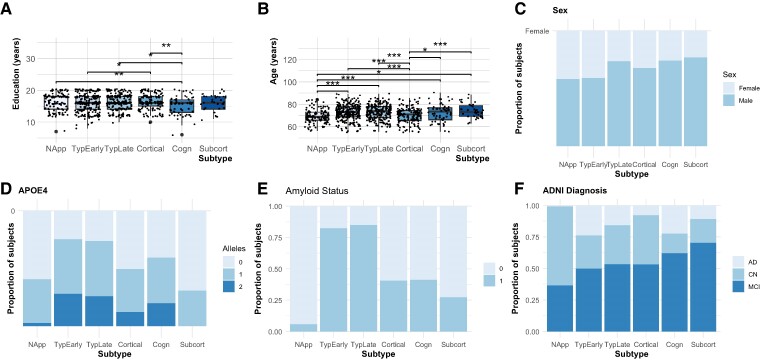
**A–F show plots depicting the differences in education, age, gender, diagnosis, APOE status and stage between SuStaIn subtypes.** A Normal Appearing subtype was added in all experiments, representing those people in stage zero. (**A**) Boxplots showing the distribution of education years in all different subtypes. (**B**) Boxplot depicting the age distribution in subtypes. Significant differences between groups computed via *t*-tests are marked with three stars for a *P*-value < 0.001, two stars for *P*-value < 0.01 and one star for *P*-value < 0.05. (**C**) Bar plot showing the proportion of female and male subjects in every subtype. A significantly higher number of males were observed between ‘Normal Appearing’ versus ‘Typical Late Tau’ (*P*-value = 0.044), ‘Subcortical’ (*P*-value = 0.03) and ‘Cognitive’ (*P*-value = 0.04). ‘Typical Late Tau’ versus ‘Typical Early Tau’ (*P*-value = 0.0008). ‘Typical Early Tau’ versus ‘Subcortical’ (*P*-value = 0.031), and versus ‘Cognitive’ (*P*-value = 0.029). (**D**) Bar plot depicting the proportion of subjects with zero, one or two APOE4 alleles in each subtype. Significant differences were found between ‘Normal Appearing’ and both ‘Typical Late Tau’ (*P*-value < 0.0001) and ‘Typical Early Tau’ (*P*-value < 0.0001). ‘Typical Late Tau’ showed significant differences with ‘Cortical’ (*P*-value < 0.0001), ‘Subcortical’ (*P*-value < 0.0001) and ‘Cognitive’ subtypes, with the ‘Cortical’ and ‘Typical Early Tau’ pair also showing a significant correlation (*P*-value < 0.0001). (**E**) Bar plot showing the proportion of amyloid-positive subjects in each subtype, where = were significant differences were found between ‘Normal Appearing’ and ‘Typical Late Tau’ (*P*-value < 0.0001), ‘Cortical’ (*P*-value = 0.002), ‘Typical Early Tau’ (*P*-value < 0.0001) and ‘Cognitive’ (*P*-value = 0.01247). ‘Typical Late Tau’ versus ‘Cortical’ (*P*-value < 0.0001), and ‘Subcortical’ (*P*-value < 0.0001), ‘Cognitive’ (*P*-value < 0.0001). ‘Cortical’ versus ‘Typical Early Tau’ (*P*-value < 0.0001). ‘Typical Early Tau’ versus ‘Subcortical’ (*P*-value < 0.0001), and ‘Cognitive’ (*P*-value < 0.0001). (**F**) This bar plot shows the proportion of subjects diagnosed as MCI, Alzheimer’s disease or CN in ADNI; divided by subject. Significantly more subjects diagnosed with Alzheimer’s disease were found between ‘Normal Appearing’ and ‘Typical Late Tau’ (*P*-value < 0.0001), ‘Cortical’ (*P*-value = 0.02), ‘Typical Early Tau’ (*P*-value < 0.0001), ‘Subcortical’ (*P*-value = 0.015) and ‘Cognitive’ (*P*-value < 0.0001). ‘Typical Late Tau’ versus ‘Typical Early Tau’ (*P*-value = 0.047), and ‘Cortical’ (*P*-value = 0.042). ‘Cortical’ versus ‘Typical Early Tau’ (*P*-value < 0.0001), and versus ‘Cognitive’ (*P*-value = 0.008). Statistics for **C–F** were computed using chi-square test. For a complete description of statistics results, please refer to Supplementary Table 3.

Figure 3 and Table 2 show the demographic variables of education, age, gender, APOE4 status, amyloid positivity and ADNI diagnosis for each SuStaIn-assigned subtype. Subjects assigned to the ‘Cognitive’ subtype had a mean of 1.1 fewer years of education than all other subtypes, this difference being significant when compared to the ‘Normal Appearing’ (*P*-value = 0.005 cognitive versus normal appearing *t*-test), ‘Typical Late Tau’ (*P*-value = 0.01 cognitive versus typical late tau *t*-test) and ‘Cortical’ (*P*-value = 0.02 cognitive versus cortical *t*-test) subtypes. Individuals with the ‘Cortical’ subtype were on average 3.4 years younger than any other subtype (*P*-values < 0.001 versus typical early tau, typical late tau, cognitive and subcortical, respectively, *t*-test); and those assigned to the ‘Subcortical’ subtype were the oldest (mean 1.93 years; *P*-values < 0.001 versus typical early tau, typical late tau, cognitive and cortical, respectively, *t*-test). Significantly more females were found in the ‘Normal appearing’ subtype (4.9% more females; *P*-values < 0.05 versus typical late tau, cognitive, cortical and subcortical, respectively, chi-square test) and the ‘Typical Early Tau’ subtype (5.5% more females; *P*-values < 0.05 versus typical late tau, cognitive, cortical and subcortical, respectively, chi-square test). The proportion of subjects who were APOE4 positive (one or more APOE4 alleles) and with ADNI diagnosis of Alzheimer’s disease was significantly larger in the two Typical Alzheimer’s disease subtypes and the Cognitive subtype (*P*-values < 0.02 versus cortical and subcortical, respectively, chi-square test).

**Table 2:** Variable of age, education, sex, APOE4 alleles, amyloid status and ADNI diagnosis divided by SuStaIn subtype

Subtypes → Variables ↓	Normal Appearing (115)	Typical AD Early Tau (235)	Typical AD Late Tau (203)	Cortical (141)	Cognitive (58)	Subcortical (37)
Age, years, mean (SD)	69.5 (6.2)*** P-value = 2.69e^−05^	72.7 (7.0)* P-value = 0.02	73.0 (6.8)** P-value = 0.005	69.9 (7.0)** P-value = 0.003	72.3 (7.9)	74.29 (6.3)** P-value = 0.02
Education, years, mean (SD)	16.5 (2.7)	15.9 (2.8)	16.2 (2.5)	16.6 (2.5)** P-value = 0.03	15.2 (2.9)** P-value = 0.009	16.23 (2.4)
Sex, % female	59.1	58.3	41.9	48.9	41.4	37.8
APOE4 alleles, % positive	27.7	56	55.4	30.7	43.8	18.9
Amyloid status, % positive	5.7	82.3	84.9	40.5	41.2	27.3
ADNI diagnosis, % of total *n*						
Alzheimer’s disease	0.8	23.8	15.8	7.8	22.4	10.8
CN	62.6	26.4	31.0	39.0	15.5	18.9
MCI	36.5	49.8	53.2	53.2	62.1	70.3
Cognitive batteries memory function (mean)	1.1*** P = 7.52e^−37^	0.2*** P = 1.81e^−06^	0.3	0.5	−0.1*** P = 7.68e^−08^	0.4
Executive function (mean)	1.1*** P = 7.61e^−21^	0.1*** P = 2.99e^−05^	0.5	0.4	−0.6*** P = 2.94e^−11^	0.6
Total WMHV at baseline, (mean)	3.6** P = 0.01	5.9	7.5	5.9	4.2	8.6
Age at death, [mean, (*n*)]	79 (1)	78 (8)	76.8 (5)	82.2 (4)	83.5 (6)	93 (1)

Statistical significance is computed between each subtype versus all the rest. Significant differences are marked with three stars for a *P*-value < 0.001, two stars for *P*-value < 0.01 and one star for *P*-value < 0.05.

A significantly higher proportion of subjects were amyloid positive in the ‘Typical Late Tau’ and ‘Typical Early Tau’ subtypes, 82% and 85% of the data, respectively (*P*-values < 0.001 versus cognitive, cortical and subcortical *t*-test, respectively). The ‘Subcortical’ subtype had the lowest proportion of amyloid-positive subjects, with only 27% (*P*-values < 0.001 versus typical early tau, typical late tau, cognitive and cortical, respectively, *t*-test). The two Typical Alzheimer’s disease subtypes and the Cognitive subtype had the highest proportion of Alzheimer’s disease diagnosed subjects, with 24% and 22% of Alzheimer’s disease subjects in each subtype. The Cortical and Subcortical subtypes had the lowest proportion of Alzheimer’s disease diagnosed subjects; 8% and 11% (*P*-values < 0.0001 versus typical early tau, typical late tau, cognitive and cortical, respectively, *t*-test). MCI diagnosis was more prevalent in the Subcortical subtype than all other subtypes, with 70% of the data belonging to this diagnosis category (*P*-values < 0.0001 versus typical early tau, typical late tau, cognitive and cortical, respectively, *t*-test).

**Figure 4:**
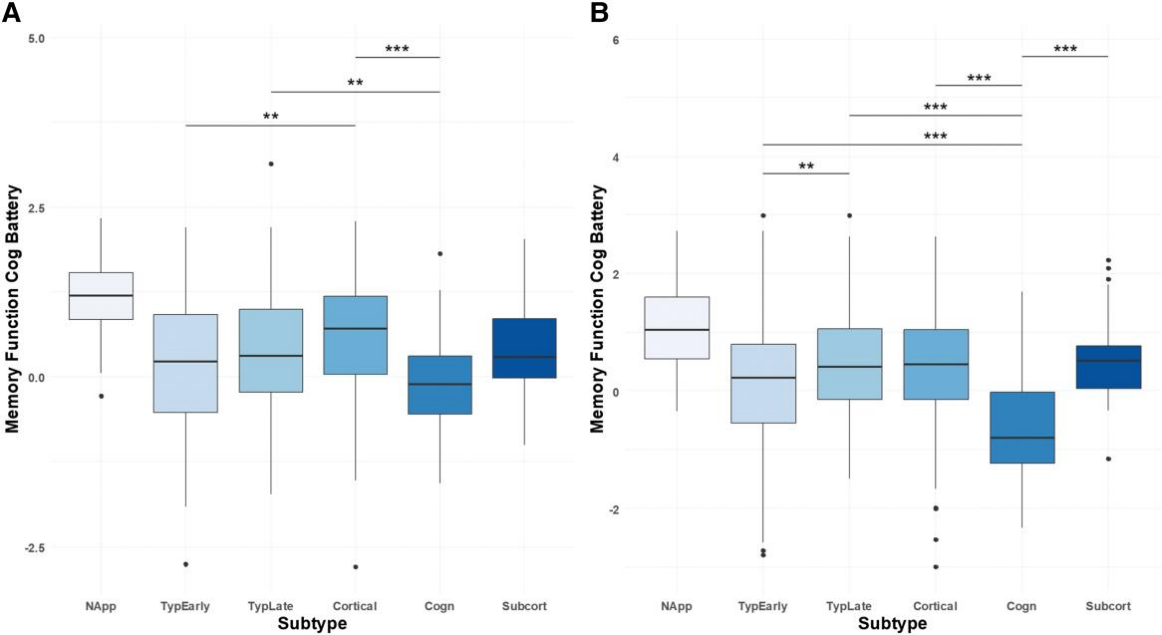
**Boxplots showing subtype differences in two cognitive batteries.** (**A**) Memory Function. Significant differences were found between ‘Normal Appearing’ versus ‘Typical Early Tau’ (t = 12.83; *P*-value < 0.0001), ‘Normal Appearing’ versus ‘Typical Late Tau’ (t-statistic = 10.61; *P*-value < 0.0001), ‘Normal Appearing’ versus ‘Cortical’ (t = 7.68; *P*-value < 0.0001), ‘Normal Appearing’ versus ‘Cognitive’ (t = 12.99; *P*-value < 0.0001) and ‘Normal Appearing’ versus ‘Subcortical’ (t = 6.16; *P*-value < 0.0001). Significant differences were also noted between ‘Typical Early Tau’ and ‘Cortical’ (t = −3.73; *P*-value = 0.0012), ‘Typical Late Tau’ and ‘Cognitive’ (t = 4.07; *P*-value = 0.008) and ‘Cortical’ and ‘Cognitive’ (t = 5.51; *P*-value < 0.0001). (**B**) For executive function, the patterns were similar: ‘Normal Appearing’ versus ‘Typical Early Tau’ (t = 9.61; *P*-value < 0.0001), ‘Normal Appearing’ versus ‘Typical Late Tau’ (t = 6.49; *P*-value < 0.0001), ‘Normal Appearing’ versus ‘Cortical’ (t = 5.98; *P*-value < 0.0001) and ‘Normal Appearing’ versus ‘Cognitive’ (t = 11.70; *P*-value < 0.0001). Additional significant differences were observed between ‘Typical Early Tau’ and ‘Typical Late Tau’ (t = −3.70; *P*-value = 0.002), ‘Typical Early Tau’ and ‘Cognitive’ (t = 4.91; *P*-value ≤ 0.0001), ‘Typical Late Tau’ and ‘Cognitive’ (t = 7.62; *P*-value < 0.0001), ‘Cortical’ and ‘Cognitive’ (t = 6.55; *P*-value < 0.0001) and ‘Cognitive’ versus ‘Subcortical’ (t = −6.53; *P*-value < 0.0001). All reported *P*-values are after Bonferroni correction for multiple comparisons. Significant correlations between ‘Normal Appearing’ and all other subtypes are not depicted in the figure for easier visualization. Subtype abbreviations: NormalApp, Normal Appearing; TypEarlyT, Typical Early Tau; TypLateT, Typical Late Tau.

Figure 4 shows the difference in memory and executive function between subtypes. Cognitive subtype subjects show the worst performance in both cognitive batteries, with −0.1 and −0.6 for memory and executive function, respectively. These scores were significantly lower than all other subtypes (memory function and executive function cognitive battery *P*-value < 0.05 cognitive subtype versus typical early tau, typical late tau, cortical and subcortical *t*-test), except when comparing memory function with the ‘typical Early Tau’ subtype.

### Prediction of conversion

Supplementary Fig. 3 shows that the different SuStaIn subtypes have distinct risks of MCI to Alzheimer’s disease conversion. By fitting a Cox proportional hazard model, we found significant effects of subtype and stage in the risk of progressing from MCI to Alzheimer’s disease, based on ADNI diagnosis labels. Of the SuStaIn subtypes, the ‘Cognitive’ subtype was associated with the highest rate of progression [HR = 12. 82 (4.20–39.1 95% CI) *P*-value < 0.001], whilst the ‘Subcortical’ subtype was associated with the lowest, although non-significant [HR = 1.75 (0.40–7.7) *P*-value = 0.45], ‘Typical AD Early Tau’ and ‘Typical AD Late Tau’ have very similar conversion rate [HR = 6.11 (2.26–16.5) *P*-value < 0.001; and HR = 7.58 (2.83–20.3) *P*-value < 0.001], showing a faster conversion than the ‘Cortical’ and ‘Subcortical’ subtypes, however slower than ‘Cognitive’. SuStaIn stage was also found to be significant [HR = 1.08 (1.06–1.1) *P*-value < 0.001] when computing MCI to Alzheimer’s disease conversion in ADNI data, with each increase in stage corresponding to an 8% increase in the hazard ratio.

### Evaluating the effect of missing data in the prediction of MCI to Alzheimer’s disease conversion

**Figure 5:**
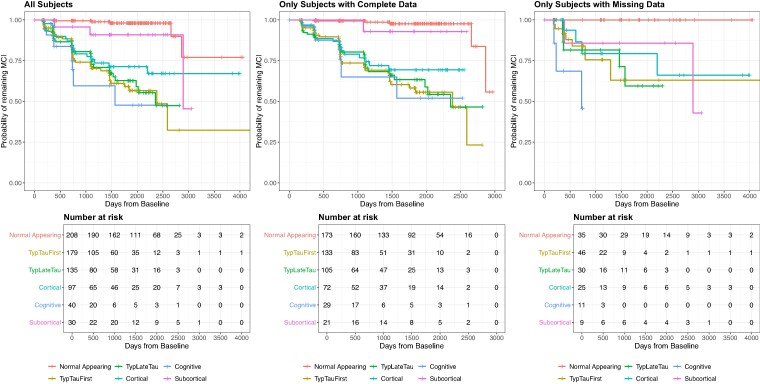
**MCI to Alzheimer’s disease conversion Kaplan–Meier curve for each subtype.** Kaplan–Meier model was fitted with all baseline MCI subjects, only baseline MCI subjects with complete data and only baseline MCI subjects with missing data. The prediction of conversion patterns for each subtype is maintained in the different data subsets.

Figure 5 shows the Kaplan–Meier curves for MCI to Alzheimer’s disease progression, fit to each of three different data subsets: all subjects (number of MCI subjects at baseline, *n* = 689), only subjects with complete data at baseline (*n* = 533) and only subjects with missing data at baseline (*n* = 156). In all data subsets, ‘Cognitive’ showed the fastest progression, followed by ‘Typical Late Tau’ and ‘Typical Early Tau’, which have a very similar rate of progression; then ‘Cortical’, and finally ‘Subcortical’ showed the slowest MCI to Alzheimer’s disease progression. The overall hazard ratios of SuStaIn subtype for each dataset were found to be 1.10 (0.96–1.26 CI) in all MCI subjects, 1.06 (0.90–1.35 CI) in the subset of MCI subjects with complete data and 1.36 (1.00–1.81) in MCI subjects with missing data. The results show no statistical difference between models, suggesting that missing-data SuStaIn produces accurate enough subtype and stage assignments to predict MCI to Alzheimer’s disease conversion when data is missing.

### Evaluating the effect of multimodal data versus MRI only in the prediction of MCI to Alzheimer’s disease conversion

By performing likelihood ratio tests comparing the prediction of MCI to Alzheimer’s disease conversion using only MRI or multimodal data to fit SuStaIn, it was found that using missing-data SuStaIn provides a significantly better fit for the Cox proportional hazard models than using only MRI data (*P* = 2.20 × 10−6). This shows that using multimodal data provides additional information for predicting the risk of conversion from MCI to Alzheimer’s disease.

### Supplementary analyses

#### Benchmarking modalities

Supplementary Fig. 4A suggests that the most important modalities for subtyping are PET/CSF (treated in combination). Supplementary Fig. 4B shows that MRI is the modality that most strongly affects the staging similarity when different biomarkers are removed, leading to a bigger spread of the staging results. Supplementary Table 1 demonstrates that whilst the uncertainty in the subtypes and stages increases when a modality is missing, the CI is representative of the true subtype and stage.

#### Longitudinal consistency

Supplementary Fig. 5 shows that longitudinal subtype assignment is most consistent for individuals with high confidence in subtype assignment at every visit, suggesting that SuStaIn provides meaningful estimates of the overall confidence in the subtype assignments. Among subjects that were assigned to a subtype with a probability of 0.9 or higher, there was baseline to follow-up subtype consistency of 86.3%. Whilst longitudinal subtype consistency decreased with subtype probability, the 95% CIs of the subtype assignments showed a strong correspondence between baseline and follow-up, regardless of the subtype probability; the mean percentage over all visits of subjects in which the CIs overlapped between baseline and follow-up was 100% (Supplementary Table 2). This demonstrates the utility of the CIs of the SuStaIn subtypes.

## Discussion

In this work, we developed and applied missing data SuStaIn to model the temporal and phenotypic heterogeneity of an Alzheimer’s disease enriched cohort, ADNI, using multimodal data. We evaluated alternative methods of handling missing data in SuStaIn, finding the most effective way of treating missing data using a synthetic dataset, which we termed ‘missing-data SuStaIn’. Missing-data SuStaIn may be less biased than imputation approaches such as KNN because it doesn’t make any assumptions about the values of the missing biomarkers. Rather than imputing a value when a biomarker is missing, missing-data SuStaIn simply encodes that there is no information available for that biomarker (by using a probability distribution to indicate that any biomarker value is equally likely). This feature allows missing-data SuStaIn to handle missing data in an unbiased manner, estimating subtype progression patterns and performing subtyping and staging based only on the recorded data entries. We demonstrated that missing-data SuStaIn outperforms alternative methods that impute missing data. Our proposed adaptation enabled SuStaIn to be applied to a multimodal set of biomarkers, uncovering five subtypes: ‘Typical AD Early Tau’, ‘Typical AD Late Tau’, ‘Cortical’, ‘Cognitive’ and ‘Subcortical’, in order of prevalence. The ‘Typical AD Early Tau’, ‘Typical AD Late Tau’, ‘Cortical’ and ‘Cognitive’ subtypes were replicated when running missing-data SuStaIn in amyloid-positive individuals only, whilst the ‘Subcortical’, ‘Cortical’ and ‘Cognitive’ subtypes were also found in amyloid-negative individuals. We found that subjects assigned to each subtype had significantly different demographic profiles and rates of MCI to Alzheimer’s disease conversion, which could potentially inform clinical practice.

‘Typical’ subtypes of Alzheimer’s disease have been repeatedly described in atrophy-only studies^38-40^. By including CSF markers in SuStaIn, we found that the typical subtype subdivided into a typical subtype with early tau abnormalities ‘Typical Early Tau’ and a typical subtype showing a later tau deposition ‘Typical Late Tau’. The ‘Typical Early Tau’ subtype appears to correspond with an archetypical pathological progression of Alzheimer’s disease, where tau starts accumulating in the brain first but doesn’t reach an abnormality threshold until Aβ deposition does.^15^ Similar subtypes have previously been found using both SuStaIn^3,41^ and other data-driven methods.^38,40,42,43^ The ‘Typical Early Tau’ subtype contains a large number of individuals with an Alzheimer’s disease diagnosis, and a high proportion of amyloid-positive and APOE4 positive individuals, reinforcing the correspondence of this subtype to a typical Alzheimer’s disease progression pattern.

The ‘Typical Late Tau’ subtype appears to largely reflect a typical pattern of Alzheimer’s disease except tau deposition happens much later in the disease progression. A similar ‘late tau’ subtype was found by another study that applied SuStaIn to CSF and PET.^23^ Several other works have previously studied the differences between subjects presenting a typical Alzheimer’s disease progression with high or low levels of Tau. These studies found that those with higher concentration of tau were more likely to exhibit an executive phenotype of the disease, rather than amnestic^44-46^. In our study, ‘Typical Early Tau’ and ‘Typical Late Tau’ also showed significant differences in executive function, with subjects assigned to the ‘Typical Early Tau’ subtype (i.e. a higher concentration of tau earlier in the disease) having lower executive cognitive scores (mean difference = −0.35 *P*-value = 0.002). We also found significant differences in total white matter hyperintensity, with the ‘Typical Late Tau’ subtype having significantly higher volumes compared to the ‘Normal Appearing’ group. A higher predominance of white matter hyperintensities in typical Alzheimer’s subtypes has been previously reported,^5,38^ but here we linked these white matter hyperintensities to the late tau group specifically. ‘Typical AD Late Tau’ and ‘Typical AD Tau First’ were shown to have very similar MCI to Alzheimer’s disease progression rates. This progression rate was faster than most of the other subtypes, consistent with previous work.^42,43,47^ However, in this study, we found that the ‘cognitive’ subtype had the fastest rate of conversion.

Subjects assigned to the ‘Cortical’ subtype were younger, had the highest educational level and a low percentage of APOE4 positivity. The ‘Cortical’ subtype subjects also had the best memory function and a slow rate of progression from MCI to Alzheimer’s disease. The ‘Cortical’ subtype had a lower proportion of individuals that were amyloid positive (40.5% compared to 82.3% and 84.9% for the two ‘Typical’ subtypes) and with an Alzheimer's disease diagnosis (7.8% compared to 23.8% and 15.8% for the ‘Typical’ subtypes), suggesting that this subtype reflects a heterogeneous group of individuals with cortical atrophy, some of whom may have Alzheimer’s disease or pre-symptomatic Alzheimer’s disease, but others who may have non-Alzheimer’s disease related changes. A subset of these subjects may reflect a hippocampal-sparing subtype of Alzheimer’s disease^40,42,43^ as they exhibit a similar set of demographic characteristics and atrophy progression pattern (starting in cortical regions with a later hippocampal atrophy) to previously described hippocampal-sparing subtypes. As the ‘Cortical’ subtype had the highest educational level, a slow rate of progression from MCI to Alzheimer’s disease and the best memory function, the ‘Cortical’ group may have a higher cognitive reserve and therefore a higher tolerance to pathology.^48,49^ The literature on the rate of decline of cognition in Alzheimer’s disease subtypes is inconclusive,^4^ with some studies suggesting that typical Alzheimer’s disease has the fastest rates of decline^39,40^ and others suggesting that hippocampal-sparing Alzheimer’s disease has the fastest rates of decline^6,50-52^ Our study suggests that typical Alzheimer’s disease has the fastest rate of decline, however the ‘Cortical’ subtype combines individuals across a range of disease stages and includes a high proportion of individuals who are amyloid negative. It is possible that the rate of decline is faster in individuals who are amyloid positive.

The ‘Cognitive’ subtype was characterized by the lowest educational level, a high prevalence of APOE4 positivity, a high proportion of individuals with an Alzheimer’s disease diagnosis and the fastest MCI to Alzheimer’s disease rate of progression. This group had the worst executive and memory function and a low burden of WMHV. This group may correspond to previously described ‘no atrophy’ or ‘minimal atrophy’ subtypes,^39,40,53^ which are characterized by subjects showing no or minimal atrophy, intermediate age at onset, high amyloid deposition and low tau pathology. However, in some studies, the minimal atrophy subtype has been related with a less aggressive disease progression,^39^ which conflicts with our finding of a fast rate of MCI to Alzheimer’s disease progression in the ‘Cognitive’ subtype. An alternative possibility is that previously described minimal atrophy subtypes are consistent with early stages of the SuStaIn subtypes as SuStaIn accounts for disease severity. The ‘Cognitive’ subtype may represent a subset of individuals previously assigned to ‘no atrophy’ or ‘minimal atrophy’ subtypes.

Finally, the ‘Subcortical’ subtype comprised only a small number of subjects, who were mostly male, had a high educational level, a low percentage of amyloid and APOE4 positivity and the best executive function of the subtypes. They also presented a high burden of WMHV, which might indicate to a group with a strong influence of vascular pathology. One possibility is that the ‘Subcortical’ subtype might represent previously described limbic predominant subtypes of Alzheimer’s disease^6,54^ due to the similarities in disease progression pattern (high atrophy in subcortical areas, such as the hippocampus, and a slow disease progression). Some studies investigating the role of white matter hyperintensities in Alzheimer’s disease subtypes have found that the limbic predominant subtype presents with the highest vascular burden,^4,38,42^ aligning with our finding of higher WMHV. However, we also note that the ‘Subcortical’ subtype was the only group not replicated when running SuStaIn in amyloid-positive individuals only. This suggests that either this subtype has a very low prevalence or that it is not a true Alzheimer’s disease subtype.

The present study has a number of limitations that warrant consideration in future work. SuStaIn makes a series of assumptions to enable modelling of cross-sectional data. SuStaIn assumes an arbitrary timescale to create pseudo-longitudinal sequences. A temporal version of SuStaIn that leverages longitudinal data to learn timescales of progression could be a potential way to address this limitation in the future.^55^ SuStaIn assumes that individuals belong to a single (subtype) progression pattern with a distinct mode. It is possible that there is a continuous spectrum of disease progression patterns, rather than a set of distinct trajectories. SuStaIn requires that a set of *Z*-scores are specified for each marker; we chose the *Z*-scores to be reflective of the range of values for each marker. However, specific combinations of *Z*-scores across markers may be under-represented in the dataset, manifesting as uncertainty in the positional variance diagrams. SuStaIn currently handles relatively small sets of features only. Leveraging a more comprehensive dataset could offer a richer picture, particularly in combination with feature selection strategies to determine the most informative biomarkers or features. Such an approach could balance computational efficiency and information richness. Our study only considers data from a research setting, further work will be required to verify the applicability of our findings in broader cohorts, such as population cohorts and clinical trials. Moreover, as the ADNI cohort is enriched for individuals with Alzheimer’s disease, it is possible that earlier stages of the disease are under-represented, which could also be investigated in population cohorts.

Our contributions to understanding the phenotypic and temporal heterogeneity of Alzheimer’s disease are threefold. First, we updated the SuStaIn algorithm to handle missing data, including validation experiments, making SuStaIn applicable to multimodal data for the first time. Second, we applied ‘missing data SuStaIn’ to the ADNI dataset to both map out the temporal and phenotypic heterogeneity of Alzheimer’s disease across molecular, imaging and cognitive biomarkers, and to characterize the value of each data modality for performing patient subtyping. Third, we showed potential clinical utility whereby SuStaIn subtypes display considerable variability in their conversion of MCI to Alzheimer’s disease. Missing-data SuStaIn has broad applications across a wider range of neurodegenerative diseases and in other progressive conditions.

## Supplementary Material

fcae219_Supplementary_Data

## Data Availability

The data that support the findings of this study are openly available at https://adni.loni.usc.edu/data-samples/access-data/. Source code for missing-data-enabled SuStaIn is available at https://github.com/ucl-pond/pySuStaIn.
