# Supplementary material for: Multimodal subtypes identified in Alzheimer’s Disease Neuroimaging Initiative participants by missing-data-enabled subtype and stage inference
Source: Brain Commun. 2024 Jun 25;6(4):fcae219. doi: 10.1093/braincomms/fcae219 (PMC11259979; doi:10.1093/braincomms/fcae219)
Supplement: fcae219_Supplementary_Data [file fcae219_supplementary_data.zip › Original_submission_manuscript.pdf .pdf]

OXFORD UNIVERSITY PRESS | Brain Communications

Multi-modal subtypes identified in ADNI by missing-data-enabled Subtype and Stage Inference

Journal:	*Brain Communications*
Manuscript ID	BRAINCOM-2023-172
Manuscript Type:	Original Article
Date Submitted by the Author:	02-May-2023
Complete List of Authors:	Estarellas, Mar; University College London, Centre for Medical Image Computing (CMIC), Department of Computer Science<br>Oxtoby, Neil; University College London, Centre for Medical Image Computing (CMIC), Department of Computer Science<br>Schott, Jonathan; University College London Institute of Neurology, Dementia Research Centre<br>Alexander, Daniel; University College London, Centre for Medical Image Computing (CMIC), Department of Computer Science<br>Young, Alexandra; King's College London, Department of Neuroimaging, Institute of Pyschiatry, Pyschology and Neuroscience; University College London, Centre for Medical Image Computing (CMIC), Department of Computer Science
Keywords:	Alzheimer's disease, subtyping, heterogeneity, missing data, multimodal

SCHOLARONE™
Manuscripts

Multi-modal subtypes identified in ADNI by missing-data-enabled Subtype and Stage Inference

Mar Estarellas[1], Neil P Oxtoby[1], Jonathan M Schott[2], Daniel C Alexander[1], Alexandra L Young[3], for the Alzheimer's Disease Neuroimaging Initiative*

*Data used in preparation of this article were obtained from the Alzheimer's Disease Neuroimaging Initiative (ADNI) database (adni.loni.usc.edu). As such, the investigators within the ADNI contributed to the design and implementation of ADNI and/or provided data but did not participate in analysis or writing of this report. A complete listing of ADNI investigators can be found at:
http://adni.loni.usc.edu/wp-content/uploads/how_to_apply/ADNI_Acknowledgement_List.pdf

Abstract

Alzheimer's disease (AD) is a highly heterogeneous disease in which different biomarkers are dynamic over different windows of the decades-long pathophysiological processes, and potentially have distinct involvement in different subgroups. Subtype and Stage Inference (SuStaIn) is an unsupervised learning algorithm that disentangles the phenotypic heterogeneity and temporal progression of disease biomarkers, providing disease insight and quantitative estimates of individual subtype and stage. However, a key limitation of SuStaIn is that it requires a complete set of biomarkers for each subject, reducing the number of data points available for model fitting and limiting applications of SuStaIn to modalities that are widely collected, e.g. volumetric biomarkers derived from structural MRI. In this study, we adapted the SuStaIn algorithm to handle missing data, enabling the application of SuStaIn to multimodal data (magnetic resonance imaging, positron emission tomography, cerebrospinal fluid and cognitive tests) from 789 participants in the Alzheimer's Disease Neuroimaging

For Review Only

Initiative (ADNI). Missing-data SuStaIn identified five subtypes having distinct progression patterns, which we describe by the earliest unique abnormality as 'Typical AD with Early Tau', 'Typical AD with Late Tau', 'Cortical', 'Cognitive' and 'Subcortical'. These new multi-modal subtypes were differentially associated with age, years of education, APOE4 status, white matter hyperintensity burden, and the rate of conversion from mild cognitive impairment to AD, with the 'Cognitive' subtype showing the fastest clinical progression, and the 'Subcortical' subtype the slowest. Overall, we demonstrate that missing-data SuStaIn reveals a finer landscape of AD subtypes, each of which are associated with different risk factors. Missing-data SuStaIn has broad utility, enabling the prediction of progression in a much wider set of individuals, rather than being restricted to those with complete data.

Keywords: Alzheimer's disease, subtyping, heterogeneity, missing data, multimodal

For Review Only

Author affiliations:

1 Centre for Medical Image Computing, Department of Computer Science, University College London, London, United Kingdom

2 Dementia Research Centre, UCL Queen Square Institute of Neurology, London, UK

3 Department of Neuroimaging, Institute of Psychiatry, Psychology and Neuroscience, King's College London, London, United Kingdom

Correspondence to: Mar Estarellas, Centre for Medical Image Computing, Department of Computer Science, University College London, London, United Kingdom

rmapest@ucl.ac.uk

Running title: Multimodal subtypes in ADNI

Keywords Alzheimer's disease; subtyping; heterogeneity; missing data; multimodal.

Introduction

Alzheimer's disease (AD) is clinically and pathologically heterogeneous. This heterogeneity potentially has important implications for clinical trials as heterogeneity may mask the benefit of a treatment that is effective in a particular subgroup[1],[2]. This motivates recent efforts to unravel the heterogeneous temporal progression patterns of AD using disease biomarkers and their relationship with clinical presentation, genetics, risk factors and multi-morbidity [1–8]; this in turn potentially enables stratification for more targeted clinical trials and assisting clinicians in patient management [9].

Established AD biomarkers include cerebrospinal fluid (CSF) [10] and positron emission tomography (PET) imaging of amyloid and tau accumulation [11,12]; magnetic resonance imaging (MRI) of regional brain atrophy [13]; and cognitive test scores [14]. As each of these modalities provide different information and are likely dynamic at different stages [15], integrating information from a range of different disease biomarkers is key to building a complete picture of AD [16,17].

Data-driven computational models enable the construction of a quantitative picture of the phenotypic and temporal heterogeneity of AD. However, most studies focus either on temporal (staging) [15,18–20] or phenotype (subtyping) differences [4,21], which risks conflating disease subtypes with disease stages. Subtype and Stage Inference (SuStaIn) [3] is an unsupervised machine learning algorithm that uses clustering and data-driven disease progression modelling techniques to identify subgroups of individuals with distinct disease trajectories. This has the advantage of simultaneously disentangling phenotypic heterogeneity (the presence of different disease subtypes) and temporal progression

For Review Only

(different stages of the disease). The algorithm requires only cross-sectional data but can use longitudinal data if available. To date SuStaIn applications have mostly exploited single-modality data-sets, such as MRI or PET [3,22], or occasionally two data-types such as Tau CSF and PET together [23]. SuStaIn models built on more diverse sets of markers would provide a more complete picture of the disease time course and landscape of subtypes [16,17].

One of the major challenges when applying SuStaIn to multi-modal data is "missing data", as many study subjects miss one or more modality due to refusal or funding constraints [24] . Whilst there are several different versions of SuStaIn that enable the use of a range of disease progression models for different data types [25–28], none are well suited to modelling multi-modal biomarker data. The most widely used version of the SuStaIn algorithm is z-score SuStaIn [3]. Z-score SuStaIn is the most appropriate version of SuStaIn to use for most modalities as it models the continuous evolution of biomarkers from one z-score to another, capturing the gradual change of biomarkers with disease progression. However, z-score SuStaIn currently requires complete data for all subjects. Alternative versions of SuStaIn, such as event-based SuStaIn [25,26,28] and ordinal SuStaIn [27], more readily handle missing data but are limited in the types of progression and data they can model; event-based SuStaIn can only model discrete transitions from a normal to an abnormal level, whilst ordinal SuStaIn can only model discrete scored data such as visual or neuropathological ratings data.

Here we propose an adaptation of z-score SuStaIn to allow for missing data, which we refer to as missing-data SuStaIn. We first validate our implementation of missing-data

For Review Only

SuStaIn using a synthetic dataset. We use missing-data SuStaIn to identify subtypes with distinct progression patterns using multimodal data including PET, CSF, MRI and cognitive scores from the ADNI dataset. We then evaluate the associations between each subtype and demographics, cognitive scores, white matter hyperintensity volumes and age at death. Finally, we test whether the multi-modal subtypes and stages identified by missing-data SuStaIn provide clinical utility for predicting conversion from mild cognitive impairment (MCI) to AD.

For Review Only

Materials and methods

Subtype and Stage Inference (SuStaIn)

To estimate subgroup progression patterns, SuStaIn [29] simultaneously clusters individuals into groups (subtypes) and uses disease progression modelling to reconstruct a disease progression pattern (set of stages) for each subgroup. Z-score SuStaIn models disease progression using a piecewise linear z-score model. The piecewise linear z-score model describes disease progression as a series of stages, where each stage corresponds to a new biomarker reaching a new z-score. The z-score SuStaIn algorithm then consists of simultaneously optimising subtype membership, and stage progression, building on the well-established methods developed for the event-based model [25,26] and incorporating ideas from clustering. SuStaIn evaluates each subject's likelihood of belonging to each subtype and stage, outputting the probability each subject belongs to each subtype and stage together with their maximum likelihood subtype and stage assignment.

The missing data adaptation we propose requires modification of the data likelihood. The data likelihood $P(X|M)$ for the z-score model in SuStaIn (derived in detail in [3]) can be written as:

$$P(X|M) = \prod_{j=1}^{J}\left(\sum_{c=1}^{C} f_c \sum_{k=0}^{N}\left[\int_{t=\frac{k}{N+1}}^{t=\frac{k+1}{N+1}}\left(P(t)\prod_{i=1}^{I}P(x_{ij}|t)\right)\partial t\right]\right) \quad (1)$$

where x_{ij} is the measurement of biomarker i in subject j, $j = 1 \ldots J$. C is the number of clusters (subtypes), f is the proportion of subjects assigned to a particular cluster (subtype), and M is the overall SuStaIn model. $P(x_{ij}|t)$ is usually modelled as a normal distribution around the piecewise linear trajectory $g_i(t)$ with variance σ_i estimated from a control population:

$$P(x_{ij}|t) = NormPDF\,(x_{ij}, g_i(t), \sigma_i). \quad (2)$$

The piecewise linear trajectory is described by an ordering of a set of N z-score events $E_{\{iz\}}$, where each event corresponds to the linear increase of a biomarker $i = 1 \ldots I$ to a z-score $z_{\{i1\}} \ldots z_{\{iR_i\}}$. Each biomarker trajectory is parameterised to start at $z = 0$ and end at $z = z^{max}$.

Missing Data Adaptation

The strategies for dealing with missing data are described below. Our proposed approach, which we label *MD1*, was benchmarked against alternative pre-processing approaches that handle missing data by deleting or inferring missing values of x_{ij}, *MD2-4*.

For Review Only

MD1: Uniform

We handle missing data in SuStaIn by modelling $P(x_{ij}|\mathrm{t})$, in the absence of x_{ij}, and evaluating equation (1) using

$$P(x_{ij} \mid t) = \begin{cases} \mathrm{NormPDF}\left(x_{ij}, g_i(t), \sigma_i\right), & x_{ij} \text{ is present} \\ P'\left(x_{ij} \mid t\right), & x_{ij} \text{ is missing} \end{cases} \quad (3)$$

When x_{ij} is missing, we propose modelling the distribution of x_{ij} as uniform over the range of the z-scores for that biomarker, so that $P'(x_{ij} \mid t) = \frac{1}{z_i^{max}}$. This strategy means that missing biomarker entries have no effect on the estimation of the subtypes and stages within SuStaIn, whilst still enabling the available biomarker entries for each participant to contribute to the subtype and stage estimation.

This adaption can be used in both the training phase – estimating the subtypes and trajectories – and the test phase – assigning subtypes and stages to individuals. The Python implementation of the SuStaIn algorithm (pySuStaIn) was adapted to enable subtype progression patterns to be estimated and to allow subtyping and staging of individuals from incomplete data. The implementation can be found in the pySuStaIn package [29] and is available on github (https://github.com/ucl-pond/pySuStaIn).

For Review Only

MD2: Deletion

All subjects with one or more missing biomarkers are excluded from the dataset, as in previous applications of z-score SuStaIn.

MD3: Imputing the mean

This approach imputes the missing datapoint as the mean of that biomarker over all subjects with non-missing values, i.e. $x_{ij} = \frac{1}{N}\sum_{i=1}^{N} x_{ij}$. It is computationally fast but has clear disadvantages such as reducing variance in the dataset.

MD4: Imputation using K-nearest neighbours (KNN)

This approach imputes the missing datapoint as the mean value of that biomarker over a set of K similar subjects, who are identified using KNN. KNN is a supervised learning algorithm that finds the K nearest subjects using a distance metric [30]. As the modalities we wish to impute vary considerably in magnitude, we computed the distance between subjects using two different subject level feature vectors: 1) the mean, range and standard deviation of an individual's biomarker values 2) an individual's z-scored biomarker data. Then the following iterative procedure based on a simple KNN algorithm was computed, for each missing biomarker value for each subject:

1. In the feature vector extracted (composed of either the mean, standard deviation and range for each subject, or the z-scored data), find the K subjects that have the most similar features, using Euclidean distance between the two vectors being compared.

2. Go to the original data matrix and find the values of the missing biomarker from those K most similar subjects.

3. Compute the average of such value and replace the missing biomarker value.

For Review Only

Datasets

Synthetic Dataset

A synthetic dataset of 500 subjects and 10 biomarkers was generated to test different approaches to handling missing data. The data was simulated as in the original SuStaIn paper [3]. The default number of clusters was set to C = 3, and no biomarker covariance, setting Σ to the identity matrix. SuStaIn stages were simulated using a uniform distribution and SuStaIn subtypes, using the following fraction: $f_c = \frac{2 + (C - c)}{2C + \sum_{C=1}^{C}(C - c)}$. Thus, the fraction of subjects belonging to each cluster are $f_1 = \frac{4}{9}$, $f_2 = \frac{3}{9}$, $f_3 = \frac{2}{9}$. The progression pattern for each cluster is simulated as a linear z-score model with a random monotonic ordering of the z-score events, fixing z_i = (1,2,3) and z_{imax}= 5 for all biomarkers *i*. From this dataset, 150 random values (3% of the 5000 datapoints) were deleted to mimic missing values. The resulting dataset was used to compare different approaches to handling missing data. We benchmarked the performance of each approach to handling missing data both against each other and against performance on the full dataset

ADNI Dataset

Data used in the preparation of this article were obtained from the Alzheimer's Disease Neuroimaging Initiative (ADNI) database (adni.loni.usc.edu). The ADNI was launched in 2003 as a public-private partnership, led by Principal Investigator Michael W. Weiner, MD. The primary goal of ADNI has been to test whether serial magnetic resonance imaging (MRI), positron emission tomography (PET), other biological markers, and clinical and neuropsychological assessment can be combined to measure the progression of mild cognitive impairment (MCI) and early Alzheimer's disease (AD). For up -to-date

For Review Only

information, see www.adni-info.org. Written consent was obtained from all participants, and the study was approved by the Institutional Review Board at each participating institution.

The inclusion criteria for our study were the availability of cross-sectional Freesurfer volumes derived from a 3T MRI scan at baseline and that these volumes passed overall quality control. Nine follow-up visits (up to month 42 according to ADNI's records) were also used for experiments in this work (see Supplementary Analysis – Longitudinal Consistency). The resulting dataset consisted of 789 subjects (182 CN, 86 significant memory concern (SMC), 241 early MCI, 163 late MCI and 117 AD). For this study, ADNI diagnosis was divided into three categories: controls (CN), AD and MCI. The group of controls includes subjects diagnosed as healthy and those with subjective memory complains. Late and early MCI were grouped together as MCI. The downloaded Freesurfer values were used to compute the volume of 6 cortical regions (Frontal lobe, Temporal lobe, Occipital lobe, Parietal lobe, Cingulate, Insula) and 4 subcortical regions (hippocampus, amygdala, thalamus, and basal ganglia formed by the accumbens, pallidum, putamen and caudate).

Several additional biomarkers were downloaded comprising measurements from CSF, PET and cognitive tests. CSF measurements included Aβ, tau and p-tau, for which 109 subjects had missing data. PET measures of florbetapir (18F-AV45)-PET averaged of angular, temporal, and posterior cingulate; and fluorodeoxyglucose (FDG) mean of whole cerebellum, were downloaded from the ADNIMERGE spreadsheet. 86 subjects had missing FDG-PET data and 92 subjects missing AV45 data. Three cognitive test scores were selected from the ADNIMERGE spreadsheet/table: the Alzheimer's Disease Assessment Scale-Cognitive Subscale (ADAS-Cog 13) [31], Rey Auditory Verbal Learning Test – RAVLT (RAVLT immediate sum of 5 trials) [32] and the time to complete the Trail Making Test

For Review Only

(TRABSCOR) [33], for which only 33 subjects had missing entries. Finally, demographic data (age, sex, education), APOE genotype and total intracranial volume was also downloaded from ADNIMERGE table for covariate correction. Overall, baseline measurements had a total of 3.8% of the data missing, of which 60.8% was CSF data, 33% corresponded to missing PET and 6.1%, cognitive data.

Experiments

Evaluation of missing data modelling using synthetic data

The synthetic data set enables evaluation of subtype and stage estimates against known ground truth values. To select the most effective strategy to deal with missing data, we evaluate the results obtained with the different methods. The event sequence similarity between two subtype progression patterns was computed using the Kendall tau distance between the sequence of the biomarkers obtained with each approach versus the ground truth sequence. Subtyping accuracy was reported as the percentage of subjects who were subtyped correctly (the estimated subtype coincided with the ground-truth subtype). Finally, the accuracy of patient staging is reported per subtype as the mean error ± standard deviation (across individuals) between the estimated stage and the ground-truth stage.

For Review Only

SuStaIn modelling in ADNI

All biomarkers were corrected for age, sex, education and, in the case of imaging markers, total intracranial volume. The correction was performed by estimating a linear regression model in a control population of 89 amyloid negative CN participants (CSF Aβ1-42 < 192pg/ml [34]), and then propagating this model to all 789 participants for the subset of associations that were significant. Baseline data of the corrected biomarkers were then converted into *z* scores relative to the control population for use as input to SuStaIn. Z-scores of up to 5 were used in this study (1, 2, 3 and 5). The minimum biomarker z score was set to

zero for all biomarkers, while the maximum was set to the rounded 95th percentile computed for each biomarker. Biomarkers that decrease with disease progression were multiplied by −1 to give positive *z* scores that increase with disease progression. Longitudinal data used in the Supplementary Analysis of Longitudinal Consistency were z-score transformed and covariate corrected using the same method performed in the baseline data.

SuStaIn was applied to three different data subsets: 1) All 789 subjects; 2) β-amyloid positive individuals, n= 406, (AB+) ; and 3) β-Amyloid negative individuals, n=275 (AB-) based on CSF cut-offs according to [34(p20)]. The optimal number of subtypes was chosen by comparing the distribution of model likelihoods. The individual subject subtype assignments under each model were compared using a Sankey diagram, a typical diagram used to visualize the directed flow between nodes or different sets of values.

Differences between subtypes in age, years of education, sex, ADNI diagnosis and number of APOE4 alleles were studied using a t-test for continuous variables or Chi square test for discrete variables. Memory and executive function were also compared between subtypes, computed using the the Memory and Executive Function Cognitive battery from the ADNI-composite scores [35,36], as were white matter hyperintensity values (WMHV) [37].

Predictive Utility of SuStaIn

Cox proportional hazard models were used to assess the predictive utility of SuStaIn for predicting conversion from MCI to AD. Age, gender, APOE4 status and SuStaIn stage were used as covariates. The risk of MCI-to-AD conversion was assessed using hazard ratios for

For Review Only

SuStaIn subtype and stage. A ratio of 1 means no modification of the risk of MCI to AD conversion, a ratio>1 means increase of risk and ratio <1, decrease of risk.

To further validate Missing data SuStaIn and its contribution to clinical practice, the cox proportional hazards models were fitted to three groups: 1) All subjects, 2) subjects with complete data only, 3) subjects with missing data only. Results were compared using the 95% confidence interval (CI) of resulting hazard ratios for subtype.

Supplementary analysis

Benchmarking modalities

We assessed the importance of different modalities for subtyping and staging of individuals by calculating the consistency of subtypes and stages when artificially treating a modality as missing vs. using their full data as a proxy ground truth.

Longitudinal Consistency

The consistency of subtyping and staging in longitudinal data was also compared for different percentages of missing data. Follow-up subtype assignments were deemed longitudinally consistent if: a participant progressed to a subtype from “normal-appearing” (i.e. no evidence of biomarker abnormality and therefore not subtypable); or if they were assigned to the same subtype at follow-up. Follow-up stages were deemed longitudinally consistent if a participant remained at the same stage or progressed to a later stage at follow-up. The 95% confidence interval (CI) was computed by determining the set of subtypes and stages that fell within a cumulative probability of 0.95.

For Review Only

Data availability

The data that support the findings of this study are openly available at https://adni.loni.usc.edu/data-samples/access-data/. Source code for Missing-data-enabled SuStaIn is available at https://github.com/ucl-pond/pySuStaIn.

Results

Validation of Missing data SuStaIn using a synthetic dataset

Table 1 shows that the uniform model of $P(x_{ij}|t)$ optimizes performance in recovering subtype trajectories in synthetic data experiments. The uniform distribution approach consistently resulted in the highest correlation between the estimated and ground truth progression patterns measured by Kendall Tau rank correlation coefficient (0.93, 0.97 and 0.89 for clusters 1, 2 and 3 respectively). This approach also produced the most accurate subtyping and staging, where 86% of the subjects were classified correctly compared to the ground truth subtypes; and there was a mean difference of 1.54 ($\pm$ 1.30) stages across individuals. The uniform distribution approach, which we term "missing data SuStaIn", was consequently used for all further analyses.

Multi-modal subtypes identified by SuStaIn

Figure 1 shows the temporal progression of the five different subtypes identified by missing data SuStaIn when applied to all 789 subjects from ADNI (Aβ + and Aβ -). We labelled these five different subtypes as: 'Typical AD Early Tau', 'Typical AD Late Tau', 'Cortical', 'Cognitive' and 'Subcortical'.

235 subjects were assigned to the 'Typical AD Early Tau' subtype. This subtype was characterized by an early CSF tau, p-tau and PET AV45 change, followed by CSF Aβ and cognitive decline. MRI volume changes were seen to appear late for this group of subjects.

For Review Only

The ‘Typical AD Late Tau’ subtype (203 subjects) had a very similar progression pattern to the ‘Typical AD Early Tau’ subtype; however, in this case, the CSF markers of tau and p-tau appear later, after MRI markers change. The ‘Cortical’ and ’Cognitive’ subtypes (n = 141 and 58, respectively) were characterized by early cortical atrophy and early cognitive decline, respectively. Only 37 subjects were assigned to the ‘Subcortical’ subtype, which shows an uncertain progression pattern with early subcortical atrophy. This may suggest the subcortical subtype comprises a collection of individuals with heterogeneous patterns that have common atrophy in subcortical regions such as the amygdala and hippocampus.

For Review Only

Missing-data SuStaIn was also applied to only amyloid positive (Aβ+) (Supplementary Fig.1) and amyloid negative (Aβ-) (Supplementary Fig.2) ADNI subjects. Figure 2A shows a Sankey diagram comparing the subtype assignments of Aβ+ individuals based on subtype progression patterns (i) learnt in the whole population and (ii) learnt in only Aβ+ individuals. Similarly, Figure 2B shows a Sankey diagram comparing the subtype assignments of Aβ- individuals based on subtype progression patterns (i) learnt in the whole population and (ii) learnt in only Aβ- individuals. Application of SuStaIn to Aβ+ subjects recovered four of the five subtypes: ‘Typical AD Early Tau’, ‘Typical AD Late Tau’, ‘Cortical and ‘Cognitive’, whereas application of SuStaIn to Aβ- subjects recovered three of the five subtypes: ‘Cortical and ‘Cognitive’ and ‘Subcortical’. Thus, ‘Typical AD Early Tau’, ‘Typical AD Late Tau’ subtypes were unique to the Aβ+ group, whereas the ‘Subcortical’ subtype appeared only in Aβ- subjects.

Results in the following sections were computed using the SuStaIn model learnt from all 789 subjects, unless stated.

Relationship of subtypes with demographics and risk factors

Figure 3 and Table 2 show the demographic variables of education, age, gender, APOE4 status, amyloid positivity and ADNI diagnosis for each SuStaIn-assigned subtypes. Subjects assigned to the 'Cognitive' subtype had a mean of 1.1 fewer years of education than all other subtypes, this difference being significant when compared to the 'Normal Appearing' (p-value = 0.005 cognitive vs. normal appearing t-test), 'Typical Late Tau' (p-value = 0.01 cognitive vs. typical late tau t-test) and 'Cortical' (p-value = 0.02 cognitive vs. cortical t-test) subtypes. Individuals with the 'Cortical' subtype were on average 3.4 years younger than any other subtype (p-values < 0.001 vs. typical early tau, typical late tau, cognitive and subcortical respectively t-test); and those assigned to the 'Subcortical' subtype were the oldest (mean 1.93 years; p-values < 0.001 vs. typical early tau, typical late tau, cognitive and cortical respectively t-test). Significantly more females were found in the 'Normal appearing' subtype (4.9% more females; p-values<0.05 vs. typical late tau, cognitive, cortical and subcortical respectively chi-square test) and the 'Typical Early Tau' subtype (5.5% more females; p-values < 0.05 vs. typical late tau, cognitive, cortical and subcortical respectively chi-square test). The proportion of subjects who were APOE4 positive (one or more APOE4 alleles) and with ADNI diagnosis of AD was significantly larger in the two Typical AD subtypes and the Cognitive subtype (p-values < 0.02 vs. cortical and subcortical respectively chi-square test).

A significantly higher proportion of subjects were amyloid positive in the 'Typical Late Tau' and 'Typical Early Tau' subtypes, 82% and 85% of the data, respectively (p-values < 0.001 vs. cognitive, cortical, and subcortical t-test respectively). The 'Subcortical' subtype had the lowest proportion of amyloid positive subjects, with only 27% (p-values < 0.001 vs. typical early tau, typical late tau, cognitive and cortical respectively t-test). The two Typical AD

For Review Only

subtypes and the Cognitive subtype had the highest proportion of AD diagnosed subjects, with 24 and 22 percent of AD subjects in each subtype. The Cortical and Subcortical subtypes had the lowest proportion of AD diagnosed subjects; 8% and 11% (p-values < 0.0001 vs. typical early tau, typical late tau, cognitive and cortical respectively t-test). MCI diagnosis was more prevalent in the Subcortical subtype than all other subtypes, with 70% of the data belonging to this diagnosis category (p-values < 0.0001 vs. typical early tau, typical late tau, cognitive and cortical respectively t-test).

Figure 4 shows the difference in memory and executive function between subtypes. Cognitive subtype subjects show the worst performance in both cognitive batteries, with -0.1 and -0.6 for memory and executive function, respectively. These low scores are found to be significantly different from all other subtypes (Memory function and executive function cognitive battery p-value <0.05 cognitive subtype vs typical early tau, typical late tau, cortical and subcortical t-test), except when comparing memory function with the 'typical Early Tau' subtype.

For Review Only

Prediction of conversion

Supplementary Fig.3 shows that the different SuStaIn subtypes have distinct risks of MCI to AD conversion. By fitting a Cox Proportional Hazard model, we found significant effects of subtype and stage in the risk of progressing from MCI to AD, based on ADNI diagnosis labels. Of the SuStaIn subtypes, the 'Cognitive' subtype was associated with the highest rate of progression (HR=12. 82 (4.20 – 39.1 95% CI) p-value < 0.001), while the 'Subcortical' subtype was associated with the lowest, although non-significant (HR=1.75 (0.40 – 7.7) p-value =0.45), 'Typical AD Early Tau' and 'Typical AD Late Tau' have very similar

conversion rate (HR= 6.11 (2.26 – 16.5) p-value<0.001; and HR = 7.58 (2.83 - 20.3) p-value<0.001), showing a faster conversion than the 'Cortical' and 'Subcortical subtype, however slower than 'Cognitive'. SuStaIn stage was also found to be significant (HR= 1.08 (1.06 – 1.1) p-value < 0.001) when computing MCI to AD conversion in ADNI data, with each increase in stage corresponding to an 8% increase in the hazard ratio

Evaluating the effect of missing data in the prediction of MCI to AD conversion

Figure 5 shows the Kaplan Meier curves for MCI to AD progression, fit to each of three different data sets: all subjects (number of MCI subjects at baseline, n=689), only subjects with complete data at baseline (n=533), only subjects with missing data at baseline (n=156). In all datasets 'Cognitive' showed the fastest progression, followed by 'Typical Late Tau' and 'Typical Early Tau', which have a very similar rate of progression; then 'Cortical', and finally 'Subcortical' showed the slowest MCI to AD progression. The overall hazard ratios of SuStaIn subtype for each dataset were found to be 1.10 (0.96-1.26 CI) in all MCI subjects, 1.06 (0.90-1.35 CI) in the subset of MCI subjects with complete data and 1.36 (1.00-1.81) in MCI subjects with missing data. The results show no statistical difference between models, suggesting that missing-data SuStaIn produces accurate enough subtype and stage assignments to predict MCI to AD conversion when data is missing.

For Review Only

Evaluating the effect of multimodal data versus MRI only in the prediction of MCI to AD conversion

By performing likelihood ratio tests comparing the prediction of MCI to AD conversion using only MRI or multimodal data to fit SuStaIn, it was found that using multimodal data SuStaIn provides a significantly better fit for the Cox Proportional Hazards models than using

only MRI data ($p = 2.20 \times 10{-}6$). This shows that using multimodal provides additional information for predicting the risk of conversion from MCI to AD.

Supplementary analyses

Benchmarking modalities.

Figure S4A suggests that the most important modalities for subtyping are PET/CSF (treated in combination). Figure S4B shows that MRI is the modality that most strongly affects the staging similarity when different biomarkers are removed, leading to a bigger spread of the staging results. Table S1 demonstrates that whilst the uncertainty in the subtypes and stages increases when a modality is missing, the confidence interval is representative of the true subtype and stage.

Longitudinal consistency

Figure S5 shows that longitudinal subtype assignment is most consistent for individuals with high confidence in subtype assignment at every visit, suggesting that SuStaIn provides meaningful estimates of the overall confidence in the subtype assignments. Among subjects that were assigned to a subtype with a probability of 0.9 or higher, there was baseline to follow-up subtype consistency of 86.3%. Whilst longitudinal subtype consistency decreased with subtype probability, the 95% confidence intervals of the subtype assignments showed a strong correspondence between baseline and follow-up, regardless of the subtype probability; the mean percentage over all visits of subjects in which the confidence intervals overlapped

For Review Only

between baseline and follow-up was 100% (Table S2). This demonstrates the utility of the confidence intervals of the SuStaIn subtypes.

Discussion

In this work we developed and applied Missing data SuStaIn to model the temporal and phenotypic heterogeneity of an Alzheimer's disease (AD) enriched cohort, ADNI, using multimodal data. We evaluated alternative methods of handling missing data in SuStaIn, finding the most effective way of treating missing data using a synthetic dataset, which we termed 'missing-data SuStaIn'. We demonstrated that missing-data SuStaIn outperforms alternative methods that impute missing data. Our proposed adaptation enabled SuStaIn to be applied to a multi-modal set of biomarkers, uncovering five subtypes: 'Typical AD Early Tau, 'Typical AD Late Tau', 'Cortical', 'Cognitive', and 'Subcortical', in order of prevalence. The 'Typical AD Early Tau, 'Typical AD Late Tau', 'Cortical' and 'Cognitive' subtypes were replicated when running missing-data SuStaIn in amyloid-positive individuals only, whilst the 'Subcortical', 'Cortical' and 'Cognitive' subtypes were also found in amyloid-negative individuals. We found that subjects assigned to each subtype had significantly different demographic profiles and rates of MCI to AD conversion, which could potentially inform clinical practice.

'Typical' subtypes of Alzheimer's disease have been repeatedly described in atrophy-only studies [38–40]. By including CSF markers in SuStaIn, we found that the typical subtype subdivided into a typical subtype with early tau abnormalities 'Typical Early Tau' and a typical subtype showing a later tau deposition 'Typical Late Tau'. The 'Typical Early Tau'

For Review Only

subtype appears to correspond with an archetypical pathological progression of AD, where tau starts accumulating in the brain first but doesn't reach an abnormality threshold until Aβ deposition does [15]. Similar subtypes have previously been found using both SuStaIn [3,41] and other data-driven methods [38,40,42,43]. The 'Typical Early Tau' subtype contains a large number of individuals with an AD diagnosis, and a high proportion of amyloid positive and APOE4 positive individuals, reinforcing the correspondence of this subtype to a typical AD progression pattern.

The 'Typical Late Tau' subtype appears to largely reflect a typical pattern of AD except tau deposition happens much later in the disease progression. A similar 'late tau' subtype was found by another study that applied SuStaIn to CSF and PET [23]. Several other works have previously studied the differences between subjects presenting a typical AD progression with high or low levels of Tau. These studies found that those with higher concentration of tau were more likely to exhibit an executive phenotype of the disease, rather than amnestic [44–46]. In our study, 'Typical Early Tau' and 'Typical Late Tau' also showed significant differences in executive function, with subjects assigned to the 'Typical Early Tau' subtype (i.e. a higher concentration of tau earlier in the disease) having lower executive cognitive scores (mean difference = -0.35 p-value = 0.002). We also found significant differences in total white matter hyperintensity, with the 'Typical Late Tau' subtype having significantly higher volumes compared to the 'Normal Appearing' group. A higher predominance of white matter hyperintensities in typical Alzheimer's subtypes has been previously reported [5,38], but here we linked these white matter hyperintensities to the late tau group specifically. 'Typical AD Late Tau' and 'Typical AD Tau First' were shown to have very similar MCI to AD progression rates. This progression rate was faster than most of the

For Review Only

other subtypes, consistent with previous work [42,43,47]. However, in this study we found that the 'cognitive' subtype had the fastest rate of conversion.

Subjects assigned to the 'Cortical' subtype were younger, had the highest educational level and a low percentage of APOE4 positivity. The 'Cortical' subtype subjects also had the best memory function and a slow rate of progression from MCI to AD. These subjects may reflect a hippocampal sparing subtype of AD [40,42,43] as they exhibit a similar set of demographic characteristics and atrophy progression pattern (starting in cortical regions with a later hippocampal atrophy) to previously described hippocampal sparing subtypes. As the 'Cortical' subtype had the highest educational level, a slow rate of progression from MCI to AD and the best memory function, the 'Cortical' group may have a higher cognitive reserve and therefore a higher tolerance to pathology [48,49].

For Review Only

The 'Cognitive' subtype was characterised by the lowest educational level, a high prevalence of APOE4 positivity, a high proportion of individuals with an AD diagnosis, and the fastest MCI to AD rate of progression. This group had the worst executive and memory function and a low burden of WMHV. This group may correspond to previously described 'no atrophy' or 'minimal atrophy' subtypes [39,40,50], which are characterised by subjects showing no or minimal atrophy, intermediate age at onset, high amyloid deposition and low tau pathology. However, in some studies, the minimal atrophy subtype has been related with a less aggressive disease progression [39], which conflicts with our finding of a fast rate of MCI to AD progression in the 'Cognitive' subtype. An alternative possibility is that previously described minimal atrophy subtypes are consistent with early stages of the SuStaIn subtypes

as SuStaIn accounts for disease severity. The 'Cognitive' subtype may represent a subset of individuals previously assigned to 'no atrophy' or 'minimal atrophy' subtypes.

Finally, the 'Subcortical' subtype comprised only a small number of subjects, who were mostly male, had a high educational level, a low percentage of amyloid and APOE4 positivity and the best executive function of the subtypes. They also presented a high burden of WMHV, which might indicate to a group with a strong influence of vascular pathology. One possibility is that the 'Subcortical' subtype might represent previously described limbic predominant subtypes of AD [6,51] due to the similarities in disease progression pattern (high atrophy in subcortical areas, such as the hippocampus, and a slow disease progression). Some studies investigating the role of white matter hyperintensities in AD subtypes have found that the limbic predominant subtype presents with the highest vascular burden [4,38,42], aligning with our finding of higher WMHV. However, we also note that the 'Subcortical' subtype was the only group not replicated when running SuStaIn in amyloid-positive individuals only. This suggests that either this subtype has a very low prevalence or that it is not a true AD subtype.

The present study has a number of limitations that warrant consideration in future work. SuStaIn makes a series of assumptions to enable modelling of cross-sectional data. SuStaIn assumes an arbitrary timescale to create pseudo-longitudinal sequences. A temporal version of SuStaIn that leverages longitudinal data to learn timescales of progression could be a potential way to address this limitation in the future [52]. SuStaIn assumes individuals belong to a single (subtype) progression pattern with a distinct mode. It is possible that there is a continuous spectrum of disease progression patterns, rather than a set of distinct trajectories. Our study only considers data from a research setting, further work will be required to verify

For Review Only

the applicability of our findings in broader cohorts, such as population cohorts and clinical trials. Moreover, as the ADNI cohort is enriched for individuals with Alzheimer's disease, it is possible that earlier stages of the disease are underrepresented, which could also be investigated in population cohorts.

Our contributions to understanding the phenotypic and temporal heterogeneity of AD are threefold. First, we updated the SuStaIn algorithm to handle missing data, including validation experiments, making SuStaIn applicable to multimodal data for the first time. Second, we applied "missing data SuStaIn" to the ADNI dataset to both map out the temporal and phenotypic heterogeneity of AD across molecular, imaging, and cognitive biomarkers, and to characterise the value of each data modality for performing patient subtyping. Third, we showed potential clinical utility whereby SuStaIn subtypes display considerable variability in their conversion of MCI to AD. Missing-data SuStaIn has broad applications across a wider range of neurodegenerative diseases and in other progressive conditions.

Funding

NPO is a UKRI Future Leaders Fellow (MR/S03546X/1). NPO and DCA acknowledge funding from the E-DADS project (EU JPND), EPSRC grant EP/M020533/1, MRC's Health Data Research UK (HDR UK) (HDR-9006; CFC0110) initiative, Wellcome Trust Investigator in Science Award (221915/Z/20/Z), MRC/JPND grant MR/T046422/1 and the National Institute for Health Research University College London Hospitals Biomedical Research Centre (UCL/H BRC). JMS acknowledges support from the UCL/H BRC, the Medical Research Council, Alzheimer's Research UK and the Alzheimer's Association. ALY is supported by an MRC Skills Development Fellowship (MR/T027800/1).

For Review Only

Data collection and sharing for this project was funded by the Alzheimer's Dise ase Neuroimaging Initiative (ADNI) (National Institutes of Health Grant U01 AG024904) and DOD ADNI (Department of Defense award number W81XWH-12-2-0012). ADNI is funded by the National Institute on Aging, the National Institute of Biomedical Imaging and Bioengineering, and through generous contributions from the following: AbbVie, Alzheimer's Association; Alzheimer's Drug Discovery Foundation; Araclon Biotech; BioClinica, Inc.; Biogen; Bristol-Myers Squibb Company; CereSpir, Inc.; Cogstate; Eisai Inc.; Elan Pharmaceuticals, Inc.; Eli Lilly and Company; EuroImmun; F. Hoffmann-La Roche Ltd and its affiliated company Genentech, Inc.; Fujirebio; GE Healthcare; IXICO Ltd.; Janssen Alzheimer Immunotherapy Research & Development, LLC.; Johnson & Johnson Pharmaceutical Research & Development LLC.; Lumosity; Lundbeck; Merck & Co., Inc.; Meso Scale Diagnostics, LLC.; NeuroRx Research; Neurotrack Technologies; Novartis Pharmaceuticals Corporation; Pfizer Inc.; Piramal Imaging; Servier; Takeda Pharmaceutical Company; and Transition Therapeutics. The Canadian Institutes of Health Research is providing funds to support ADNI clinical sites in Canada. Private sector contributions are facilitated by the Foundation for the National Institutes of Health (www.fnih.org). The grantee organization is the Northern California Institute for Research and Education, and the study is coordinated by the Alzheimer's Therapeutic Research Institute at the University of Southern California. ADNI data are disseminated by the Laboratory for Neuro Imaging at the University of Southern California.

For Review Only

Competing interests

The authors report no competing interests.

Supplementary material

Supplementary material is available at *Brain* online

References

1. Ferreira D, Wahlund LO, Westman E. The heterogeneity within Alzheimer's disease. *Aging*. 2018;10(11):3058-3060. doi:10.18632/aging.101638

2. Devi G, Scheltens P. Heterogeneity of Alzheimer's disease: consequence for drug trials? *Alzheimer's Research & Therapy*. 2018;10(1):122. doi:10.1186/s13195-018-0455-y

3. The Genetic FTD Initiative (GENFI), The Alzheimer's Disease Neuroimaging Initiative (ADNI), Young AL, et al. Uncovering the heterogeneity and temporal complexity of neurodegenerative diseases with Subtype and Stage Inference. *Nature Communications*. 2018;9(1):4273. doi:10.1038/s41467-018-05892-0

4. Ferreira D, Nordberg A, Westman E. Biological subtypes of Alzheimer disease: A systematic review and meta-analysis. *Neurology*. 2020;94(10):436-448. doi:10.1212/WNL.0000000000009058

5. Jellinger KA. Pathobiological Subtypes of Alzheimer Disease. *Dementia and Geriatric Cognitive Disorders*. 2021;49(4):321-333. doi:10.1159/000508625

6. Murray ME, Graff-Radford NR, Ross OA, Petersen RC, Duara R, Dickson DW. Neuropathologically defined subtypes of Alzheimer's disease with distinct clinical characteristics: a retrospective study. *The Lancet Neurology*. 2011;10(9):785-796. doi:10.1016/S1474-4422(11)70156-9

7. Oxtoby NP, Alexander DC. Imaging plus X: multimodal models of neurodegenerative disease. *Current Opinion in Neurology*. 2017;30(4):371-379. doi:10.1097/WCO.0000000000000460

8. Birkenbihl C, Salimi Y, Fröhlich H. Unraveling the heterogeneity in Alzheimer's disease progression across multiple cohorts and the implications for data-driven disease modeling. *Alzheimer's and Dementia*. Published online 2021. doi:10.1002/alz.12387

9. Duara R. Heterogeneity in Alzheimer's Disease Diagnosis and Progression Rates: Implications for Therapeutic Trials. :18.

10. Blennow K, Zetterberg H. Biomarkers for Alzheimer's disease: current status and prospects for the future. *J Intern Med*. 2018;284(6):643-663. doi:10.1111/joim.12816

11. on behalf of Alzheimer's Disease Neuroimaging Initiative, Ou YN, Xu W, et al. FDG-PET as an independent biomarker for Alzheimer's biological diagnosis: a longitudinal study. *Alz Res Therapy*. 2019;11(1):57. doi:10.1186/s13195-019-0512-1

For Review Only

12. Vogel JW, Mattsson N, Iturria-Medina Y, et al. Data-driven approaches for tau-PET imaging biomarkers in Alzheimer's disease. *Human Brain Mapping*. 2019;40(2):638-651. doi:10.1002/hbm.24401

13. Frisoni GB, Fox NC, Jack CR, Scheltens P, Thompson PM. The clinical use of structural MRI in Alzheimer disease. *Nat Rev Neurol*. 2010;6(2):67-77. doi:10.1038/nrneurol.2009.215

14. Tsoi KKF, Chan JYC, Hirai HW, Wong SYS, Kwok TCY. Cognitive Tests to Detect Dementia: A Systematic Review and Meta-analysis. *JAMA Intern Med*. 2015;175(9):1450-1458. doi:10.1001/jamainternmed.2015.2152

15. Jack CR, Knopman DS, Jagust WJ, et al. Tracking pathophysiological processes in Alzheimer's disease: an updated hypothetical model of dynamic biomarkers. *The Lancet Neurology*. 2013;12(2):207-216. doi:10.1016/S1474-4422(12)70291-0

16. Khoury R, Ghossoub E. Diagnostic biomarkers of Alzheimer's disease: A state-of-the-art review. *Biomarkers in Neuropsychiatry*. 2019;1:100005. doi:10.1016/j.bionps.2019.100005

17. Zetterberg H, Bendlin BB. Biomarkers for Alzheimer's disease—preparing for a new era of disease-modifying therapies. *Mol Psychiatry*. 2021;26(1):296-308. doi:10.1038/s41380-020-0721-9

18. Oxtoby NP, Young AL, Cash DM, et al. Data-driven models of dominantly-inherited Alzheimer's disease progression. *Brain*. 2018;141(5):1529-1544. doi:10.1093/brain/awy050

19. Xiao R, Liu X, Qiao H, Zheng X, Zhang Y, Cui X. Adaptive LASSO logistic regression based on particle swarm optimization for Alzheimer's disease early diagnosis. *Chemometrics and Intelligent Laboratory Systems*. Published online April 16, 2021:104316. doi:10.1016/j.chemolab.2021.104316

20. Kühnel L, Berger AK, Markussen B, Raket LL. Simultaneous modeling of Alzheimer's disease progression via multiple cognitive scales. *Statistics in Medicine*. n/a(n/a). doi:https://doi.org/10.1002/sim.8932

21. Mohanty R, Mårtensson G, Poulakis K, et al. Towards harmonizing subtyping methods for neuroimaging studies in Alzheimer's disease. *medRxiv*. Published online April 23, 2020:2020.04.19.20064881. doi:10.1101/2020.04.19.20064881

22. Vogel JW, Young AL, Oxtoby NP, et al. Four distinct trajectories of tau deposition identified in Alzheimer's disease. *Nat Med*. 2021;27(5):871-881. doi:10.1038/s41591-021-01309-6

23. Aksman LM, Oxtoby NP, Scelsi MA, et al. Tau-first subtype of Alzheimer's disease progression consistently identified through PET and CSF. *Alzheimer's & Dementia*. 2020;16(S5):e045412. doi:https://doi.org/10.1002/alz.045412

24. Thung KH, Wee CY, Yap PT, Shen D. Neurodegenerative disease diagnosis using incomplete multi-modality data via matrix shrinkage and completion. *NeuroImage*. 2014;91:386-400. doi:10.1016/j.neuroimage.2014.01.033

For Review Only

25. Fonteijn HM, Modat M, Clarkson MJ, et al. An event-based model for disease progression and its application in familial Alzheimer's disease and Huntington's disease. *NeuroImage*. 2012;60(3):1880-1889. doi:10.1016/j.neuroimage.2012.01.062

26. Young AL, Oxtoby NP, Daga P, et al. A data-driven model of biomarker changes in sporadic Alzheimer's disease. *Brain*. 2014;137(9):2564-2577. doi:10.1093/brain/awu176

27. Young AL, Vogel JW, Aksman LM, et al. Ordinal SuStaIn: Subtype and Stage Inference for Clinical Scores, Visual Ratings, and Other Ordinal Data. *Front Artif Intell*. 2021;4:613261. doi:10.3389/frai.2021.613261

28. Firth NC, Primativo S, Brotherhood E, et al. Sequences of cognitive decline in typical Alzheimer's disease and posterior cortical atrophy estimated using a novel event-based model of disease progression. *Alzheimer's & Dementia*. 2020;16(7):965-973. doi:10.1002/alz.12083

29. Aksman LM, Wijeratne PA, Oxtoby NP, et al. pySuStaIn: A Python implementation of the Subtype and Stage Inference algorithm. *SoftwareX*. 2021;16:100811. doi:10.1016/j.softx.2021.100811

30. Altman NS. An introduction to kernel and nearest-neighbor nonparametric regression. *The American Statistician*. 1992;46(3):175-185.

31. Rosen, Wilma G and Mohs, Richard C and Davis, Kenneth L. A new rating scale for Alzheimer's disease. Published online 1984.

32. Rey A. *L'examen Clinique En Psychologie. [The Clinical Examination in Psychology.]*. Presses Universitaries De France; 1958:222.

33. Battery AIT. Manual of directions and scoring. Published online 1944.

34. Shaw LM, Vanderstichele H, Knapik-Czajka M, et al. Cerebrospinal fluid biomarker signature in Alzheimer's disease neuroimaging initiative subjects. *Ann Neurol*. 2009;65(4):403-413. doi:10.1002/ana.21610

35. Gibbons LE, Carle AC, Mackin RS, et al. A composite score for executive functioning, validated in Alzheimer's Disease Neuroimaging Initiative (ADNI) participants with baseline mild cognitive impairment. *Brain Imaging and Behavior*. 2012;6(4):517-527. doi:10.1007/s11682-012-9176-1

36. Crane PK, Carle A, Gibbons LE, et al. Development and assessment of a composite score for memory in the Alzheimer's Disease Neuroimaging Initiative (ADNI). *Brain Imaging and Behavior*. 2012;6(4):502-516. doi:10.1007/s11682-012-9186-z

37. DeCarli C, Maillard P, Fletcher E. Four Tissue Segmentation in ADNI II. Published online 2013:6.

38. Cedres N, Ekman U, Poulakis K, et al. Brain Atrophy Subtypes and the ATN Classification Scheme in Alzheimer's Disease. *Neurodegenerative Diseases*. 2021;20(4):153-164. doi:10.1159/000515322

For Review Only

39. Ferreira D, Verhagen C, Hernández-Cabrera JA, et al. Distinct subtypes of Alzheimer's disease based on patterns of brain atrophy: Longitudinal trajectories and clinical applications. *Scientific Reports*. 2017;7. doi:10.1038/srep46263

40. Persson K, Eldholm RS, Barca ML, et al. MRI-assessed atrophy subtypes in Alzheimer's disease and the cognitive reserve hypothesis. *PLoS ONE*. 2017;12(10). doi:10.1371/journal.pone.0186595

41. Archetti D, Young AL, Oxtoby NP, et al. Inter-Cohort Validation of SuStaIn Model for Alzheimer's Disease. *Frontiers in Big Data*. 2021;4. Accessed October 21, 2022. https://www.frontiersin.org/articles/10.3389/fdata.2021.661110

42. Levin F, Ferreira D, Lange C, et al. Data-driven FDG-PET subtypes of Alzheimer's disease-related neurodegeneration. *Alzheimer's Research and Therapy*. 2021;13(1). doi:10.1186/s13195-021-00785-9

43. Ekman U, Ferreira D, Westman E. The A/T/N biomarker scheme and patterns of brain atrophy assessed in mild cognitive impairment. *Sci Rep*. 2018;8(1):8431. doi:10.1038/s41598-018-26151-8

44. Pillai JA, Bonner-Jackson A, Bekris LM, Safar J, Bena J, Leverenz JB. Highly Elevated Cerebrospinal Fluid Total Tau Level Reflects Higher Likelihood of Non-Amnestic Subtype of Alzheimer's Disease. *Journal of Alzheimer's Disease*. 2019;70(4):1051-1058. doi:10.3233/JAD-190519

45. Granadillo E, Paholpak P, Mendez MF, Teng E. Visual Ratings of Medial Temporal Lobe Atrophy Correlate with CSF Tau Indices in Clinical Variants of Early-Onset Alzheimer Disease. *Dement Geriatr Cogn Disord*. 2017;44(1-2):45-54. doi:10.1159/000477718

46. Ossenkoppele R, Mattsson N, Teunissen CE, et al. Cerebrospinal fluid biomarkers and cerebral atrophy in distinct clinical variants of probable Alzheimer's disease. *Neurobiol Aging*. 2015;36(8):2340-2347. doi:10.1016/j.neurobiolaging.2015.04.011

47. ten Kate M, Dicks E, Visser PJ, et al. Atrophy subtypes in prodromal Alzheimer's disease are associated with cognitive decline. *Brain*. 2018;141(12):3443-3456. doi:10.1093/brain/awy264

48. Stern Y. Cognitive reserve in ageing and Alzheimer's disease. *The Lancet Neurology*. 2012;11(11):1006-1012. doi:10.1016/S1474-4422(12)70191-6

49. Lee DH, Seo SW, Roh JH, et al. Effects of Cognitive Reserve in Alzheimer's Disease and Cognitively Unimpaired Individuals. *Front Aging Neurosci*. 2022;13:784054. doi:10.3389/fnagi.2021.784054

50. Zhang B, Lin L, Wu S, Al-masqari ZHMA. Multiple subtypes of alzheimer's disease base on brain atrophy pattern. *Brain Sciences*. 2021;11(2):1-15. doi:10.3390/brainsci11020278

51. Whitwell JL, Dickson DW, Murray ME, et al. Neuroimaging correlates of pathologically defined subtypes of Alzheimer's disease: A case-control study. *The Lancet Neurology*. 2012;11(10):868-877. doi:10.1016/S1474-4422(12)70200-4

For Review Only

52. Wijeratne PA, Alexander DC. Learning transition times in event sequences: the Event-Based Hidden Markov Model of disease progression. :8.

Figure legends

Figure 1 SuStaIn subtypes identified in the ADNI dataset. This figure depicts the five subtypes uncovered by SuStaIn and their respective progression patterns. At each stage, the colour indicates the level of change compared to controls: white means no change; red means a change of z-score = 1; magenta, z-score = 2; blue, z-score = 3 and black, z-score = 5. The y-axis shows the different biomarkers used for the study, while the x-axis shows the position of the z-score events for each biomarker, which ranges from 1 to 45. Insula, Amygdala, Hippocampus, Thalamus, FrontalLobe, ParietalLobe, TemporalLobe, OccipitalLobe, Cingulate and BasalGanglia correspond to MRI measurements. ABETA, TAU and PTAU are CSF markers, while FDG and AV45 correspond to PET data. ADAS13, RAVLT and TRABSCOR are the three cognitive test scores used in this study. N corresponds to the number of subjects belonging to each subtype, excluding those that were assigned to the Normal Appearing subtype (SuStaIn stage 0).

Figure 2 Two San Key flows showing subtyping consistency between models. A Normal Appearing subtype was included, consisting of those subjects assigned to Stage 0. Diagram A compares the 406 Aβ+ subjects, subtyped by the Aβ+ (left) and full data model (right). Diagram B compares the 275 Aβ- subjects, subtyped by the Aβ- (right) and full data model (left).

Figure 3. A - F show plots depicting the differences in education, age, gender, diagnosis, APOE status and stage between SuStaIn subtypes. A Normal Appearing subtype was added in all experiments, representing those people in stage zero. **(A)** Boxplots showing the distribution of education years in all different subtypes. Significant differences between groups computed via t-tests are marked with three stars for a p-value < 0.001, two stars for p-value < 0.01 and one star for p-value< 0.05. Subjects assigned to 'Cognitive' subtype are seen to be those with less education years. **(B)** Boxplot depicting the age distribution in subtypes. Those subjects assigned to Normal Appearing subtype are significantly younger than all other subtypes, except the 'Cortical' one. Those belonging to the 'Cognitive' subtype

For Review Only

appear to be the oldest. A statistically significant difference is marked with asterisk as in figure 2.A. **(C)** Bar plot showing the proportion of female and male subjects in every subtype. No significant differences are found between subtypes and gender proportion. **(D)** Bar plot depicting the proportion of subjects with zero, one or two APOE4 alleles in each subtype. The two 'Typical' and the 'Cognitive' subtypes have the highest proportion of subjects presenting with two APOE4 alleles. **(E)** Bar plot showing the proportion of amyloid positive subjects in each subtype, where a significantly higher proportion of amyloid positive subjects can be seen in the two 'Typical' subtypes. **(F)** This bar plot shows the proportion of subjects diagnosed as MCI, AD or CN in ADNI; divided by subject. The highest proportion of AD diagnosed people can be found in subtype 'Typical AD Late Tau', followed by the 'Cognitive' subtype. **(G)** Box plot showing white matter hyperintensity volume differences

Figure 4. Boxplots showing subtype differences in two cognitive batteries. (A) Memory Function and **(B)** Executive Function

Figure 5 MCI to AD conversion Kaplan Meier curve for each subtype. Kaplan Meier model was fitted with all baseline MCI subjects, only baseline MCI subjects with complete data, and only baseline MCI subjects with missing data. The prediction of conversion patterns for each subtype is maintained in the different data subsets.

For Review Only

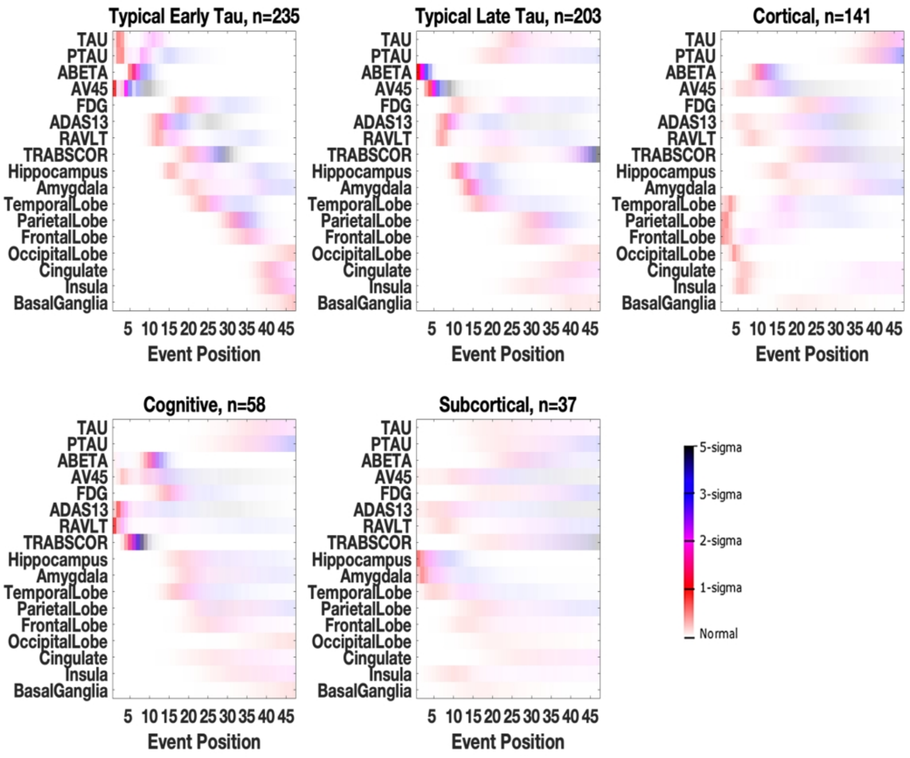

Figure 1 SuStaIn subtypes identified in the ADNI dataset. This figure depicts the five subtypes uncovered by SuStaIn and their respective progression patterns. At each stage, the colour indicates the level of change compared to controls: white means no change; red means a change of z-score = 1; magenta, z-score = 2; blue, z-score = 3 and black, z-score = 5. The y-axis shows the different biomarkers used for the study, while the x-axis shows the position of the z-score events for each biomarker, which ranges from 1 to 45. Insula, Amygdala, Hippocampus, Thalamus, FrontalLobe, ParietalLobe, TemporalLobe, OccipitalLobe, Cingulate and BasalGanglia correspond to MRI measurements. ABETA, TAU and PTAU are CSF markers, while FDG and AV45 correspond to PET data. ADAS13, RAVLT and TRABSCOR are the three cognitive test scores used in this study. N corresponds to the number of subjects belonging to each subtype, excluding those that were assigned to the Normal Appearing subtype (SuStaIn stage 0).

Figure 1 SuStaIn subtypes identified in the ADNI dataset. This figure depicts the five subtypes uncovered by SuStaIn and their respective progression patterns. At each stage, the colour indicates the level of change compared to controls: white means no change; red means a change of z-score = 1; magenta, z-score = 2; blue, z-score = 3 and black, z-score = 5. The y-axis shows the different biomarkers used for the study, while the x-axis shows the position of the z-score events for each biomarker, which ranges from 1 to 45. Insula, Amygdala, Hippocampus, Thalamus, FrontalLobe, ParietalLobe, TemporalLobe, OccipitalLobe, Cingulate and BasalGanglia correspond to MRI measurements. ABETA, TAU and PTAU are CSF markers, while FDG and AV45 correspond to PET data. ADAS13, RAVLT and TRABSCOR are the three cognitive test scores used in this study. N corresponds to the number of subjects belonging to each subtype, excluding those that were assigned to the Normal Appearing subtype (SuStaIn stage 0).

427x488mm (130 x 130 DPI)

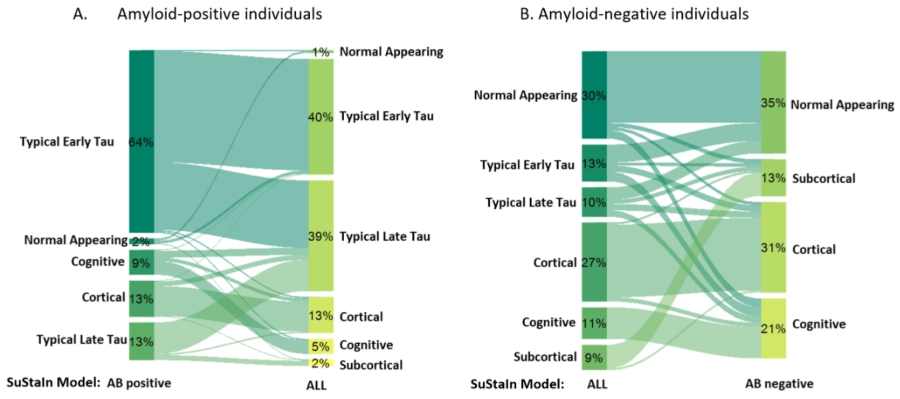

Figure 2 Two San Key flows showing subtyping consistency between models. A Normal Appearing subtype was included, consisting of those subjects assigned to Stage 0. Diagram A compares the 406 Aβ+ subjects, subtyped by the Aβ+ (left) and full data model (right). Diagram B compares the 275 Aβ- subjects, subtyped by the Aβ- (right) and full data model (left).

Figure 2 Two San Key flows showing subtyping consistency between models. A Normal Appearing subtype was included, consisting of those subjects assigned to Stage 0. Diagram A compares the 406 Aβ+ subjects, subtyped by the Aβ+ (left) and full data model (right). Diagram B compares the 275 Aβ- subjects, subtyped by the Aβ- (right) and full data model (left).

545x345mm (130 x 130 DPI)

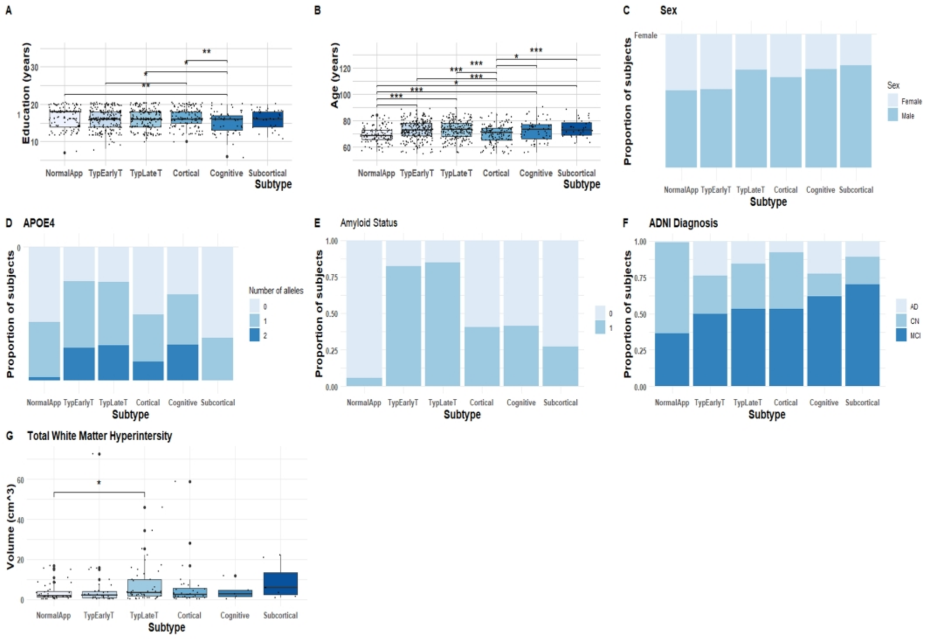

Figure 3. A - F show plots depicting the differences in education, age, gender, diagnosis, APOE status and stage between SuStaIn subtypes. A Normal Appearing subtype was added in all experiments, representing those people in stage zero. **(A)** Boxplots showing the distribution of education years in all different subtypes. Significant differences between groups computed via t-tests are marked with three stars for a p-value < 0.001, two stars for p-value < 0.01 and one star for p-value< 0.05. Subjects assigned to 'Cognitive' subtype are seen to be those with less education years. **(B)** Boxplot depicting the age distribution in subtypes. Those subjects assigned to Normal Appearing subtype are significantly younger than all other subtypes, except the 'Cortical' one. Those belonging to the 'Cognitive' subtype appear to be the oldest. A statistically significant difference is marked with asterisk as in figure 2.A. **(C)** Bar plot showing the proportion of female and male subjects in every subtype. No significant differences are found between subtypes and gender proportion. **(D)** Bar plot depicting the proportion of subjects with zero, one or two APOE4 alleles in each subtype. The two 'Typical' and the 'Cognitive' subtypes have the highest proportion of subjects presenting with two APOE4 alleles. **(E)** Bar plot showing the proportion of amyloid positive subjects in each subtype, where a significantly higher proportion of amyloid positive subjects can be seen in the two 'Typical' subtypes. **(F)** This bar plot shows the proportion of subjects diagnosed as MCI, AD or CN in ADNI; divided by subject. The highest proportion of AD diagnosed people can be found in subtype 'Typical AD Late Tau', followed by the 'Cognitive' subtype. **(G)** Box plot showing white matter hyperintensity volume differences

Figure 3. A - F show plots depicting the differences in education, age, gender, diagnosis, APOE status and stage between SuStaIn subtypes. A Normal Appearing subtype was added in all experiments, representing those people in stage zero. (A) Boxplots showing the distribution of education years in all different subtypes. Significant differences between groups computed via t-tests are marked with three stars for a p-value < 0.001, two stars for p-value < 0.01 and one star for p-value< 0.05. Subjects assigned to 'Cognitive' subtype are seen to be those with less education years. (B) Boxplot depicting the age distribution in subtypes. Those subjects assigned to Normal Appearing subtype are significantly younger than all other subtypes, except the 'Cortical' one. Those belonging to the 'Cognitive' subtype appear to be the oldest. A statistically significant difference is marked with asterisk as in figure 2.A. (C) Bar plot showing the proportion of female and male subjects in every subtype. No significant differences are found between subtypes and gender proportion. (D) Bar plot depicting the proportion of subjects with zero, one or two APOE4 alleles in each subtype. The two 'Typical' and the 'Cognitive' subtypes have the highest proportion of subjects presenting with two APOE4 alleles. (E) Bar plot showing the proportion of amyloid positive subjects in each subtype, where a significantly higher proportion of amyloid positive subjects can be seen in the two 'Typical' subtypes. (F) This bar plot shows the proportion of subjects diagnosed as MCI, AD or CN in ADNI; divided by subject. The highest proportion of AD diagnosed people can be found in subtype 'Typical AD Late Tau', followed by the 'Cognitive' subtype. (G) Box plot showing white matter hyperintensity volume differences.

599x565mm (130 x 130 DPI)

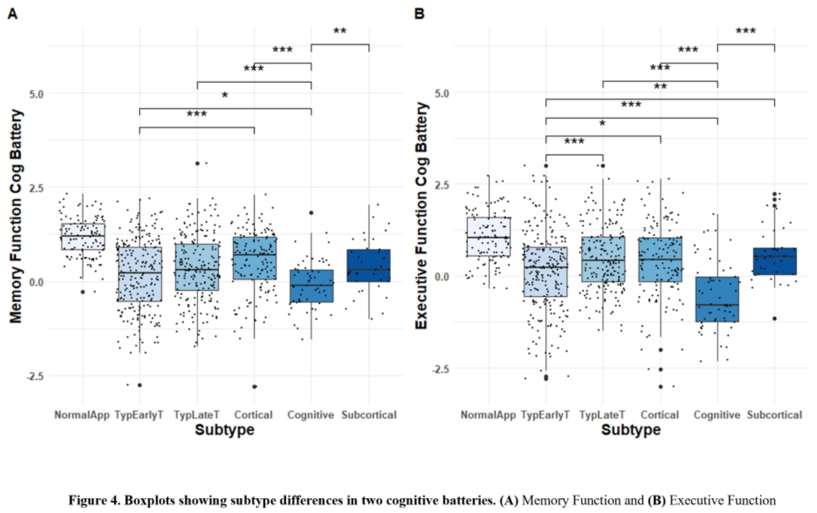

Figure 4. Boxplots showing subtype differences in two cognitive batteries. (A) Memory Function and (B) Executive Function

521x348mm (130 x 130 DPI)

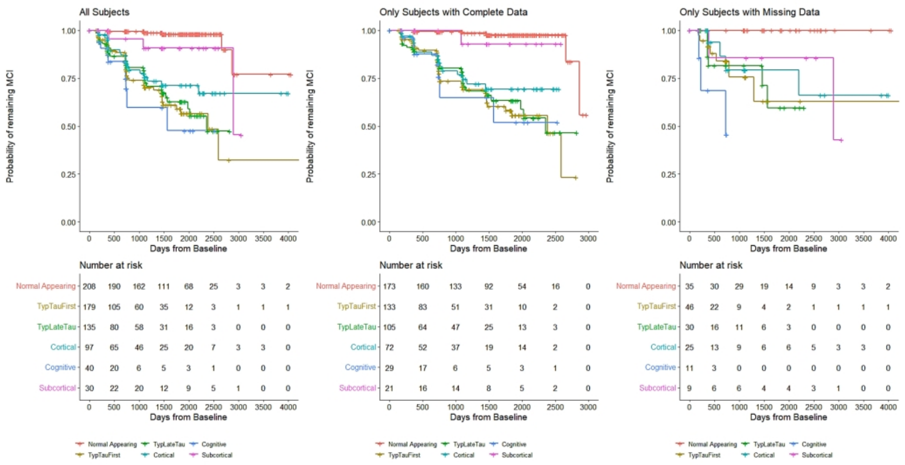

Figure 5 MCI to AD conversion Kaplan Meier curve for each subtype. Kaplan Meier model was fitted with all baseline MCI subjects, only baseline MCI subjects with complete data, and only baseline MCI subjects with missing data. The prediction of conversion patterns for each subtype is maintained in the different data subsets.

Figure 5 MCI to AD conversion Kaplan Meier curve for each subtype. Kaplan Meier model was fitted with all baseline MCI subjects, only baseline MCI subjects with complete data, and only baseline MCI subjects with missing data. The prediction of conversion patterns for each subtype is maintained in the different data subsets.

545x393mm (130 x 130 DPI)

Table 1 Assessment of performance of different methods for handling missing data. Comparison metrics include the similarity between the estimated subtype progression patterns and the ground truth, and subject staging and subtyping accuracy. Similarity between the three progression patterns is measured using the Kendall Tau coefficient, with 0 indicating no similarity between the progression patterns and 1 indicating identical progression patterns. The last experiment, 'Full data' benchmarks the performance when there is no missing data to indicate the best possible performance. The mean Kendall Tau coefficient across the three progression patterns is stated in this table; for a breakdown by subtype refer to Table S1. The subtyping and staging metrics are reported across all individuals (with and without missing data points); subtyping and staging metrics computed only in the subset of subjects with missing data can be found in Table S2.

Missing Data	Progression pattern similarity<br>Mean Kendal Tau Distance	Subtype<br>% correct	Stage<br>Mean diff (stand dev)
Deletion	0.89	79.60	1.84 (1.64)
Mean	0.92	84.20	1.57 (1.32)
KNN Biomarkers (feature matrix)	0.91	84.80	1.60 (1.36)
KNN Biomarkers (Z-scored)	0.44	60.60	14.25(7.54)
Uniform distribution	0.93	85.40	1.53 (1.30)
Full Data	0.923	85.80	1.54 (1.31)

For Review Only

Table 2 Variable of age, education, sex, APOE4 alleles, amyloid status and ADNI diagnosis divided by SuStaIn subtype. Statistical significance is computed between each subtype vs. all the rest. Significant differences are marked with three stars for a p-value < 0.001, two stars for p-value < 0.01 and one star for p-value< 0.05

Subtypes → Variables ↓	Normal Appearing (115)	Typical AD Early Tau (235)	Typical AD Late Tau (203)	Cortical (141)	Cognitive (58)	Subcortical (37)
Age, years mean (st dev)	69.5 (6.2)*** p-value = 2.69e-05	72.7 (7.0)* p-value = 0.02	73.0 (6.8)** p-value = 0.005	69.9 (7.0)** p-value = 0.003	72.3 (7.9)	74.29 (6.3)** p-value = 0.02
Education, years mean (st dev)	16.5 (2.7)	15.9 (2.8)	16.2 (2.5)	16.6 (2.5)** p-value = 0.03	15.2 (2.9)** p-value = 0.009	16.23 (2.4)
Sex , % female	59.1	58.3	41.9	48.9	41.4	37.8
APOE4 alleles % positive	27.7	56	55.4	30.7	43.8	18.9
Amyloid Status, % positive	5.7	82.3	84.9	40.5	41.2	27.3
ADNI Diagnosis % of total n						
AD	0.8	23.8	15.8	7.8	22.4	10.8
CN	62.6	26.4	31.0	39.0	15.5	18.9
MCI	36.5	49.8	53.2	53.2	62.1	70.3
Cognitive Batteries Memory Function (mean)	1.1*** p=7.52e-37	0.2*** p=1.81e-06	0.3	0.5	-0.1*** p=7.68e-08	0.4
Executive Function (mean)	1.1*** p=7.61e-21	0.1*** p=2.99e-05	0.5	0.4	-0.6*** p=2.94e-11	0.6
Total WMHV at baseline (mean)	3.6** p=0.01	5.9	7.5	5.9	4.2	8.6
Age at death (mean, (n))	79, (1)	78, (8)	76.8, (5)	82.2, (4)	83.5, (6)	93, (1)

For Review Only

Supplementary material

A. SuStaIn results in ADNI divided according to amyloid status

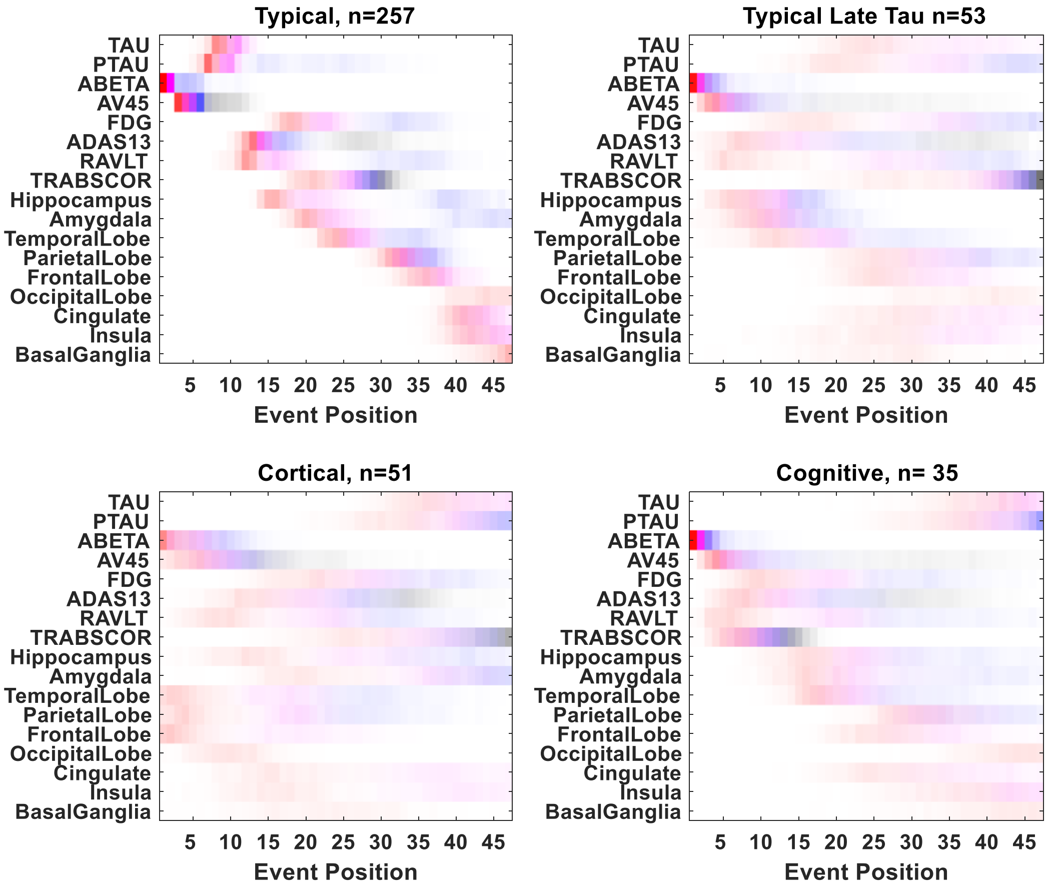

Figure S1 SuStaIn modelling of baseline amyloid positive subjects in ADNI data. Four different subtypes are uncovered: 'Typical, 'Typical Late Tau', 'Cortical' and 'Cognitive', in order of prevalence. Subtypes found coincide with those fitting the model to both amyloid positive and negative subjects, except in the missing 'Subcortical' subtype found in the previous model (see Figure 1).

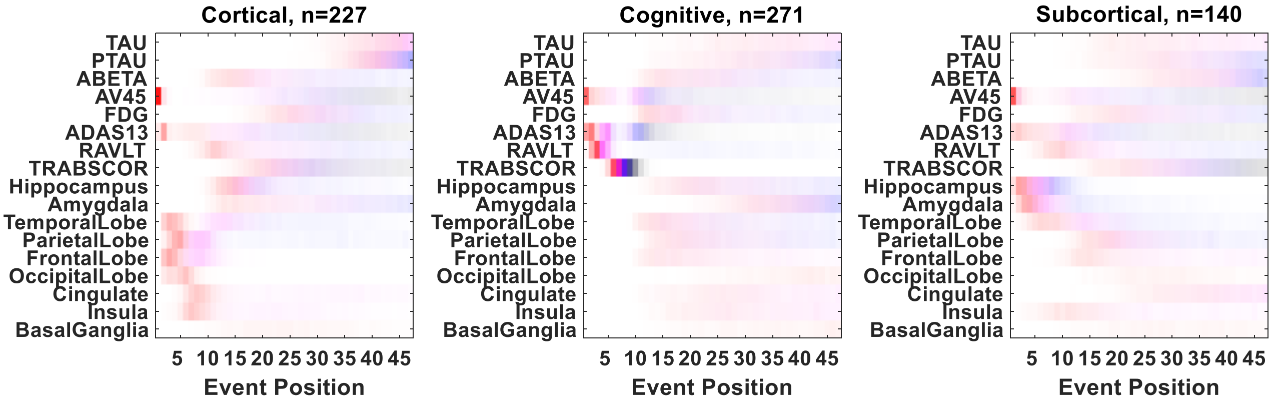

Figure S2 Three subtypes uncovered by SuStaIn fitted using only amyloid negative subjects. 'Cortical', 'Cognitive' and 'Subcortical' coincide with the three non-typical AD progression patterns found in the model fitted with all the subjects (amyloid positive and negative).

B. Prediction of Conversion

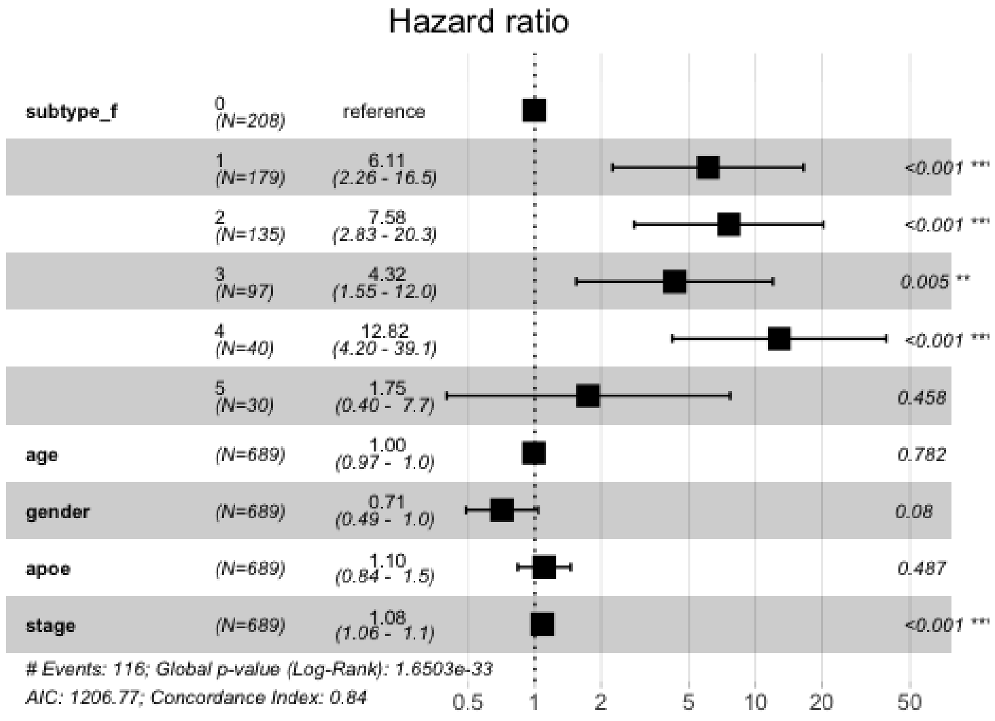

Figure S3 Hazard ratios for Cox Proportional Hazards model, estimating the risk of MCI to AD conversion. Each row shows the estimated hazard ratio with 95% confidence intervals, for each covariable accounted in the model.

C. Supplementary analysis

To further test the significance of using multimodal data for this study, subtyping and staging of subjects at baseline was studied and compared using the full set of biomarkers versus deleting one type of modality. As CSF and PET convey information about similar pathological processes, deleting both was also explored. Figure S4 and Table S1 suggest that CSF and PET data are the most important features for subtyping. We hypothesize that this is because they are the only early AD specific markers that were included in the study. MRI was seen to be the most influential biomarker for staging, showing that when it is not present, individuals tend to be assigned in higher SuStaIn stages. Suggesting, as we might suspect, that MRI best reflects progressive changes throughout the disease course [1,2].

We also studied how consistent subtyping and staging was from baseline to later visits exploring the effect of missing data and confidence of assignment of subtype. Subjects were divided depending on different ranges of confidence of subtype (90%, 70-90%, 50-70%, 30-50% and up to 30%). Results showed in Figure S5 indicate that 86.0% of individuals were classified in the same subtype in following visits when the range of confidence of subtype is above 90%. This value drops proportionally to the confidence of subtype assignment.

For Review Only

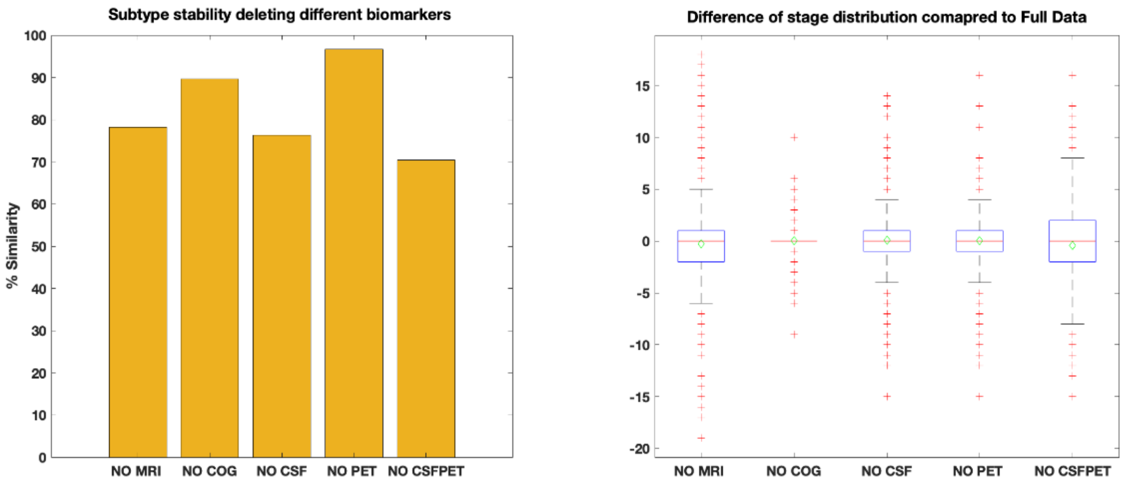

Figure S4. Importance of different modalities for subtyping and staging (A). Bar chart bar chart of stability of subtypes under deletion of a biomarker. Similarity is measured comparing the subtype assignment of using the full dataset (with the four types of biomarkers) with those assigned when deleting a biomarker **(B)** Box plots showing distribution of the difference of stages between using the full range of biomarkers and deleting MRI, COG, CSF, PET or CSF and PET

Table S1. Accuracy of confidence estimates of subtypes and stages when deleting a modality. Comparisons are computed between results obtained using subjects with a full dataset (598 out of the total 789) versus when deleting a modality

**Data (Only those with full data)**	**Percentage common Subtypes in 95%CI**	**Average of Subtype CI "width"**	**Percentage common Stages in 95%CI**	**Average of Stage CI "width"**
**All**	-	**1.74**	-	**8.77**
Missing MRI	92.31	2.15	100	15.32
Missing COG	92.64	1.94	100	10.68
Missing CSF	94.64	1.87	100	9.53
Missing PET	97.82	1.78	100	9.41
Missing PET and CSF	93.14	2.21	100	12.22

Prob subtype: 90-100% Consistency: 86.30%

Subtype Later Visit \ Subtype at Baseline	NA	TTF	TLT	CORT	COG	SUBC	
NA	0	0	0	0	0	0	
TTF	1	19	2	0	1	0	86,96 %
TLT	0	2	10	0	0	2	71,43 %
CORT	2	1	2	82	1	0	95,45 %
COG	0	6	1	1	8	0	50,00 %
SUBC	0	1	0	0	0	4	80,00 %

Prob subtype: 70-90% Consistency: 72.31%

Subtype Later Visit \ Subtype at Baseline	NA	TTF	TLT	CORT	COG	SUBC	
NA	0	0	0	0	0	0	
TTF	1	45	13	0	3	0	74,19 %
TLT	3	15	50	1	1	12	64,63 %
CORT	3	7	10	67	3	0	77,78 %
COG	0	1	0	0	1	1	33,33 %
SUBC	0	0	0	0	0	5	100,00 %

Prob subtype: 50-70% Consistency: 50.74%

Subtype Later Visit \ Subtype at Baseline	NA	TTF	TLT	CORT	COG	SUBC	
NA	117	73	70	2	10	0	43,01 %
TTF	41	166	101	30	37	8	54,05 %
TLT	1	27	44	6	1	17	46,88 %
CORT	9	21	12	51	1	1	63,16 %
COG	0	6	4	2	12	0	50,00 %
SUBC	0	1	4	1	0	7	53,85 %

Prob subtype: 30-50% Consistency: 43.93%

Subtype Later Visit \ Subtype at Baseline	NA	TTF	TLT	CORT	COG	SUBC	
NA	94	38	55	3	15	0	45,85 %
TTF	21	101	78	57	25	15	41,08 %
TLT	1	23	32	12	5	9	40,24 %
CORT	4	19	13	35	1	2	52,70 %
COG	3	5	4	1	4	0	41,18 %
SUBC	1	1	3	4	0	8	52,94 %

Prob subtype: 0-30% Consistency: 30%

Subtype Later Visit \ Subtype at Baseline	NA	TTF	TLT	CORT	COG	SUBC	
NA	1	0	1	0	0	0	50,00 %
TTF	0	2	0	1	0	0	66,67 %
TLT	0	7	1	4	0	0	8,33 %
CORT	0	0	1	1	0	0	50,00 %
COG	0	0	0	0	1	0	100,00 %
SUBC	0	0	0	0	0	0	

Figure S5 Longitudinal subtype consistency stratified by the probability of the subtype assignment. A high probability of 90% or above gave a longitudinal consistency of 86.3%. This percentage decreased between 10 to 20 percent each time the probability of belonging to a subtype was lowered to 70, 50, 30 and <30.

Table S2 95% confidence interval similarity study depending on confidence of subtype assignment shows that even if the consistency decreases when looking at the most prevalent subtype, the 95% confidence interval remains high throughout all probabilities of assignment. How many subtypes fall into that 95% confidence intervals do increase to almost 4 when the probability of being assigned to a subtype decrease to less than 0.3

Confidence of subtype assignment	1-0.9	0.9.- 0.7	0.7- 0.5	0.5-0.3	<0.3
At least one common subtype (%)	100	100	100	100	100
Mean “length” 95CI subtype	1.01	1.62	2.44	2.69	3.90

For Review Only
