# Supplementary material for: Multimodal subtypes identified in Alzheimer’s Disease Neuroimaging Initiative participants by missing-data-enabled subtype and stage inference
Source: Brain Commun. 2024 Jun 25;6(4):fcae219. doi: 10.1093/braincomms/fcae219 (PMC11259979; doi:10.1093/braincomms/fcae219)
Supplement: fcae219_Supplementary_Data [file fcae219_supplementary_data.zip › Supplementary_Material.pdf]

Supplementary Material

SuStaIn results in ADNI divided according to amyloid status.

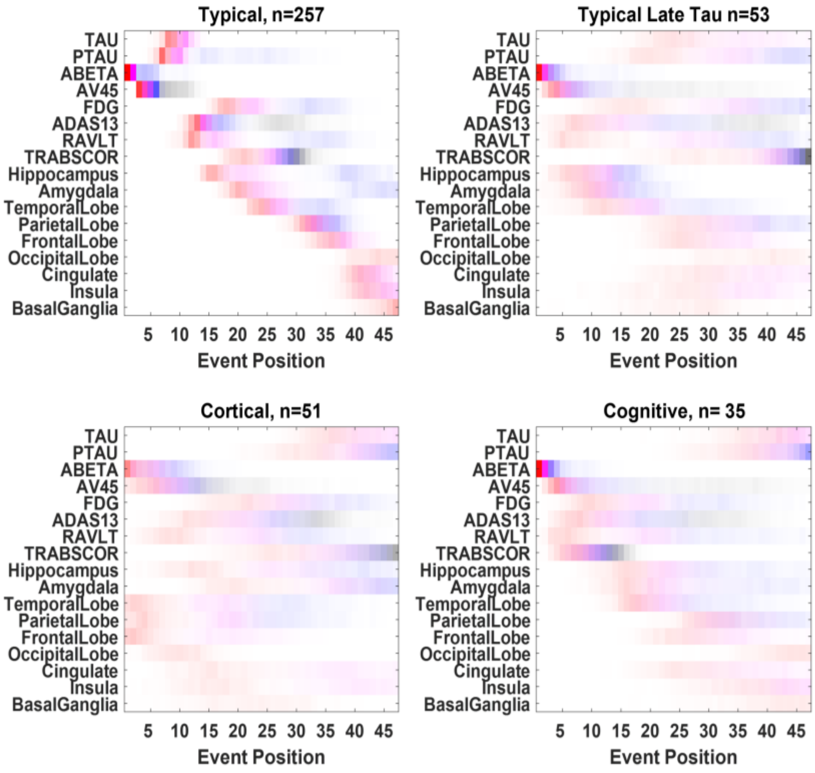

Supplementary Figure 1: SuStaIn modelling of baseline amyloid positive subjects in ADNI data. Four different subtypes are uncovered: 'Typical, 'Typical Late Tau', 'Cortical' and 'Cognitive', in order of prevalence. Subtypes found coincide with those fitting the model to both amyloid positive and negative subjects, except in the missing 'Subcortical' subtype found in the previous model (see Figure 1).

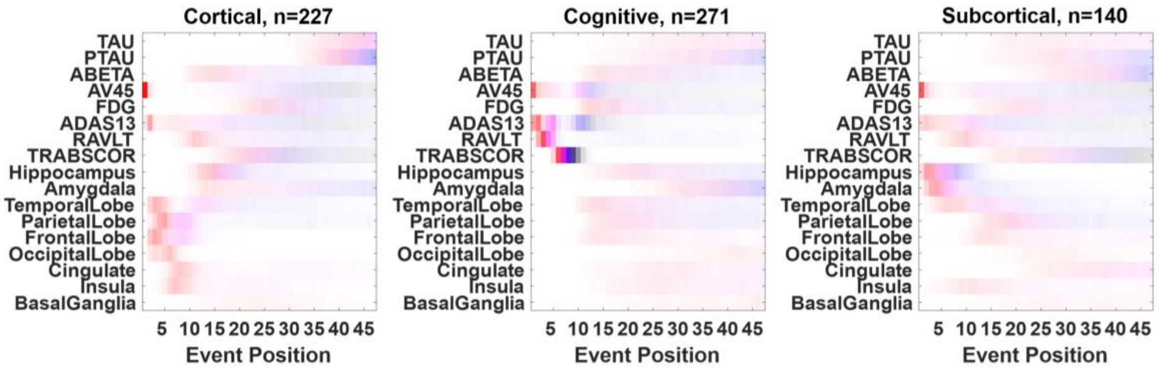

Supplementary Figure 2: Three subtypes uncovered by SuStaIn fitted using only amyloid negative subjects. 'Cortical', 'Cognitive' and 'Subcortical' coincide with the three non-typical AD progression patterns found in the model fitted with all the subjects (amyloid positive and negative).

Prediction of Conversion

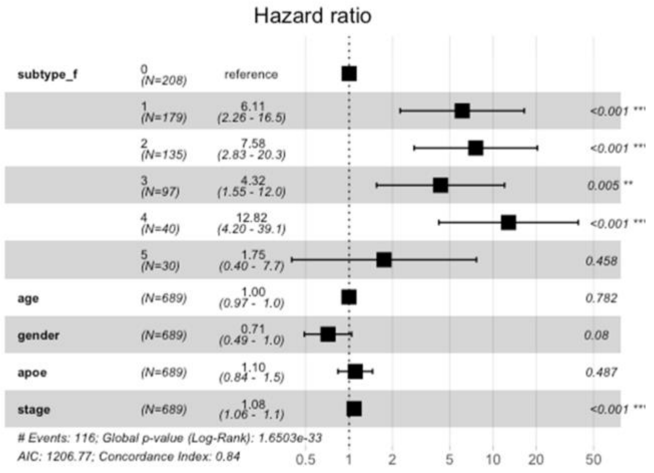

Supplementary Figure 3: Hazard ratios for Cox Proportional Hazards model, estimating the risk of MCI to AD conversion. Each row shows the estimated hazard ratio with 95% confidence intervals, for each covariable accounted in the model.

Supplementary analysis

To further test the significance of using multimodal data for this study, subtyping and staging of subjects at baseline was studied and compared using the full set of biomarkers versus deleting one type of modality. As CSF and PET convey information about similar pathological processes, deleting both was also explored. Supplementary Figure 4 and Supplementary Table 1 suggest that CSF and PET data are the most important features for subtyping. We hypothesize that this is because they are the only early AD specific markers that were included in the study. MRI was seen to be the most influential biomarker for staging, showing that when it is not present, individuals tend to be assigned in higher SuStaIn stages. Suggesting, as we might suspect, that MRI best reflects progressive changes throughout the disease course [1,2].

We also studied how consistent subtyping and staging was from baseline to later visits exploring the effect of missing data and confidence of assignment of subtype. Subjects were divided depending on different ranges of confidence of subtype (90%, 70-90%, 50-70%, 30-50% and up to 30%). Results showed in Supplementary Figure 5 indicate that 86.0% of individuals were classified in the same subtype in following visits when the range of confidence of subtype is above 90%. This value drops proportionally to the confidence of subtype assignment. Supplementary Table 2 shows that even if the consistency decreases when looking at the most prevalent subtype, the 95% confidence interval remains high throughout all probabilities of assignment.

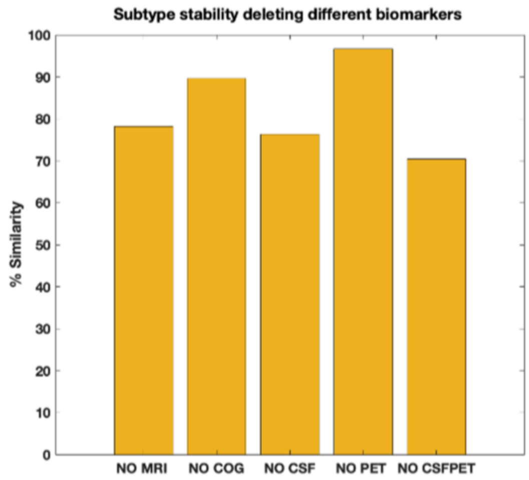

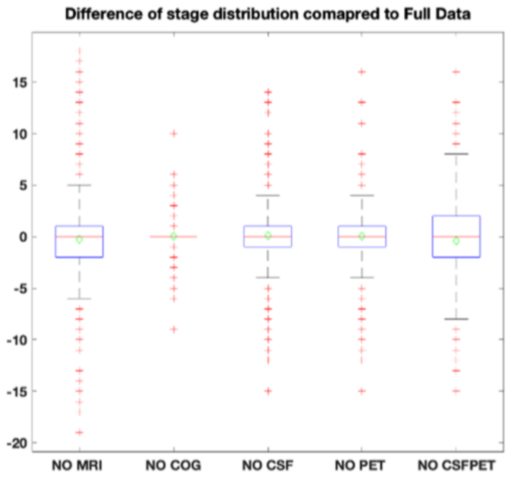

Supplementary Figure 4: Importance of different modalities for subtyping and staging (A). Bar chart bar chart of stability of subtypes under deletion of a biomarker. Similarity is measured comparing the subtype assignment of using the full dataset (with the four types of biomarkers) with those assigned when deleting a biomarker (B) Box plots showing distribution of the difference of stages between using the full range of biomarkers and deleting MRI, cognitive scores (COG), cerebrospinal fluid (CSF), positron emission tomography (PET) or CSF and PET together.

Supplementary Table 1: Accuracy of confidence estimates of subtypes and stages when deleting a modality. Comparisons are computed between results obtained using subjects with a full dataset (598 out of the total 789) versus when deleting a modality

Data (Only those with full data)	Percentage common Subtypes in 95%CI	Average of Subtype CI "width"	Percentage common Stages in 95%CI	Average of Stage CI "width"
**All**	-	**1.74**	-	**8.77**
Missing MRI	92.31	2.15	100	15.32
Missing COG	92.64	1.94	100	10.68
Missing CSF	94.64	1.87	100	9.53
Missing PET	97.82	1.78	100	9.41
Missing PET and CSF	93.14	2.21	100	12.22

Supplementary Table 2: 95% confidence interval similarity study depending on confidence of subtype assignment shows that even if the consistency decreases when looking at the most prevalent subtype, the 95% confidence interval remains high throughout all probabilities of assignment. How many subtypes fall into that 95% confidence intervals do increase to almost 4 when the probability of being assigned to a subtype decrease to less than 0.3

Confidence of subtype assignment	1-0.9	0.9.- 0.7	0.7- 0.5	0.5-0.3	<0.3
**At least one common subtype (%)**	100	100	100	100	100
**Mean "length" 95CI subtype**	1.01	1.62	2.44	2.69	3.90

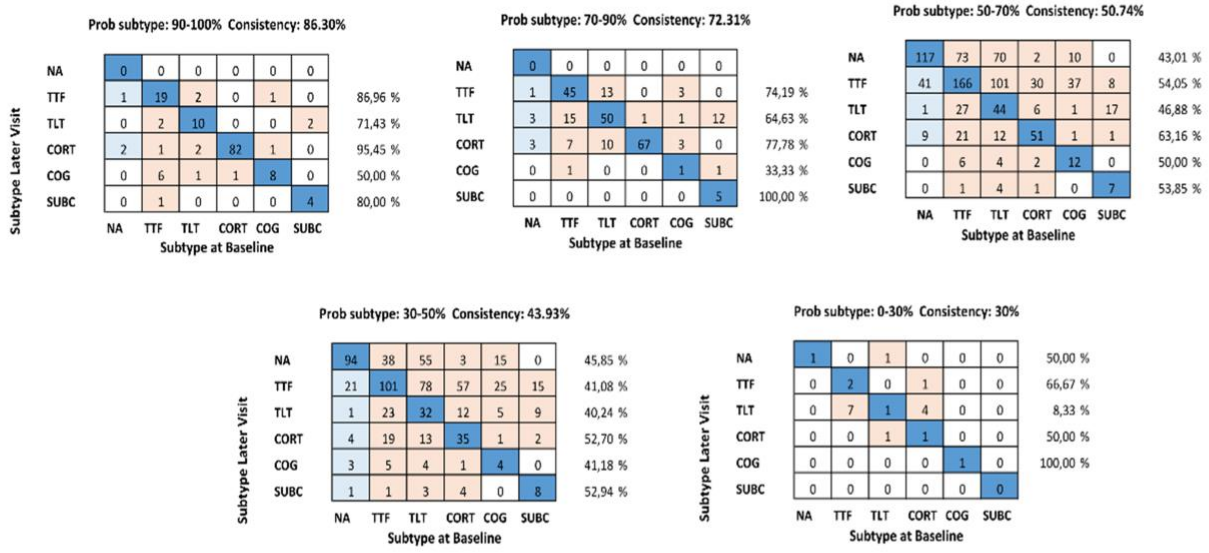

Supplementary Figure 5: Longitudinal subtype consistency stratified by the probability of the subtype assignment. A high probability of 90% or above gave a longitudinal consistency of 86.3%. This percentage decreased between 10 to 20 percent each time the probability of belonging to a subtype was lowered to 70, 50, 30 and <30. Abbreviations: Normal Appearing (NA); Typical Tau First (TTF); Typical Late Tau (TLT), Cortical (CORT); Cognitive (COG); Subcortical (SUBC).

Supplementary Table 3: Comparative Analysis of Subtypes Across Studied Variables. The table presents the subtypes compared, the associated p-value, and the relevant statistical test (either t-test or chi-square), depending on the nature of the variable under examination.

	Subtype "x"	Subtype "y"	P-value	statistic
**EDUCATION (t-test)**				
	Normal Appearing	Cognitive	0.005	2.816
	Typical Early Tau	Cortical	0.024	-2.253
	Typical Late Tau	Cognitive	0.015	2.478
	Cortical	Cognitive	0.002	3.152
**AGE (t-test)**				
	Normal Appearing	Typical Early Tau	<0.0001	-4.359
	Normal Appearing	Typical Late Tau	<0.0001	-4.687
	Normal Appearing	Cognitive	0.021	-2.347
	Normal Appearing	Subcortical	0.0002	-3.94
	Typical Early Tau	Cortical	0.0001	3.776
	Typical Late Tau	Cortical	<0.0001	4.105
	Cortical	Cognitive	0.047	-2.01
	Cortical	Subcortical	0.0006	-3.591
**TOTAL WMHV (t-test)**				
	Normal Appearing	Typical Late Tau	0.018	-2.417
**APOE - positive vs negative (Chi-Square)**				
	Normal Appearing	Typical Late Tau	<0.0001	21.29
	Normal Appearing	Typical Early Tau	<0.0001	21.27
	Typical Late Tau	Cortical	<0.0001	19.42
	Typical Late Tau	Subcortical	<0.0001	15.29
	Cortical	Typical Early Tau	<0.0001	21.49
	Typical Early Tau	Subcortical	<0.0001	16.14
	Subcortical	Cognitive	0.023	5.15
**AD diagnosis (Chi-Square)**				

	Normal Appearing	Typical Late Tau	<0.0001	15.95
	Normal Appearing	Cortical	0.02	5.35
	Normal Appearing	Typical Early Tau	<0.0001	28.2
	Normal Appearing	Subcortical	0.015	5.85
	Normal Appearing	Cognitive	<0.0001	21.25
	Typical Late Tau	Cortical	0.042	4.12
	Typical Late Tau	Typical Early Tau	0.047	3.92
	Cortical	Typical Early Tau	<0.0001	14.39
	Cortical	Cognitive	0.008	6.95
**Gender- Male (Chi-Square)**				
	Normal Appearing	Typical Late Tau	0.044	8.08
	Normal Appearing	Subcortical	0.03	4.29
	Normal Appearing	Cognitive	0.04	4.19
	Typical Late Tau	Typical Early Tau	0.0008	11.11
	Typical Early Tau	Subcortical	0.031	4.62
	Typical Early Tau	Cognitive	0.029	4.72
**Amyloid positivity (Chi-Square)**				
	Normal Appearing	Typical Late Tau	<0.0001	107.57
	Normal Appearing	Cortical	0.00289	8.87
	Normal Appearing	Typical Early Tau	<0.0001	110.82
	Normal Appearing	Cognitive	0.01247	6.24
	Typical Late Tau	Cortical	<0.0001	59.71
	Typical Late Tau	Subcortical	<0.0001	45.13
	Typical Late Tau	Cognitive	<0.0001	35.39
	Cortical	Typical Early Tau	<0.0001	60.62
	Typical Early Tau	Subcortical	<0.0001	44.1
	Typical Early Tau	Cognitive	<0.0001	34.45
**Memory Function Cognitive Battery (t-test)**				
	Normal Appearing	Typical Early Tau	<0.0001	12.83
	Normal Appearing	Typical Late Tau	<0.0001	10.61

	Normal Appearing	Cortical	<0.0001	7.68
	Normal Appearing	Cognitive	<0.0001	12.99
	Normal Appearing	Subcortical	<0.0001	6.16
	Typical Early Tau	Cortical	0.0002	-3.73
	Typical Early Tau	Cognitive	0.016	2.45
	Typical Late Tau	Cognitive	<0.0001	4.07
	Cortical	Cognitive	<0.0001	5.51
	Cognitive	Subcortical	0.001	-3.38
	Cognitive	Subcortical	0.001	-3.38
**Executive Function Cognitive Battery (t-test)**				
	Normal Appearing	Typical Early Tau	<0.0001	12.83
	Normal Appearing	Typical Late Tau	<0.0001	10.61
	Normal Appearing	Cortical	<0.0001	7.68
	Normal Appearing	Cognitive	<0.0001	12.99
	Normal Appearing	Subcortical	<0.0001	6.16
	Typical Early Tau	Cortical	0.000	-3.73
	Typical Early Tau	Cognitive	0.016	2.45
	Typical Late Tau	Cognitive	<0.0001	4.07
	Cortical	Cognitive	<0.0001	5.51
	Cognitive	Subcortical	0.0012	-3.38
	Cognitive	Subcortical	<0.0001	-6.53

REFERENCES

1. Salvatore C, Cerasa A, Castiglioni I. MRI Characterizes the Progressive Course of AD and Predicts Conversion to Alzheimer's Dementia 24 Months Before Probable Diagnosis. *Front Aging Neurosci*. 2018;10:135. doi:10.3389/fnagi.2018.00135

2. Mofrad SA, Lundervold AJ, Vik A, Lundervold AS. Cognitive and MRI trajectories for prediction of Alzheimer's disease. *Sci Rep*. 2021;11(1):2122. doi:10.1038/s41598-020-78095-7
