# Supplementary material for: Multimodal subtypes identified in Alzheimer’s Disease Neuroimaging Initiative participants by missing-data-enabled subtype and stage inference
Source: Brain Commun. 2024 Jun 25;6(4):fcae219. doi: 10.1093/braincomms/fcae219 (PMC11259979; doi:10.1093/braincomms/fcae219)
Supplement: fcae219_Supplementary_Data [file Original_submission_decision_letter.pdf .pdf › Original_submission_decision_letter.pdf .pdf]

Original Submission - Decision Letter

18-Jul-2023

Dear Ms. Estarellas Garcia,

Thank you for your recent submission BRAINCOM-2023-172 entitled "Multi-modal subtypes identified in ADNI by missing-data-enabled Subtype and Stage Inference" to Brain Communications.

Your manuscript has been reviewed, and while the reviewers and editorial team appreciate many aspects of the paper, they raise some concerns that you will need to address in order for the paper to be considered for publication.

If you can address these concerns, I invite you to submit a revised manuscript and a response to each of the reviewers' comments. The reviewer(s)' comments are included at the bottom of this message.

In addition to the reviewers' comments, please also address our Scientific Editor's comments added below to comply with our requirements for presentation of scientific data at Brain Communications.

Please format your revised manuscript according to our Instructions to Authors
https://academic.oup.com/braincomms/pages/General_Instructions
A summary of key points is attached to help with this. Please note that there can be substantial delays to publication if you don't resubmit in line with our requirements.

To revise your manuscript, click the link below or log into https://mc.manuscriptcentral.com/braincom and enter your Author Centre, where you will find your manuscript title listed under "Manuscripts with Decisions." Under "Actions," click on "Create a Revision." Your manuscript number will be appended to denote a revision.

*** PLEASE NOTE: This is a two-step process. After clicking on the link, you will be directed to a webpage to confirm. ***

https://mc.manuscriptcentral.com/braincom?URL_MASK=10a8094dcb174cbbba61a632ed3629e4

Please upload a revised manuscript with changes tracked with track changes or in coloured text.

When submitting your revised manuscript, you will be asked to respond to the reviewer(s) comments. Please be as specific as possible in your response to all of the comments.

The editorial team at Brain Communications aims to increase transparency in scientific publishing and to demonstrate the value that peer review brings to the scientific process. Please therefore consider whether you are willing to have reviewer(s)' comments on your paper and your response published as supplementary data with the paper if it is accepted. This is entirely optional and will only be done if both the authors and reviewers agree. While submitting your revision there will be an opportunity to review or update the answer you gave previously, if necessary.

IMPORTANT: Your original files are available to you when you upload your revised manuscript. Please delete any redundant files before completing the submission.

Because we are trying to facilitate timely publication of manuscripts submitted to Brain Communications, we would appreciate your revised manuscript as soon as possible. If it is not possible for you to resubmit within 3 months, the option to submit a revision will expire, and we would then consider your revised paper as a new submission. However, if you need a little more time, please get in touch and we can extend this deadline for you.

Once again, thank you for submitting your manuscript to Brain Communications and I look forward to receiving your revision.

Best wishes

Dr. Ian Simpson
Associate Editor, Brain Communications

Professor Tara Spires-Jones
Founding Editor, Brain Communications

Reviewer(s)' Comments to Author:

Reviewer: 1 (Konstantinos Poulakis)

Comments to the Author
Manuscript title: Multi-modal subtypes identified in ADNI by missing-data enabled Subtype and Stage Inference
Manuscript review:
In this study, the authors developed extended the SuStaIn model. Missing data imputation is incorporated. For comparison, the authors implemented two more commonly used methods of data imputation as well as a missing data removal strategy. Their model is applied to a very specific synthetic data design as well as to a publicly available dataset from ADNI. The results as well as the discussion show a good understanding of 1) the methodological challenges of unsupervised data modelling and 2) the Alzheimer's disease heterogeneity field.
After having read the manuscript, I attach here some considerations as well as comments and suggestions for improving the manuscript.

Page 6 out of 48
• From the text I understand that the model is fully explained in another publication, but please explain all sub/superscripts if they are used in the manuscript. It allows a better read of the model: k disease stage, N number of stages, P(t) a priori probability of individuals to belong to stage.. etc. Also, the order of the product (over J) and sum (over C) in equation 1 is changed compared to the original paper's formula. I see why, but either follow the exact order in the formula as in citation 3, or explain why the order is changed (this journal aims towards an applied Science readership).
• "Each event corresponds to the linear increase of a biomarker $i = 1 \ldots I$ to a z-score z{i1}… z{iRi}. Each biomarker trajectory is parameterised to start at start at $z = 0$ and end at $z = z$max."Does this imply that each biomarker's stage 0 and stage max must exist in the dataset (observed zs in the observed data space) or can they be inferred by extrapolation of the disease staging linear modelling? In other words (page 12 out of 47), is it so that you assume that all stages of a biomarker in a specific disease subtype should be included in your observed dataset? If yes, this should be discussed in the discussion's limitation section.

Page 7 out of 48
Can you present/build a measure (even empirical) for assessing the certainty over the classification of each subject to each subtype, according to the available information (non-missing data proportion)? This will help in understanding how much one should believe a subject's classification based on the amount of information available. Otherwise, the following statement "This strategy means that missing biomarker entries have no effect on the estimation of the subtypes and stages" also means that all subjects are classified with equal weights without knowing how much one should believe in their classification (for interpretational purposes).

Page 9 out of 48
On the synthetic dataset, do you have any simulation results on the effect of missing data imputation when missing data increase far more than 3% (30% or 40%)?

Page 11 out of 48
It is important and a methodologically beneficious that you have evaluated the missing data handling

strategies with more than one metrics. Why uniform data imputation versus knn works better (theoretically) can be discussed further.

Page 13 out of 48
Systematic data missing from a single modality (Benchmarking modalities). Can you discuss more how systematic data missing can be traced by the algorithm? For example, if CSF data are mostly missing from your biomarker sample, is there a way to assess how much the Sustain subtypes depend on the MRI, PET, CSF, or cognitive data?

Page 14 out of 48
The results presented here make sense in terms of ab positive and negative sub-analyses, since the model is applied in different datasets and discrimination rules change accordingly.

Page 18 out of 48
Can this be true based on the literature in AD subtypes? "In all datasets 'Cognitive' showed the fastest progression, followed by 'Typical Late Tau' and 'Typical Early Tau', which have a very similar rate of progression; then 'Cortical', and finally 'Subcortical' showed the slowest MCI to AD progression." Discuss or investigate and discuss this result more (e.g. Early versus late onset cortical atrophy in AD).

Page 22 out of 48
I understand that this is a methodological study, but since it is submitted in BrainComm can you please discuss your results with respect to Alzheimer's disease staging more? For example, cortical AD (some cases may be posterior cortical atrophy patients) is more aggressive in its cognitive deterioration etc. You mention: "As the 'Cortical' subtype had the highest educational level, a slow rate of progression from MCI to AD and the best memory function, the 'Cortical' group may have a higher cognitive reserve and therefore a higher tolerance to pathology". How about the cognitive profiles of hippocampal sparing AD patietns after being diagnosed with AD?

Page 23 out of 48
Limitations: How about low versus high dimensional dataset utilization for disease heterogeneity assessment? This model utilizes 17 biomarkers (based on figure S1) that present the summary of a very large set of features (MRI, PET, CSF, cognition). Is this a potential limitation of the overall modelling approach? Can variable importance (for easier interpretation) be incorporated in SuStaIn?

Reviewer: 2

Comments to the Author
The manuscript _Multi-modal subtypes identified in ADNI by missing-data-enabled Subtype and Stage Inference_ of Esterrelas et al. introduces an extension of the statistical model of Young et al. (SuStaIn) to allow modelling datasets that comprise missing data. The authors validate the method on a simulated dataset and apply it to a selection of subjects from the ADNI database.

Handling missing data is especially useful to combine modalities. Published studies that leverage the Subtyping and Staging Inference method mainly focus on multi-featured progression models derived from a single modality (e.g. modelling the progression of cortical volumes, all acquired & derived from the same MRI session). Here the authors showcase how this can be applied to discover subtypes comprising both structural atrophy, PET & CSF measurements of amyloid and tau proteins, as well as patient's cognition. This is a very welcome endeavour in the field of subtyping in disease progression modelling.

Multiple strategies to handle missing data are presented and evaluated in a seemingly realistic simulated dataset (in which missing data seem to follow missing proportions observed in the real data). One of them is novel and specific to the SuStaIn framework, while the three other ones are more common imputation approaches. The authors benchmark these four strategies on the simulated dataset and based on both accuracy at the level of the discovered subtype (through Kendal's tau) and at the individual level (both in staging and assigned subtype) were considered. The author's proposed method (the so-called Uniform) was selected and used for further analysis of ADNI data. Five subtypes are

discovered on ADNI and appear consistent with the literature, as well as providing interesting predictive utility, especially for predicting MCI / AD conversion, as well as a good longitudinal consistency.

Overall, the manuscript is well written and combines an interesting methodological increment to an established and robust statistical framework with a sound analysis of real data from an AD cohort. It addresses both the question of the reliability and stability of the method when run in different conditions (missing modalities, different subpopulations, etc) as well as the clinical relevance of the discovered subtypes (in terms of predictive ability for conversion, consistency with the published subtyping literature of AD, correlation with external clinical covariates and risk factors).

Here are some points that could benefit from some clarification:

1. Page 7, section about MD1: it is a bit unclear to me how the modelling assumptions behind the unobserved x_{ij} being assumed uniform over its range (p7 L23) fits into the staging component of SuStaIn: I'm especially not sure how this translates to a uniform distribution for the probabilities conditioned by the stage $P(x|t)$.

More precisely setting the value of the conditional probability $P(x_{ij} \mid t)$ to $1/z_i^{max}$ when x_{ij} is unobserved seems to ignore that, conditioned by t, the range $[0, z_i^{max}]$ might not endowed anymore with a uniform distribution over the biomarker values — because observed values are expected to follow a piecewise trajectory whose level depends on the stage. For example: if a biomarker i is such that all its abnormality stages are only attained at the very end of the progression (i.e. $g_i(t)$ stays at zero for almost all the progressions except for the last few stages). Then for a time that corresponds to an early stage — say $t=1/(N+1))$ — and if an unobserved x is treated as uniform — $x \sim U(0, z_i^{max})$ — I don't see why $P(x \mid t)$ should be approximated by $1/z_i^{max}$ while in fact, a uniform x has a high probability of having quite a low conditional $P(x|t)$ under $N(g_i(t), s_i)$. This is because the "most common observation for this biomarker" is in fact a low abnormality (and not a uniform one). Why isn't $P(x \mid t)$ therefore modeled as $E_{x\sim U(0,z_i^{max})} [N(x, g_i(t), s_i)]$ when x is unobserved?

Maybe the choice of $1/z_i^{max}$ still makes sense (and it does look valuable from the experimental study on both simulation and real data), but it does not seem to be a consequence of assuming that unobserved values would have been drawn from a uniform distribution over their range as stated on page 7 L23. I think this section, which is part of the methodological contribution of the manuscript would benefit from a justification/clarification/rewording.

2. Page 8 line 16: inconsistency in the notation of x_{ij} (the sum over the subjects is computed over i, which differs from the notation introduced earlier on page 6 (l.21))
3. Page 8 lines 34 – 36: for the first feature vector, it is unclear to me over what exactly are the mean / range and std of a biomarker computed for each single patient
4. Figure 2. The caption should be "Sankey" and not "San Key". The readability of the figure could benefit from a better ordering of both the "in" and the "out" of the flow, such that it prevents "artificial" crossings (e.g. on the A panel: "Normal Appearing" or "Typical Late Tau" could appear at the same rank on both sides).

Comments from the Scientific Editor:

-Please supply a graphical abstract. A graphical abstract is a visual representation of the central finding or methodology of the paper. It will be displayed in the table of contents. Authors should strive to make the graphical abstract informative, interesting, visually appealing, and straightforward (no caption needed, no abbreviations except gene names). The technical requirements for the graphical abstracts are:
- Size: 120 x 120 mm (300-600dpi) or 3000 x 3000 pixels
- Font: Arial 12-16 pt
- File type: tiff or PDF
- Aspect ratio: 1:1 preferred

-Please add an Abbreviated Summary (up to 50 words) of your paper that captures the main purpose and conclusions of the work. The summary should state the main results written in the third person (‘[1st author surname] et al. report that …’) and conclusion. This summary will be used in the contents list online and should not contain any abbreviations other than those on the accepted abbreviations list and accepted gene/protein names, making it accessible to the non-specialist. Please upload it as a Word document. Allowed abbreviations: AIDS; ANOVA; AMPA; ATP; A,T,C,G; CNS; CSF; CT; DNA; ECG; EEG; EMG; GABA; HIV; MRI; NMDA; PET; PCR; RNA.

-Please include a paragraph about statistical analysis in the 'Materials and Methods' section.

- Figure 3a,b,g: if you are comparing more than 2 data-groups, you may want to revisit your choice of statistical analysis.

- please, make sure that all the abbreviations in figures are spelled out either in the figure or in the legend.

-for each statistical analysis part of a figure, please report in the figure legend the test used and relevant test statistic values alongside p values (for example F or t values for ANOVAs and t-tests).
