# Supplementary material for: Multimodal subtypes identified in Alzheimer’s Disease Neuroimaging Initiative participants by missing-data-enabled subtype and stage inference
Source: Brain Commun. 2024 Jun 25;6(4):fcae219. doi: 10.1093/braincomms/fcae219 (PMC11259979; doi:10.1093/braincomms/fcae219)
Supplement: fcae219_Supplementary_Data [file Original_submission_Response_to_reviewers_comments.pdf .pdf › Original_submission_Response_to_reviewers_comments.pdf .pdf]

We thank the reviewers for their detailed feedback on the manuscript. Addressing their concerns has substantially improved the readability of the manuscript for a general audience and strengthened the validity of the results.

Reviewer(s)' Comments to Author:

Reviewer: 1

Comments to the Author
Manuscript title: Multi-modal subtypes identified in ADNI by missing-data enabled Subtype and Stage Inference
Manuscript review:
In this study, the authors developed extended the SuStaIn model. Missing data imputation is incorporated. For comparison, the authors implemented two more commonly used methods of data imputation as well as a missing data removal strategy. Their model is applied to a very specific synthetic data design as well as to a publicly available dataset from ADNI. The results as well as the discussion show a good understanding of 1) the methodological challenges of unsupervised data modelling and 2) the Alzheimer's disease heterogeneity field.
After having read the manuscript, I attach here some considerations as well as comments and suggestions for improving the manuscript.

Page 6 out of 48
• From the text I understand that the model is fully explained in another publication, but please explain all sub/superscripts if they are used in the manuscript. It allows a better read of the model: k disease stage, N number of stages, P(t) a priori probability of individuals to belong to stage.. etc.

R1.1 Thank you for spotting this; we have added this missing information about sub/superscripts (see page 6)

Also, the order of the product (over J) and sum (over C) in equation 1 is changed compared to the original paper's formula. I see why, but either follow the exact order in the formula as in citation 3, or explain why the order is changed (this journal aims towards an applied Science readership).

R1.2 This is an important observation. On further inspection, the order of the product and sum are correct here, but incorrect in citation 3. This doesn't affect the results in citation 3 as the order is correct in the programming code (https://github.com/ucl-pond). We will handle this by submitting a correction for the formula in citation 3, rather than propagating the error to this work.

• "Each event corresponds to the linear increase of a biomarker $i = 1$ to a z-score z{i1}… z{iRi}. Each biomarker trajectory is parameterised to start at $z = 0$ and end at $z = z$max." Does this imply that each biomarker's stage 0 and stage max must exist in the dataset (observed zs in the observed data space) or can they be inferred by extrapolation of the disease staging linear modelling? In other words (page 12 out of 47), is it so that you assume that all stages of a biomarker in a specific disease subtype should be included in your observed dataset? If yes, this should be discussed in the discussion's limitation section.

R1.3 Thanks for this question. The z-scores at stage 0 and stage max are indeed assumed to exist in the observed data space and are chosen as follows (see page 12):

The minimum biomarker z score was set to zero for all biomarkers, while the maximum was set to the rounded 95th percentile computed for each biomarker.

It is possible that specific combinations of z-scores across markers may be under-represented in the dataset, this will manifest as uncertainty in the positional variance diagrams. We have now mentioned this in the limitations section (see page 25):

SuStaIn requires that a set of z-scores are specified for each marker; we chose the z-scores to be reflective of the range of values for each marker. However, specific combinations of z-scores across markers may be under-represented in a particular dataset, manifesting as uncertainty in the positional variance diagrams.

Page 7 out of 48
Can you present/build a measure (even empirical) for assessing the certainty over the classification of each subject to each subtype, according to the available information (non-missing data proportion)?. This will help in understanding how much one should believe a subject's classification based on the amount of information available. Otherwise, the following statement "This strategy means that missing biomarker entries have no effect on the estimation of the subtypes and stages" also means that all subjects are classified with equal weights without knowing how much one should believe in their classification (for interpretational purposes).

R1.4 A measure for assessing the probably of a subject belonging to a certain subtype and stage is already built into the model. Figure S5 details the longitudinal consistency of the subtype assignments based on the confidence in the subtype assignment, demonstrating that individuals with a lower probability of belonging to a particular subtype at baseline are less likely to be assigned to the same subtype at follow-up.

We have further added Table S2, which shows that the 95% confidence intervals for the subtype assignment at baseline overlap with the 95% confidence intervals for the subtype assignment at follow-up, i.e. there is a single consistent subtype at baseline and follow-up that an individual could be assigned to. The width of this confidence interval increases for individuals with less certain subtype assignments. These findings together suggest that the subtype probability and the confidence interval of the subtypes are a useful reflection of the uncertainty in the subtype assignment.

Thank you for spotting the ambiguity in the statement you highlighted. We intended the statement to mean that missing biomarker entries don't affect the overall progression pattern. Missing data for an individual does affect the confidence in their subtype assignment. We have now clarified this statement to:

*"This strategy means that missing biomarker entries have no **effect on the overall progression pattern**."*

Page 9 out of 48
On the synthetic dataset, do you have any simulation results on the effect of missing data imputation when missing data increase far more than 3% (30% or 40%)?

R1.5 Thanks for this suggestion, we have now added results with 40% missing data to Table 1. The performance decreases slightly (percentage of correct subtype assignments decreases from 85% to 75% and the error in the stage increases from 1.5 stages to 2.2 stages), but our proposed uniform distribution approach still clearly out-performs alternative approaches.

Missing Data	Progression pattern similarity<br>Mean Kendall Tau Distance	Subtype<br>% correct	Stage<br>Mean diff (stand dev)
Deletion	-	-	-
Mean	0.69	68.80	2.39 (3.39)
KNN Biomarkers (feature matrix)	0.68	46.80	2.44 (2.18)
Uniform distribution	0.86	75.20	2.22 (2.06)
Full Data	0.923	85.80	1.54 (1.31)

Page 11 out of 48
It is important and a methodologically beneficious that you have evaluated the missing data handling strategies with more than one metrics. Why uniform data imputation versus knn works better (theoretically) can be discussed further.
R1.6 Thanks for this comment, we have added the following to the Discussion (page 20):

“Missing-data SuStaIn may be less biased than imputation approaches such as KNN because it doesn’t make any assumptions about the values of the missing biomarkers. Rather than imputing a value when a biomarker is missing, missing-data SuStaIn simply encodes that there is no information available for that biomarker (by using a probability distribution to indicate that any biomarker value is equally likely). This feature allows missing-data SuStaIn to appropriately handle uncertainty, estimating subtype progression patterns and performing subtyping and staging based only on the recorded data entries.”

Page 13 out of 48
Systematic data missing from a single modality (Benchmarking modalities). Can you discuss more how systematic data missing can be traced by the algorithm? For example, if CSF data are mostly missing from your biomarker sample, is there a way to assess how much the Sustain subtypes depend on the MRI, PET, CSF, or cognitive data?
R1.7 In Supplementary Material S4, we show the significance of various modalities in subtyping and staging, emphasizing the importance of multimodal data in our study. We investigated the subtyping and staging of subjects at baseline by comparing results using the complete set of biomarkers against those where one modality was removed. Given that CSF and PET both reflect similar pathological processes, we also explored the effects of excluding them concurrently. As evidenced by Figure S4 and Table S1, CSF and PET data seem to be pivotal features for subtyping, likely because they represent the only early AD-specific markers included in our analysis. On the other hand, MRI proved to be the most significant biomarker for staging; its absence typically results in individuals being categorized into higher SuStaIn stages. This aligns with our expectation that MRI most accurately captures the progressive changes spanning the disease's trajectory.

Page 14 out of 48
The results presented here make sense in terms of ab positive and negative sub-analyses, since the model is applied in different datasets and discrimination rules change accordingly.
R1.8 Thanks for this comment. We added the sub-analyses to shed light on which subtypes may be more likely to be Alzheimer’s subtypes vs. non-Alzheimer’s subtypes. We agree this has the added benefit of providing insight into how the model might translate to different datasets.

Page 18 out of 48
Can this be true based on the literature in AD subtypes? “In all datasets ‘Cognitive’ showed the

fastest progression, followed by ‘Typical Late Tau’ and ‘Typical Early Tau’, which have a very similar rate of progression; then ‘Cortical’, and finally ‘Subcortical’ showed the slowest MCI to AD progression.” Discuss or investigate and discuss this result more (e.g. Early versus late onset cortical atrophy in AD).
R1.9 Thanks for this interesting point. We have revised the term ‘datasets’ to ‘data subsets’ for clarity.

Regarding the literature on Alzheimer’s disease subtypes, we believe our subtypes, and in particular the cortical subtype the reviewer mentions, broadly align with the literature. The systematic review of Ferreira et al. Neurology 2020 on Alzheimer’s disease subtypes identified that individuals with hippocampal-sparing Alzheimer’s disease were younger, male, APOE4 noncarriers, had higher cognitive reserve, and a non-amnestic syndrome. Our cortical subtype is younger, has a higher proportion of men than the ‘normal appearing’ subtype, are predominantly APOE4 noncarriers, have higher years of education, and perform slightly worse on executive function than memory function (although both functions are relatively preserved – see points 2 and 3 below).

The key difference between our cortical subtype and the characteristics identified in Ferreira et al. Neurology is that our cortical subtype does not have higher CSF p-tau and t-tau. We believe this is likely because our cortical subtype includes both amyloid-positive and amyloid-negative individuals (see point 3 below), and thus does not only reflect hippocampal-sparing Alzheimer’s disease, but also other non-Alzheimer’s disease related changes.

The review of Ferreira et al. highlights that the literature on the rate of progression of individuals with a cortical Alzheimer’s disease subtype is inconclusive: “Cognitive trajectories over time more clearly differ across subtypes, highlighting heterogeneity in disease progression as well. Longitudinal decline in MMSE was faster in hippocampal-sparing than in other subtypes.[9,10,23,29] Na et al.[29] showed that this funding extends to several memory and nonmemory cognitive domains that also decline faster in hippocampal-sparing AD. However, other studies showed that typical AD has the fastest longitudinal decline in MMSE.[16,18] The results on longitudinal decline in CDR are similar than those for MMSE, that is, faster decline is reported for typical AD in a study,[16] whereas faster decline is reported for hippocampal-sparing AD in other studies.[18,29] These contradictory results may depend on differences across cohorts in the frequency of patients with early-onset non-amnestic AD presentations.” Their references are:
[9] Murray Lancet Neurol 2011. Neuropathologically defined subtypes of Alzheimer’s disease with distinct clinical characteristics: a retrospective study.
[10] Janocko Acta Neuropathol 2012. Neuropathologically defined subtypes of Alzheimer’s disease differ significantly from neurofibrillary tangle-predominant dementia.
[16] Ferreira Sci Rep 2017. Distinct subtypes of Alzheimer’s disease based on patterns of brain atrophy: longitudinal trajectories and clinical applications.
[18] Persson Plos One 2017. MRI-assessed atrophy subtypes in Alzheimer’s disease and the cognitive reserve hypothesis.
[23] Byun Plos One 2015. Heterogeneity of regional brain atrophy patterns associated with distinct progression rates in Alzheimer’s disease.
[29] Na Neurobiol Aging 2016. Malignant progression in parietal-dominant atrophy subtype of Alzheimer’s disease occurs independent of onset age.

Our finding that the typical subtype has faster conversion from MCI to AD than the cortical subtype is consistent with the subset of these studies that report faster decline in typical Alzheimer’s disease than hippocampal-sparing Alzheimer’s disease.

However, we also note that our subtypes would not be expected to align exactly with those in the literature for several reasons:

1. The subtypes are dynamic, i.e. they have a temporal component such that the subtype profile evolves over time. We have labelled them according to their earliest feature to change, however individuals at later stages in the subtypes will have abnormalities across a broader set of biomarkers. We have added a comment to highlight this point:

   We labelled these five different subtypes as: 'Typical AD Early Tau', 'Typical AD Late Tau', 'Cortical', 'Cognitive' and 'Subcortical' ***according to early characteristic features of each progression pattern, however we note that the subtypes will have different profiles at different stages as they model temporal progression****.*

2. Our subtypes capture individuals ranging from cognitively normal to mild cognitive impairment to Alzheimer's disease, whereas much of the literature on Alzheimer's subtypes focusses only on individuals with Alzheimer's disease.

3. Our subtypes include both amyloid-positive and amyloid-negative individuals. Table 2 highlights that individuals assigned to the cortical, cognitive, and subcortical subtypes had lower rates of amyloid positivity (40.5%, 41.2% and 27.3% respectively in those with amyloid information available) than the typical early and late tau subtypes (82.3% and 84.9% respectively in those with amyloid information available).

We thank the reviewer again for this interesting discussion point and have added the following text to the discussion (page 22) to highlight these points:

The 'Cortical' subtype had a lower proportion of individuals that were amyloid positive (40.5% compared to 82.3% and 84.9% for the two 'Typical' subtypes) and with an AD diagnosis (7.8% compared to 23.8% and 15.8% for the 'Typical' subtypes), suggesting that this subtype reflects a heterogeneous group of individuals with cortical atrophy, some of whom may have AD or pre-symptomatic AD, but others who may have non-AD related changes.

The literature on the rate of decline of cognition in AD subtypes is inconclusive [ref Ferreira Neurology 2020], with some studies suggesting that typical AD has the fastest rates of decline [ref Ferrerira Sci Rep 2017; Persson Plos One 2017], and others suggesting that hippocampal-sparing AD has the fastest rates of decline [re Murray Lancet Neurol 2011; Janocko Acta Neuropathol 2012; Byun Plos One 2015; Na Neurobiol Aging 2016]. Our study suggests that typical AD has the fastest rate of decline, however the 'Cortical' subtype combines individuals across a range of disease stages and includes a high proportion of individuals who are amyloid negative. It is possible that the rate of decline is faster in individuals who are amyloid positive.

Page 22 out of 48
I understand that this is a methodological study, but since it is submitted in BrainComm can you please discuss your results with respect to Alzheimer's disease staging more? For example, cortical AD (some cases may be posterior cortical atrophy patients) is more aggressive in its cognitive deterioration etc. You mention: "As the 'Cortical' subtype had the highest educational level, a slow rate of progression from MCI to AD and the best memory function, the 'Cortical' group may have a higher cognitive reserve and therefore a higher tolerance to pathology". How about the cognitive profiles of hippocampal sparing AD patients after being diagnosed with AD?

R1.10 Thanks for this interesting discussion point. See the previous response (R1.9), which discusses this.

Page 23 out of 48
Limitations: How about low versus high dimensional dataset utilization for disease heterogeneity assessment? This model utilizes 17 biomarkers (based on figure S1) that present the summary of a very large set of features (MRI, PET, CSF, cognition). Is this a potential limitation of the overall modelling approach? Can variable importance (for easier interpretation) be incorporated in SuStaIn?

R1.11 Thank you for this point, we have now added a comment on this in the limitation section, page 25:

SuStaIn currently handles relatively small sets of features only. Leveraging a more comprehensive dataset could offer a richer picture, particularly in combination with feature selection strategies to determine the most informative biomarkers or features. Such an approach could balance computational efficiency and information richness.

Reviewer: 2

Comments to the Author
The manuscript _Multi-modal subtypes identified in ADNI by missing-data-enabled Subtype and Stage Inference_ of Esterrelas et al. introduces an extension of the statistical model of Young et al. (SuStaIn) to allow modelling datasets that comprise missing data. The authors validate the method on a simulated dataset and apply it to a selection of subjects from the ADNI database.

Handling missing data is especially useful to combine modalities. Published studies that leverage the Subtyping and Staging Inference method mainly focus on multi-featured progression models derived from a single modality (e.g. modelling the progression of cortical volumes, all acquired & derived from the same MRI session). Here the authors showcase how this can be applied to discover subtypes comprising both structural atrophy, PET & CSF measurements of amyloid and tau proteins, as well as patient's cognition. This is a very welcome endeavour in the field of subtyping in disease progression modelling.

Multiple strategies to handle missing data are presented and evaluated in a seemingly realistic simulated dataset (in which missing data seem to follow missing proportions observed in the real data). One of them is novel and specific to the SuStaIn framework, while the three other ones are more common imputation approaches. The authors benchmark these four strategies on the simulated dataset and based on both accuracy at the level of the discovered subtype (through Kendal's tau) and at the individual level (both in staging and assigned subtype) were considered. The author's proposed method (the so-called Uniform) was selected and used for further analysis of ADNI data. Five subtypes are discovered on ADNI and appear consistent with the literature, as well as providing interesting predictive utility, especially for predicting MCI / AD conversion, as well as a good longitudinal consistency.

Overall, the manuscript is well written and combines an interesting methodological increment to an established and robust statistical framework with a sound analysis of real data from an AD cohort. It addresses both the question of the reliability and stability of the method when run in different conditions (missing modalities, different subpopulations, etc) as well as the clinical relevance of the

discovered subtypes (in terms of predictive ability for conversion, consistency with the published subtyping literature of AD, correlation with external clinical covariates and risk factors).

Here are some points that could benefit from some clarification:

1. Page 7, section about MD1: it is a bit unclear to me how the modelling assumptions behind the unobserved x_{ij} being assumed uniform over its range (p7 L23) fits into the staging component of SuStaIn: I'm especially not sure how this translates to a uniform distribution for the probabilities conditioned by the stage P(x|t).

More precisely setting the value of the conditional probability P(x_{ij} | t) to 1/z_i^{max} when x_{ij} is unobserved seems to ignore that, conditioned by t, the range [0, z_i^{max}] might not endowed anymore with a uniform distribution over the biomarker values — because observed values are expected to follow a piecewise trajectory whose level depends on the stage. For example: if a biomarker i is such that all its abnormality stages are only attained at the very end of the progression (i.e. g_i(t) stays at zero for almost all the progressions except for the last few stages). Then for a time that corresponds to an early stage — say t=1/(N+1)) — and if an unobserved x is treated as uniform — x ~ U(0, z_i^{max}) — I don't see why P(x | t) should be approximated by 1/z_i^{max} while in fact, a uniform x has a high probability of having quite a low conditional P(x|t) under N(g_i(t), s_i). This is because the "most common observation for this biomarker" is in fact a low abnormality (and not a uniform one).
Why isn't P(x | t) therefore modeled as E_{x~U(0,z_i^{max}} [N(x, g_i(t), s_i)] when x is unobserved?

Maybe the choice of 1/z_i^{max} still makes sense (and it does look valuable from the experimental study on both simulation and real data), but it does not seem to be a consequence of assuming that unobserved values would have been drawn from a uniform distribution over their range as stated on page 7 L23. I think this section, which is part of the methodological contribution of the manuscript would benefit from a justification/clarification/rewording.

Thanks for this interesting and thoughtful comment on the choice of assuming a uniform probability over the range of the data. This design choice arose from translating the method for handling missing data used by the event-based model to the z-score model. In the event-based model (Fonteijn et al. Neuroimage, 2012), missing data is handled by assuming that P(x|E) = P(x|not E), i.e. if data is missing for a biomarker it is equally likely that that particular biomarker event has or has not occurred for that individual. Here we have a set of z-score events, rather than just normal vs abnormal events. In this case we wish to express that it is equally likely that the data could come from any z-score event (similarly to assuming the data could equally come from either the biomarker event has or hasn't occurred distribution) and so we chose a uniform distribution over the range of z-scores. Your alternative suggestion is very interesting but induces a dependency of the modelled missing data likelihood on the ordering of events, for which there is no such assumption made in the event-based model, so we choose to keep our current formulation, which works well on simulated data.

2. Page 8 line 16: inconsistency in the notation of x_ij (the sum over the subjects is computed over i, which differs from the notation introduced earlier on page 6 (l.21))
R2.2 Thanks for spotting this, we have changed the notation to $x_{ij} = \frac{1}{J}\sum_{i=1}^{J} x_{ij}$ (where x_ij is the measurement of biomarker i in subject j, j = 1 … J.) in page 8.

3. Page 8 lines 34 – 36: for the first feature vector, it is unclear to me over what exactly are the mean / range and std of a biomarker computed for each single patient

R2. 3 To determine how similar subjects are to one another, KNN uses a distance metric. The 'distance' between subjects, in this case is not calculated based on just one feature or attribute but using feature vectors. In this context, there are two types of feature vectors being used. In one of them, for each subject, three statistical measures are calculated for their biomarker values: Mean, Range and Standard Deviation.

The following clarification has been added to the manuscript (page 8):

Since the biomarkers can vary significantly in terms of magnitude, using this feature vector ensures that the algorithm can account for both the overall statistical distribution (mean, range, standard deviation). Now, each subject is represented by three dimensions. Using these dimensions, we can compute the distance between subjects. After establishing the distance metrics, we refer to the original matrix to inspect the actual biomarker values for the subject with the missing data. We then impute this missing value by calculating the mean of these observed biomarker values.

4. Figure 2. The caption should be "Sankey" and not "San Key". The readability of the figure could benefit from a better ordering of both the "in" and the "out" of the flow, such that it prevents "artificial" crossings (e.g. on the A panel: "Normal Appearing" or "Typical Late Tau" could appear at the same rank on both sides).

R2.4 Thanks or spotting this, we have changed the caption to Sankey and altered the ordering of the subtypes in the figures. The figures are now as follows:

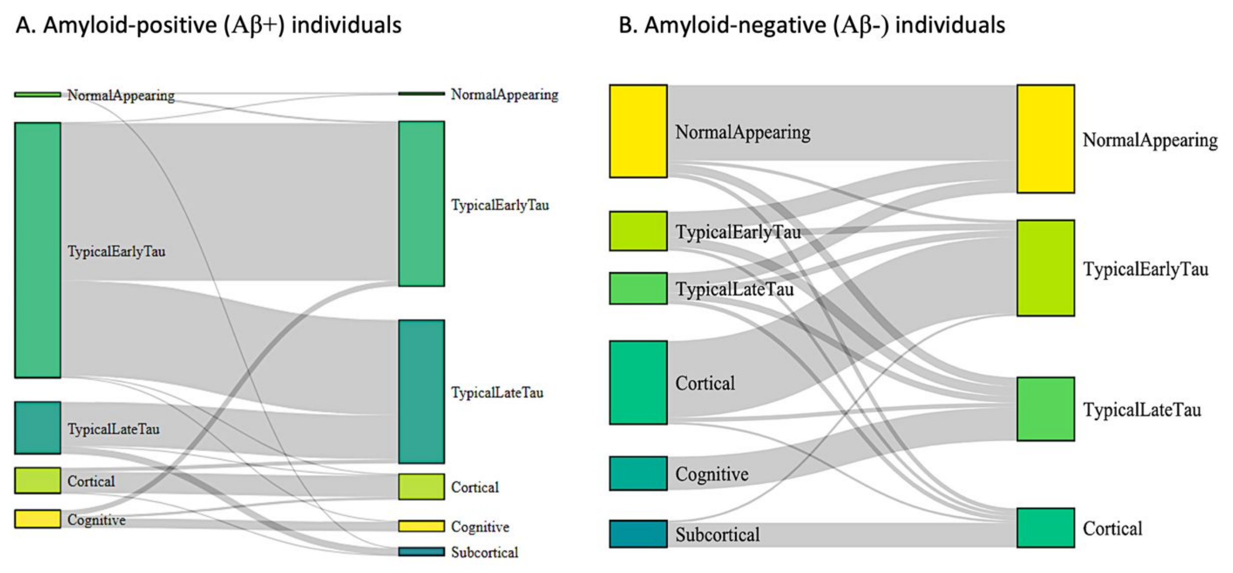

Figure 2 Two Sankey flows showing subtyping consistency between models. A Normal Appearing subtype was included, consisting of those subjects assigned to Stage 0. Diagram A compares the 406 Aβ+ subjects, subtyped by the Aβ+ (left) and full data model (right). Diagram B compares the 275 Aβ- subjects, subtyped by the Aβ- (right) and full data model (left).

Comments from the Scientific Editor:

-Please supply a graphical abstract. A graphical abstract is a visual representation of the central finding or methodology of the paper. It will be displayed in the table of contents. Authors should

strive to make the graphical abstract informative, interesting, visually appealing, and straightforward (no caption needed, no abbreviations except gene names). The technical requirements for the graphical abstracts are:
- Size: 120 x 120 mm (300-600dpi) or 3000 x 3000 pixels
- Font: Arial 12-16 pt
- File type: tiff or PDF
- Aspect ratio: 1:1 preferred

SE1. Please see attached in PDF form the Graphical Abstract. Thank you.

-Please add an Abbreviated Summary (up to 50 words) of your paper that captures the main purpose and conclusions of the work. The summary should state the main results written in the third person ('[1st author surname] et al. report that …') and conclusion. This summary will be used in the contents list online and should not contain any abbreviations other than those on the accepted abbreviations list and accepted gene/protein names, making it accessible to the non-specialist. Please upload it as a Word document. Allowed abbreviations: AIDS; ANOVA; AMPA; ATP; A,T,C,G; CNS; CSF; CT; DNA; ECG; EEG; EMG; GABA; HIV; MRI; NMDA; PET; PCR; RNA.

SE2. "Estarellas et al. report that an adapted Subtype and Stage Inference algorithm accommodates missing data in multimodal Alzheimer's Disease studies. They identified five distinct subtypes, each associated with different risk factors, revealing new insights into the disease's landscape and progression."

- Please include a paragraph about statistical analysis in the 'Materials and Methods' section.

SE3. The section 'Experiment' refers to the Statistical analysis. Section title has been changed for clarification (page 11).

- Figure 3a,b,g: if you are comparing more than 2 data-groups, you may want to revisit your choice of statistical analysis.

SE4. We are performing pairwise comparison between subtypes.

- please, make sure that all the abbreviations in figures are spelled out either in the figure or in the legend.

SE5. Thank you for your observations. Abbreviations have been added to all figures.

-for each statistical analysis part of a figure, please report in the figure legend the test used and relevant test statistic values alongside p values (for example F or t values for ANOVAs and t-tests).
SE6. Thank you for your valuable feedback. To address this comment, we have prepared a supplementary table that provides all the requested statistical details. We opted for this approach to maintain the clarity of the figure legends, given the volume of the information. We trust this solution effectively presents the necessary data. Kindly refer to the supplementary table S3 (page 7 Supplementary Material) attached for detailed statistics.

	Subtype	Subtype 2	P-value	statistic
**EDUCATION (t-test)**				
	Normal Appearing	Cognitive	0.005	2.816
	Typical Early Tau	Cortical	0.024	-2.253
	Typical Late Tau	Cognitive	0.015	2.478
	Cortical	Cognitive	0.002	3.152
**AGE (t-test)**				
	Normal Appearing	Typical Early Tau	<0.0001	-4.359
	Normal Appearing	Typical Late Tau	<0.0001	-4.687
	Normal Appearing	Cognitive	0.021	-2.347
	Normal Appearing	Subcortical	0.0002	-3.94
	Typical Early Tau	Cortical	0.0001	3.776
	Typical Late Tau	Cortical	<0.0001	4.105
	Cortical	Cognitive	0.047	-2.01
	Cortical	Subcortical	0.0006	-3.591
**TOTAL WMHV (t-test)**				
	Normal Appearing	Typical Late Tau	0.018	-2.417
**APOE - positive vs negative (Chi-Square)**				
	Normal Appearing	Typical Late Tau	<0.0001	21.29
	Normal Appearing	Typical Early Tau	<0.0001	21.27

	Typical Late Tau	Cortical	<0.0001	19.42
	Typical Late Tau	Subcortical	<0.0001	15.29
	Cortical	Typical Early Tau	<0.0001	21.49
	Typical Early Tau	Subcortical	<0.0001	16.14
	Subcortical	Cognitive	0.023	5.15
**AD diagnosis (Chi-Square)**				
	Normal Appearing	Typical Late Tau	<0.0001	15.95
	Normal Appearing	Cortical	0.02	5.35
	Normal Appearing	Typical Early Tau	<0.0001	28.2
	Normal Appearing	Subcortical	0.015	5.85
	Normal Appearing	Cognitive	<0.0001	21.25
	Typical Late Tau	Cortical	0.042	4.12
	Typical Late Tau	Typical Early Tau	0.047	3.92
	Cortical	Typical Early Tau	<0.0001	14.39
	Cortical	Cognitive	0.008	6.95
**Gender- Male (Chi-Square)**				
	Normal Appearing	Typical Late Tau	0.044	8.08
	Normal Appearing	Subcortical	0.03	4.29
	Normal Appearing	Cognitive	0.04	4.19
	Typical Late Tau	Typical Early Tau	0.0008	11.11
	Typical Early Tau	Subcortical	0.031	4.62
	Typical Early Tau	Cognitive	0.029	4.72
**Amyloid positivity (Chi-Square)**				
	Normal Appearing	Typical Late Tau	<0.0001	107.57
	Normal Appearing	Cortical	0.00289	8.87
	Normal Appearing	Typical Early Tau	<0.0001	110.82
	Normal Appearing	Cognitive	0.01247	6.24
	Typical Late Tau	Cortical	<0.0001	59.71
	Typical Late Tau	Subcortical	<0.0001	45.13
	Typical Late Tau	Cognitive	<0.0001	35.39

	Cortical	Typical Early Tau	<0.0001	60.62
	Typical Early Tau	Subcortical	<0.0001	44.1
	Typical Early Tau	Cognitive	<0.0001	34.45
**Memory Function Cognitive Battery (t-test)**				
	Normal Appearing	Typical Early Tau	<0.0001	12.83
	Normal Appearing	Typical Late Tau	<0.0001	10.61
	Normal Appearing	Cortical	<0.0001	7.68
	Normal Appearing	Cognitive	<0.0001	12.99
	Normal Appearing	Subcortical	<0.0001	6.16
	Typical Early Tau	Cortical	0.0002	-3.73
	Typical Early Tau	Cognitive	0.016	2.45
	Typical Late Tau	Cognitive	<0.0001	4.07
	Cortical	Cognitive	<0.0001	5.51
	Cognitive	Subcortical	0.001	-3.38
	Cognitive	Subcortical	0.001	-3.38
**Executive Function Cognitive Battery (t-test)**				
	Normal Appearing	Typical Early Tau	<0.0001	12.83
	Normal Appearing	Typical Late Tau	<0.0001	10.61
	Normal Appearing	Cortical	<0.0001	7.68
	Normal Appearing	Cognitive	<0.0001	12.99
	Normal Appearing	Subcortical	<0.0001	6.16
	Typical Early Tau	Cortical	0.000	-3.73
	Typical Early Tau	Cognitive	0.016	2.45
	Typical Late Tau	Cognitive	<0.0001	4.07
	Cortical	Cognitive	<0.0001	5.51
	Cognitive	Subcortical	0.0012	-3.38
	Cognitive	Subcortical	<0.0001	-6.53
