# Supplementary material for: Multimodal subtypes identified in Alzheimer’s Disease Neuroimaging Initiative participants by missing-data-enabled subtype and stage inference
Source: Brain Commun. 2024 Jun 25;6(4):fcae219. doi: 10.1093/braincomms/fcae219 (PMC11259979; doi:10.1093/braincomms/fcae219)
Supplement: fcae219_Supplementary_Data [file Revision_1_ Response_to_reviewers_comments.pdf › Revision_1_ Response_to_reviewers_comments.pdf]

Thank you for your feedback and the provisional acceptance of our manuscript, "Multi-modal subtypes identified in ADNI by missing-data-enabled Subtype and Stage Inference," for publication in Brain Communications. We are grateful to the reviewers for their valuable insights and constructive comments, which have helped refine our work.

In response to the comments from the Scientific Editor regarding the statistical analysis in Figure 4, we have revised our approach. We now employ post-hoc p-value Bonferroni corrections to ensure a robust comparison of groups within our study, aligning with the journal's requirements. This change is reflected in the revised version of Figure 4 and its caption.

We are grateful for the guidance and support from the review team and look forward to our work being published in Brain Communications.

Reviewer(s)' Comments to Author:

Reviewer: 1

Comments to the Author

Thank you for the discussion. This study adds valuable findings to the AD subtypes' research field.

Thank you.

Reviewer: 2

Comments to the Author

The authors of the manuscript have addressed in a convincing way both the questions I had raised after the first review and the minor formal comments about exposition, notations, and plots. I believe the manuscript is now improved and worthy of acceptance to Brain Communications.

Thank you.

Comments from the Scientific Editor:

- Figure 4: we do not accept t-test or equivalent for comparing 2 groups when there are more than 2 conditions in the study. For this type of comparison, we suggest ANOVA, or linear models with post-hoc comparison or post-hoc p-value corrections (Holm, Bonferroni, etc).

Thank you for your valuable feedback regarding the statistical analysis approach used in Figure 4 of our manuscript. We have taken your advice into consideration and have revised it accordingly. The modifications include the application of post-hoc Bonferroni corrections for multiple comparisons, as suggested. Figure and caption have been updated to reflect this methodological adjustment (page 33-34 of revised manuscript)

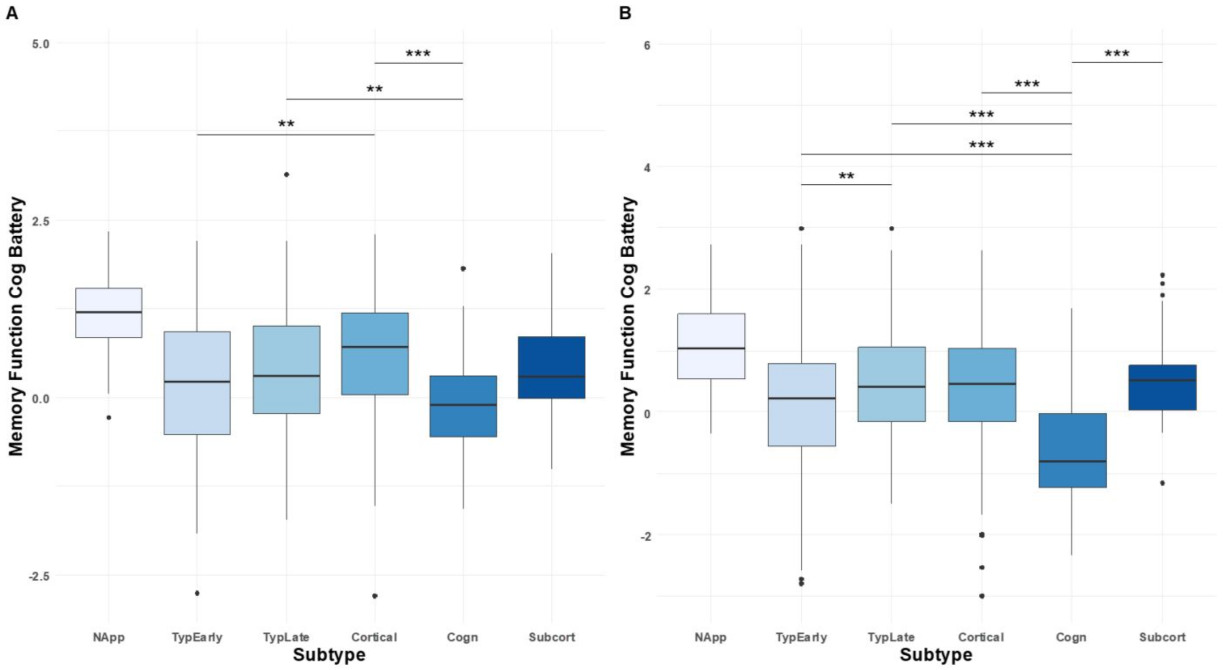

Figure 4. Boxplots showing subtype differences in two cognitive batteries. (A) Memory Function. Significant differences were found between 'Normal Appearing' vs. 'Typical Early Tau' (t = 12.83; p-value < 0.0001), 'Normal Appearing' vs. 'Typical Late Tau' (t-statistic = 10.61; p-value < 0.0001), 'Normal Appearing' vs. 'Cortical' (t = 7.68; p-value < 0.0001), 'Normal Appearing' vs. 'Cognitive' (t = 12.99; p-value < 0.0001), and 'Normal Appearing' vs. 'Subcortical' (t = 6.16; p-value < 0.0001). Significant differences were also noted between 'Typical Early Tau' and 'Cortical' (t = -3.73; p-value = 0.0012), 'Typical Late Tau' and 'Cognitive' (t = 4.07; p-value = 0.008), and 'Cortical' and 'Cognitive' (t = 5.51; p-value < 0.0001). **(B)** For Executive Function, the patterns were similar: 'Normal Appearing' vs. 'Typical Early Tau' (t = 9.61; p-value < 0.0001), 'Normal Appearing' vs. 'Typical Late Tau' (t = 6.49; p-value < 0.0001), 'Normal Appearing' vs. 'Cortical' (t = 5.98; p-value < 0.0001), and 'Normal Appearing' vs. 'Cognitive' (t = 11.70; p-value < 0.0001). Additional significant differences were observed between 'Typical Early Tau' and 'Typical Late Tau' (t = -3.70; p-value = 0.002), 'Typical Early Tau' and 'Cognitive' (t = 4.91; p-value = < 0.0001), 'Typical Late Tau' and 'Cognitive' (t = 7.62; p-value < 0.0001), 'Cortical' and 'Cognitive' (t = 6.55; p-value < 0.0001), and 'Cognitive' vs. 'Subcortical' (t = -6.53; p-value < 0.0001). All reported p-values are after Bonferroni correction for multiple comparisons. Significant correlations between 'Normal Appearing' and all other subtypes are not depicted in the figure for easier visualization. Subtypes abbreviations: NormalApp = Normal Appearing; TypEarlyT = Typical Early Tau; TypLateT = Typical Late Tau.
